# Genomic signatures of adaptive introgression from European mouflon into domestic sheep

**DOI:** 10.1038/s41598-017-07382-7

**Published:** 2017-08-08

**Authors:** Mario Barbato, Frank Hailer, Pablo Orozco-terWengel, James Kijas, Paolo Mereu, Pierangela Cabras, Raffaele Mazza, Monica Pirastru, Michael W. Bruford

**Affiliations:** 1 Istituto di Zootecnica, Università Cattolica del Sacro Cuore, via Emilia Parmense 84, Piacenza, Italy; 2 School of Biosciences, Cardiff University, CF10 3AX Cardiff, Wales UK; 3 CSIRO Agriculture, St Lucia, Brisbane, 4067 QLD Australia; 4 Department of Biomedical Sciences, and Centre for Biotechnology Development and Biodiversity Research, University of Sassari, V.le San Pietro 43, Sassari, Italy; 5 Istituto Zooprofilattico Sperimentale della Sardegna, Tortolí, Ogliastra Italy; 6 Laboratorio Genetica e Servizi - Associazione Italiana Allevatori, Cremona, Italy

## Abstract

Mouflon (*Ovis aries musimon*) became extinct from mainland Europe after the Neolithic, but remnant populations from the Mediterranean islands of Corsica and Sardinia have been used for reintroductions across Europe since the 19^th^-century. Mouflon *x* sheep hybrids are larger-bodied than mouflon, potentially showing increased male reproductive success, but little is known about genomic levels of admixture, or about the adaptive significance of introgression between resident mouflon and local sheep breeds. Here we analysed Ovine medium-density SNP array genotypes of 92 mouflon from six geographic regions, along with data from 330 individuals of 16 domestic sheep breeds. We found lower levels of genetic diversity in mouflon than in domestic sheep, consistent with past bottlenecks in mouflon. Introgression signals were bidirectional and affected most mouflon and sheep populations, being strongest in one Sardinian mouflon population. Developing and using a novel approach to identify chromosomal regions with consistent introgression signals, we infer adaptive introgression from mouflon to domestic sheep related to immunity mechanisms, but not in the opposite direction. Further, we infer that Soay and Sarda sheep carry introgressed mouflon alleles involved in bitter taste perception and/or innate immunity. Our results illustrate the potential for adaptive introgression even among recently diverged populations.

## Introduction

Introgression is increasingly documented as a potentially adaptive evolutionary force^1^, with recent developments in high-throughput genotyping and sequencing facilitating the detection of even small genomic regions that have been passed on from one taxon to another^2^. Since Darwin’s early work on domesticates, evolutionary biologists have devoted much attention to the relationships between domesticates and their wild ancestors. While much work has focused on describing the evolutionary consequences of domestication on modern sheep breeds^3^, less work has focused on their wild counterpart, the mouflon.

Sheep have a complex evolutionary history shaped by widespread extinctions in the wild, domestication, and feralisation. The European mouflon (*O. aries musimon*) is the only wild ovine currently occurring in Europe. Present in the archaeozoological record in Europe since the middle Pleistocene^4^, European mouflon went extinct across mainland Europe after the Neolithic^5^. Early agricultural societies then brought domesticated sheep into Europe during the Neolithic transition^6^. Archaeological evidence along with analysis of retroviral genomic markers in wild and domestic sheep breeds suggest two main domestication events: a first wave of domestication at around 11,000 years ago (YA), and a second wave around 6,000 YA^7, 8^. The European mouflon, together with the Cypriot mouflon (*O. orientalis ophion*) and some primitive domestic breeds present in Northern Europe such as the Soay and Spael sheep are considered remnants from the first wave of domestication^8^.

Humans translocated mouflon onto the Mediterranean island of Cyprus ~10,000 YA, and to Corsica and Sardinia ~6–7,000 YA^9^, where feral populations became established^7^. Since the late 18^th^ century, Corsican and Sardinian mouflon have been used to repopulate several regions of mainland Europe^10, 11^. Corsican mouflon were introduced as game and park animals in Southern France, and subsequently into other European countries^12^, whereas both Sardinian and Corsican animals were moved to central and northern Italy and Austria^13, 14^. Currently, continental European mouflon are distributed from the Iberian Peninsula to the Caucasus.

Feral mouflon populations of Sardinia, Corsica and Cyprus have coexisted with sheep populations since the arrival of the second wave of domesticated sheep. Even today, sheep herding practices in Sardinia involve seasonal transhumance from lower towards higher-altitude pastures, where mouflon reside and farmers habitually allow sheep to graze in the wild^12^. Records from ancient Rome and more recently from the 18^th^ century (both based on ref. 15) describe interbreeding between wild and domestic sheep in Europe. Mouflon and domestic sheep have therefore occurred in sympatry on several Mediterranean islands for millennia, and historical records indicate that admixture may be common^15^. Importantly, mouflon *x* sheep hybrids tend to be larger than mouflon^16^, and larger-bodied mouflon males have higher reproductive success^17^. Despite these records of mouflon *x* sheep admixture, and although sexual selection might act to enhance introgression into mouflon, little information is available on the scale, impact and adaptive significance of admixture between mouflon and domestic sheep.

Molecular approaches have been used to investigate the genetic structure of European and Mediterranean mouflon populations, including several phylogenetic studies with datasets comprising mouflon and domestic sheep^18–20^. Domestic Sardinian sheep and local mouflon show varying levels of admixture^12, 21^, and mtDNA cannot be used to effectively infer gene flow between them, as both mouflon and domestic breeds belong to the same mitochondrial haplogroup (B)^20^. The OvineSNP50 BeadChip (Illumina Inc.) includes 54,241 domestic sheep polymorphisms, and while originally developed to assess genetic diversity^22^ and perform genome-wide association studies^23^ in domestic sheep, it can also be used for other purposes such as studying conservation genetics^24^, domestication, local adaptation^25^, and admixture in wild and feral populations^26, 27^.

Here we used the OvineSNP50 BeadChip to analyse the most comprehensive genome-scale dataset of European mouflon to date. Our sampling covers the European mainland and Mediterranean islands, including Corsican, Corsican-derived (Spain, Hungary) and three Sardinian mouflon populations. Mouflon samples from Cyprus and Iran were also included, along with adjacent domestic breeds. We used this dataset to investigate the form, extent and potential adaptive significance of admixture between feral and domestic populations. We explored signals of local ancestry along sheep chromosomes, applying a novel approach to identify chromosomal regions of consistent ancient ancestry and to infer the direction of introgression (feral to domestic, or vice versa). We analysed sympatric mouflon and sheep populations from Sardinia to investigate the adaptive significance of introgression. We hypothesized that introgression from feral mouflon into recently imported local sheep breeds (Sarda) could have greater adaptive significance with regard to local environmental conditions, than introgression from domestic Sarda sheep into resident mouflon.

## Materials and Methods

All of the animal procedures were performed in strict accordance with the guidelines of the Ethics Committee of Sassari University, Italy, which also approved this study.

### Samples, DNA extraction and genotyping

**Figure 1:**
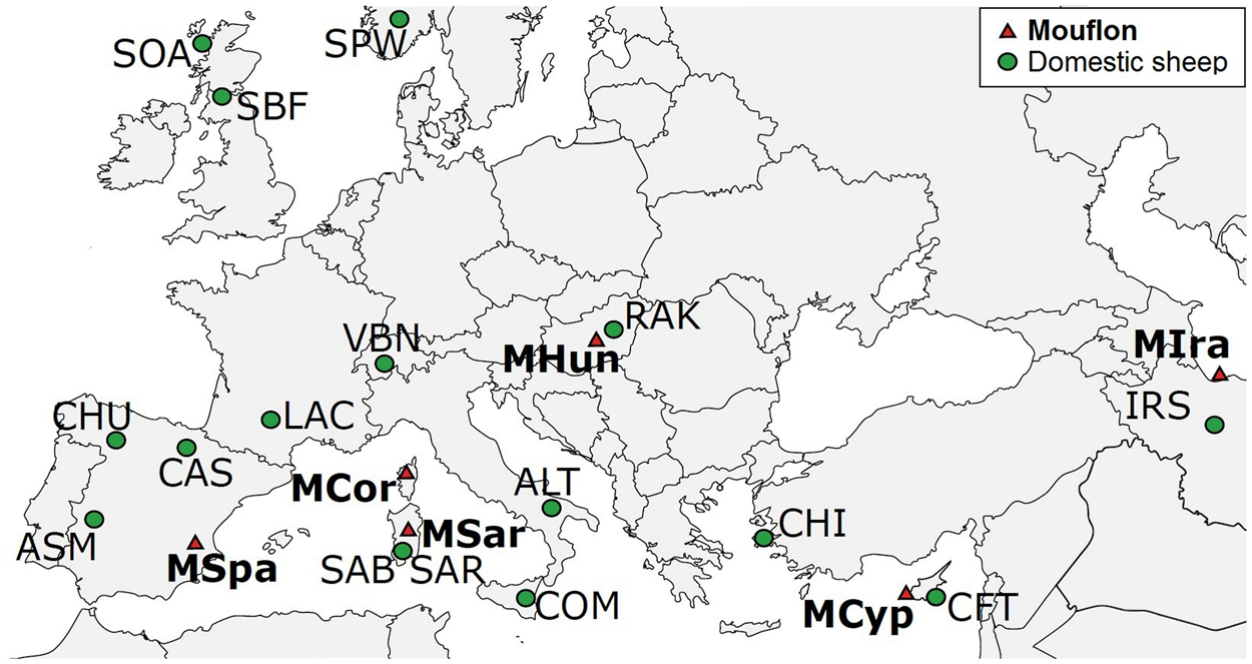
Sampling location. Geographic origin of mouflon and domestic sheep samples. For population abbreviations see Table 1. Map was generated in Inkscape v 0.91 (https://inkscape.org/).

We analysed 92 mouflon from eight populations across continental Europe, remnant populations on Mediterranean islands including Cyprus and three subpopulations from Sardinia, and from Iran (Fig. 1). For comparison, we collected data from 330 individuals from domestic European sheep breeds that either live in sympatry with or adjacent to mouflon, or have been generally described as ancient/autochthonous breeds. Other domestic sheep data were available from the Sheep HapMap project^22^ (Table 1; for additional details see Supplementary Text S1). Genomic DNA was obtained from blood and muscle tissue using phenol/chloroform extraction. Sample quality and concentration was determined via spectrophotometry using a ND-8,000 (NanoDrop Technologies, Thermo Fisher Scientific Inc., Wilmington, DE).

**Table 1:** Sample information and diversity indexes.

Breed/population	Acronym	Origin	Number	H_o_ (SD)	N_e_	F	Source
*Mouflon*							
Sardinian mouflon	MSar1	Sardinia	19	0.22 (0.19)	261	0.45	This study
Sardinian mouflon	MSar2	Sardinia	8	0.22 (0.24)	130	0.46	This study
Sardinian mouflon	MSar3	Sardinia	28	0.34 (0.19)	273	0.16	KJ^a^
Spanish mouflon	MSpa	Spain	21	0.20 (0.19)	96	0.51	KJ^a^
Hungarian mouflon	MHun	Hungary	8	0.24 (0.21)	282	0.42	This study
Corsican mouflon	MCor	Corsica	3	0.24 (0.27)	259	0.41	This study
Cypriot mouflon	MCyp	Cyprus	3	0.09 (0.20)	244	0.78	This study
Iranian mouflon	MIra	Iran	2	0.25 (0.31)	—	0.35	NG^b^
*Total*			*92*				
*Domestic sheep*							
Altamurana	ALT	Italy	24	0.37 (0.16)	628	0.06	KJ^a^
Australian Merino	ASM	Spain	24	0.37 (0.15)	920	0.06	KJ^a^
Castellana	CAS	Spain	23	0.38 (0.16)	813	0.02	KJ^a^
Chios	CHI	Greece	23	0.33 (0.17)	391	0.15	KJ^a^
Churra	CHU	Spain	24	0.37 (0.16)	617	0.05	KJ^a^
Comisana	COM	Italy	24	0.38 (0.16)	1028	0.03	KJ^a^
Cyprus Fat Tail	CFT	Cyprus	24	0.34 (0.19)	186	0.13	KJ^a^
Iranian sheep	IRS	Iran	6	0.37 (0.22)	412	0.05	NG^b^
Milk Lacaune	LAC	France	24	0.37 (0.16)	607	0.06	KJ^a^
Nera di Arbus sheep	SAB	Sardinia	20	0.36 (0.18)	366	0.08	KJ^a^
Racka	RAK	Hungary	8	0.35 (0.21)	327	0.11	This study
Sarda sheep	SAR	Sardinia	10	0.37 (0.19)	755	0.07	This study
Scottish Blackface	SBF	UK	24	0.37 (0.17)	428	0.05	KJ^a^
Soay	SOA	UK	24	0.27 (0.20)	179	0.32	KJ^a^
Spael-white	SPW	Norway	24	0.34 (0.18)	367	0.14	KJ^a^
Valais Blacknose sheep	VBN	Switzerland	24	0.31 (0.18)	306	0.22	KJ^a^
*Total*			*330*				

Breed/population name and the corresponding code used throughout the manuscript, the country of origin and the number of individuals analysed in this work are shown in the first four columns, along with the observed heterozygosity (*H*_*o*_) and its standard deviation (*SD*, in brackets), the effective population size (*N*_*e*_) and the inbreeding coefficient (*F*). ^a^KJ refers to Kijas *et al*.^22^. ^b^NG refers to the NextGen Consortium (FP7/2010–2014, grant agreement no 244356 - “NextGen”).

Samples were genotyped using the OvineSNP50 BeadChip in the ‘Laboratorio Genetica e Servizi’ (Cremona) or as part of the ISGC HapMap experiment^22^. Markers with a call rate <0.99 and minor allele frequency (MAF) <0.05 were excluded from all analyses. For the Ovine 50 k SNP array, no mouflon were included in the discovery panel^22^, implying ascertainment bias when applying it to mouflon^28^. We therefore pruned SNPs on the basis of linkage disequilibrium (LD) in the total dataset, as this approach has been shown to reduce the impact of ascertainment bias, allowing less biased comparisons among populations by preferentially reducing mean heterozygosity within the populations used during SNP discovery^22, 29^. LD pruning was performed using the indep-pairwise function in PLINK v1.7^30^, where SNPs with *r* ^2^ > 0.5 were removed from sliding windows of 50 SNPs and with 10 SNPs of overlap. Only autosomal markers were kept for analysis. After pruning for MAF and LD, 36,961 SNPs distributed across 26 chromosomes were retained for analysis.

### Genetic diversity and population structure

Heterozygosity values were calculated using custom scripts and the inbreeding coefficient (*F*) was estimated using PLINK. *N*_*e*_ was estimated with the software SNeP v1.1^31^. The software uses LD to infer *N*_*e*_ at different *t* generations in the past where *t* = *1/2c* and *c* is the distance between SNPs in Morgans (in this case assuming 100 Mb = 1 Morgan^22^). The following options were used: sample size correction for unphased genotypes, correction to account for mutation, and Sved & Feldman’s mutation rate modifier. The most recent estimate of *N*_*e*_ was taken for *c* calculated at 1 Mb^22^.

Maximum likelihood analysis of population structure was conducted using ADMIXTURE v1.23^32^. Clustering solutions for the whole dataset were calculated for *K* values from 2 to 24, the latter corresponding to the total number of sampled populations/breeds in our study. Additional Admixture analyses were performed after removal of highly inbred and/or divergent sheep populations, and using supervised ancestry assignments (Supplementary Text S1). A principal component analysis (PCA) was performed to investigate the ordinal relationships between populations and individuals, using flashpca v1.2^33^ with default settings. Neighbour-net graphs using Reynolds’ distances, calculated with a custom script, were generated using Splitstree v4.13.1^34^. The occurrence of admixture was further investigated using Treemix v1.12^35^. This software models the relationship among the sample populations with their ancestral population using genome-wide allele frequency data and a Gaussian approximation of genetic drift^35^. The *f* index representing the fraction of the variance in the sample covariance matrix ($\widehat{W}$) accounted for by the model covariance matrix ($W$) was used to identify the information contribution of each migration vector added to the tree. Up to 20 possible migration vertices were computed.

f3 and f4 admixture tests^36^ were performed using Treemix^37^. In the f3 test, the putative admixture of a target population (*A*) is tested against two source populations (*B*, *C*). A significant negative value of the resulting f3 score indicates *A* being the result of admixture of *B* and *C*. Similarly, the f4 test investigates the tree topology of four populations, with resulting f4 scores significantly different from 0 in cases of a distorted topology likely being due to admixture. Extreme positive scores suggest gene flow between *A* and *C* and/or *D* and *B* that surpasses any gene flow between *A* and *D* and/or *B* and *C*, whereas extreme negative scores suggest gene flow between *A* and *D* and/or *B* and *C* that surpasses that between *A* and *C* and/or *B* and *D*. To determine extreme f3/f4 values, a data normalisation and outlier detection approach was implemented (see Supplementary Text S1).

### Inference of local genomic ancestry (PCAdmix)

We used PCAdmix v1.0^38^ to infer local genomic ancestry. PCAdmix utilises haplotypes from ancestral representatives to infer ancestry of focal individuals. The software performs the inference chromosome-wide through PCA, via short windows along each chromosome. Using a hidden Markov Model, PCAdmix then returns the posterior probability (PP) of ancestry from each reference population for each haploid individual for each window. Additional information on PCAdmix parameters is available in Supplementary Text S1. PCAdmix requires phased genotypes, which we obtained using fastPHASE v1.2^39^. Default parameters were used in fastPHASE, except that we allowed for the incorporation of subpopulation labels, as this has been shown to significantly improve the imputation accuracy^40^.

To perform the local genomic ancestry analyses we used three reference populations: one population representative of the Sardinian mouflon lineage, one from the Corsican mouflon lineage, and one domestic sheep breed (see Supplementary Text S1 for details). Analyses were repeated using different domestic breeds as a third reference population, to assess how this choice affected the results. Additional analyses using a different combination of mouflon references were also performed (Supplementary Text S1).

### A novel approach to identify consistent genomic windows of introgression

We observed relatively high variation among PCAdmix results, when comparing a given focal population to different combinations of ‘pure’ reference breeds (see ‘Results’). We therefore developed a pipeline that uses a sliding-window approach to identify genomic regions that show consistent PCAdmix signals of introgression (i) in all individuals of the focal population, and (ii) across different reference population comparisons (PCAdmix runs). Specifically, multiple PCAdmix analyses are performed, each utilizing different reference populations. The results of these analyses are filtered for highly concordant introgression signals using a sliding-window approach along chromosomes that assigns a concordance score to each window. A concordant signal is one that appears across individuals of the focal population, and across multiple tests using different references (i.e. is not dependent on which reference population is used).

Chromosomal regions exhibiting concordance scores higher than a certain percentile of the genome-wide concordance score distribution are denoted as Consistently Introgressed Windows of Interest (CIWIs). Here we conducted the analysis based on both the 95^th^ and 99^th^ percentile. Additional information on the CIWI approach is available in Supplementary Text S1 and Supplementary Fig. S3.

### GO term identification

To identify GO terms significantly overrepresented in the CIWIs, all genes located inside or within 20 kbp (half median distance between two SNPs in the Ovine 50 k SNP chip) from the endpoints of each CIWI were compared against a background set of 11,089 genes, each containing or being in close proximity with a SNPs present in the 50 k SNP chip^22^. The comparisons were performed using GOrilla^41^, employing a false discovery rate (FDR) threshold of 0.05.

## Results

In total 422 individuals from seven mouflon and 16 domestic sheep populations were analysed at 36,961 SNP positions, after pruning for MAF and LD. Observed heterozygosity ranged from 0.09 to 0.34 for mouflon populations, with MSar3 showing the highest value and MCyp the lowest (Table 1). Heterozygosity for domestic sheep breeds was generally higher (range: 0.30 to 0.38), with most values overlapping with those reported previously for the same populations^21, 22^. Effective population size (*N*_*e*_) values of most mouflon populations were around 250, with the highest value recorded for MHun (282) and the lowest for MSpa (96). *N*_*e*_ values for domestic sheep were generally comparable with those from previous studies^21, 22^, showing differences in *N*_*e*_ < 50, with the exception of ALT, COM, CAS where the difference in *N*_*e*_ was >100. Estimated inbreeding values ranged around 0.40–0.45 in most mouflon populations, although a lower value was recorded for MSar3 (0.16) and the largest was recorded for MCyp (0.78). The inbreeding values for most domestic sheep populations were low (0.02–0.15); larger values were recorded for VBN (0.22) and SOA (0.32) in accordance with previous observations^22^.

### Population structure and genome-wide signals of admixture

**Figure 2:**
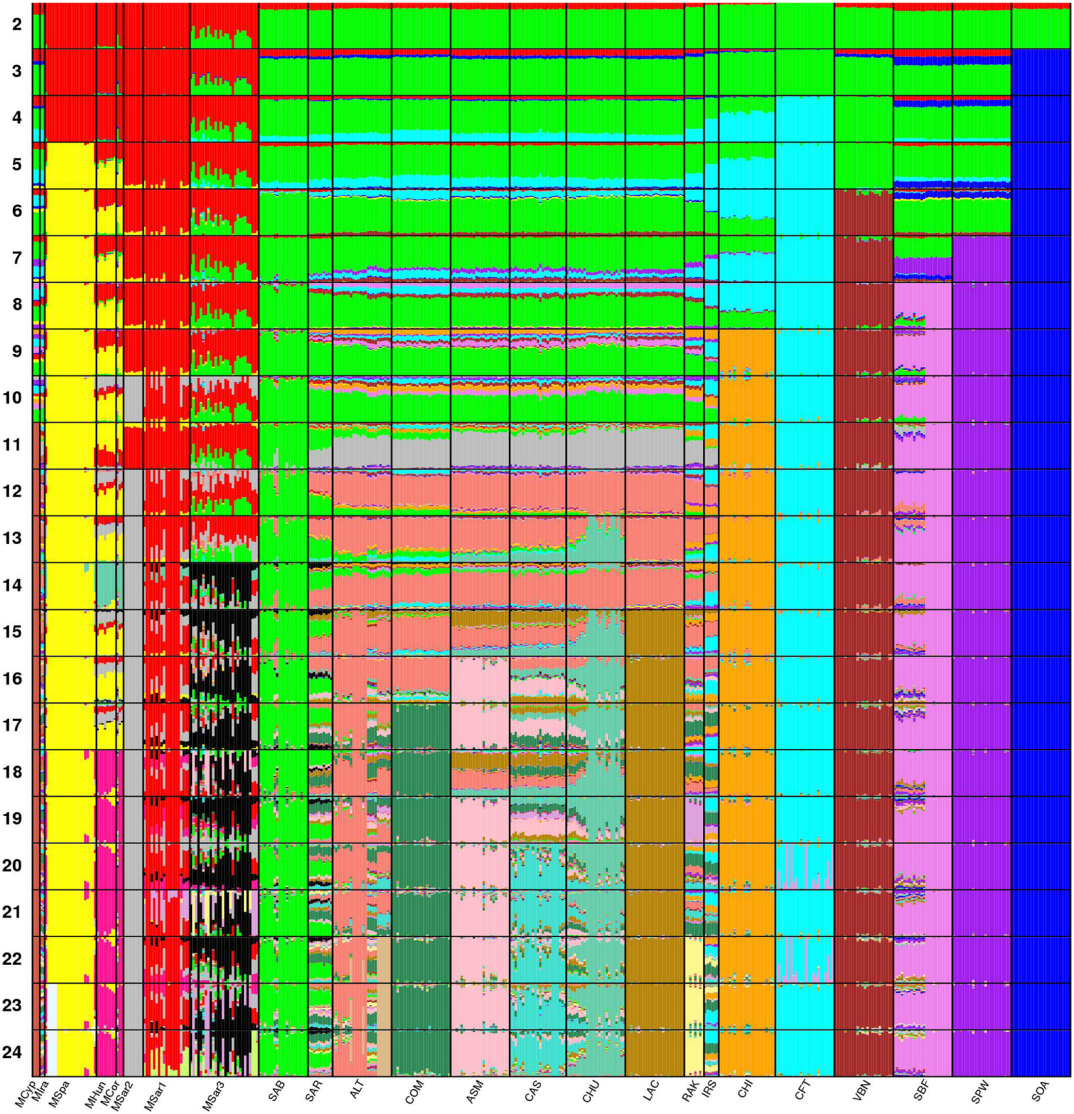
Admixture plot. Admixture plot comprising the first 24 clustering solutions of all the 422 individuals analysed in this work. The analysis is based on 36,961 SNPs from the Ovine SNP50BeadChip. For population abbreviations see Table 1.

Results from (unsupervised) Admixture analysis at *K* = 2 clustered the samples into relatively distinct domestic sheep and mouflon groups, both when using the full dataset, and after removal of the most inbred and highly divergent populations (Fig. 2, Supplementary Fig. S1). Extensive signals of admixture were discernible in 24 individuals of the MSar3 population, showing 21–51% of sheep assignment, as well as in one individual of the MCor population. The eastern MCyp and MIra populations showed ~74% cluster membership consistent with domestic assignment. Otherwise, low admixture proportions (~5%; Fig. 2) were found in most mouflon. Also domestic breeds showed introgression, displaying on average 14% of mouflon cluster membership (Fig. 2). An exception were Eastern Mediterranean and SW Asiatic breeds (IRS, CHI and CFT), for which the mouflon component was <5%. At *K* = 5, a cluster restricted to mouflon derived from Corsican stocks (MCor, MHun and MSpa) was detected, which was absent from other mouflon populations in Sardinia and other regions. At *K* = 11, MCyp was detected as a distinct cluster, as was MSar2 at *K* = 12, while the Corsican and Hungarian mouflon formed a distinct cluster at *K* = 18. A supervised Admixture analysis (Supplementary Fig. S1) identified the same overall pattern of bidirectional sheep/mouflon introgression, but clustered SOA with MHun: a pattern not seen in any of the other analyses. Supervised and unsupervised clustering analyses were congruent, however, once SOA and other inbred and/or heavily inbred populations were removed (Supplementary Fig. S1).

In the PCA (Supplementary Fig. S4), the first principal component (PC) accounted for 7.3% of the variance and discriminated sheep and European mouflon, mirroring the admixture results obtained at *K* = 2. The second PC accounted for 3.4% of the variance and reflected Admixture results for *K* = 3, discriminating northern sheep breeds from other domesticates. The third and fourth PCs split the Sardinian and Corsican mouflon and the Asiatic and European sheep breeds respectively. The Neighbour-net analysis of pairwise Reynolds’ distances between sampled populations (Supplementary Fig. S5) clearly differentiated mouflon from domestic sheep. The European mouflon occupied a separate branch and was further split into the Corsican and Sardinian lineages. Separate branches differentiated the North European domestic sheep breeds, the two Sardinians and the breeds from the East Mediterranean.

**Figure 3:**
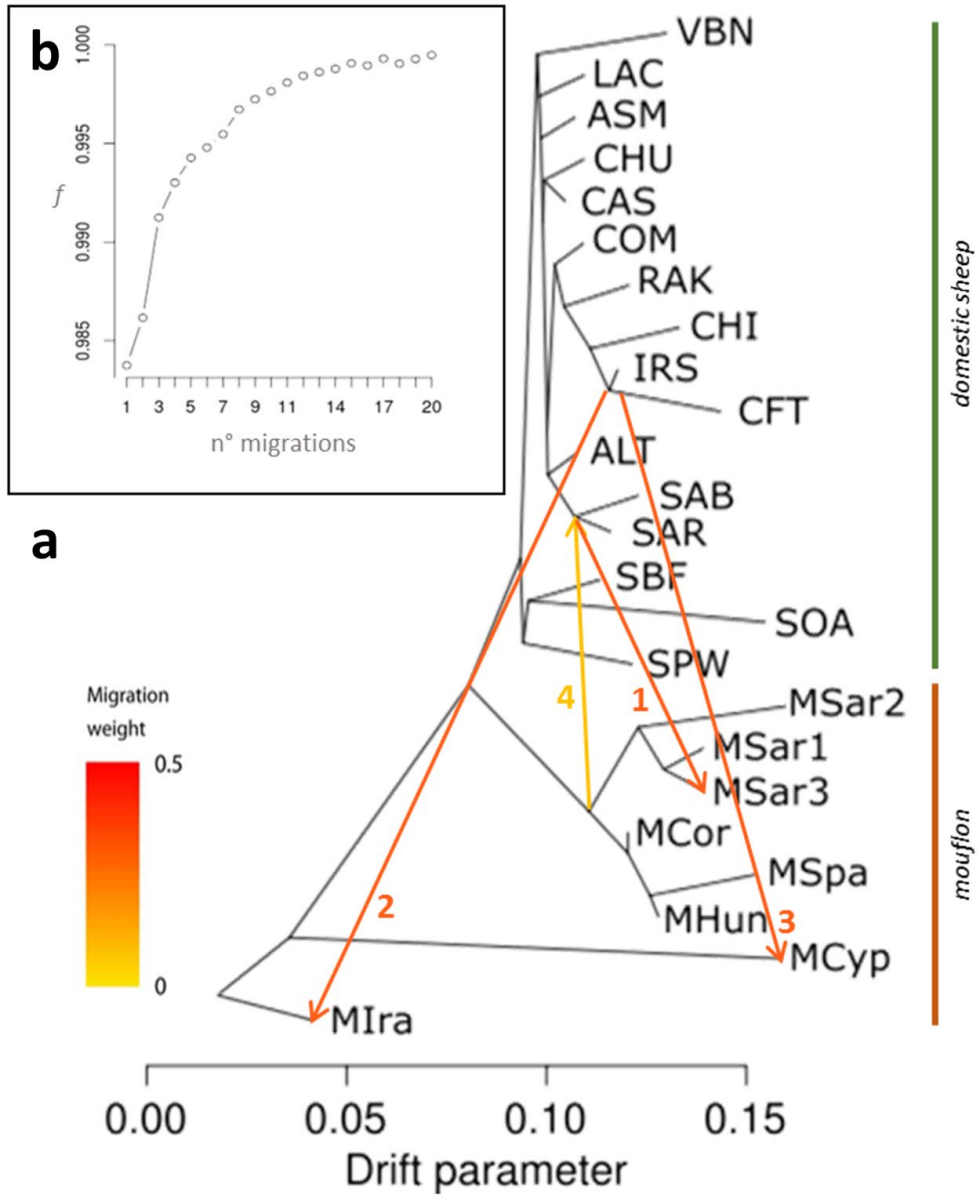
Treemix plot. (**a**) Phylogenetic network inferred by Treemix of the relationships between mouflon and domestic sheep populations. The first four migration edges between populations are shown with arrows pointing in the direction toward the recipient group, and coloured according to the ancestry percentage received from the donor. The numbers associated with each vector identify the identification order. (**b**) The inset shows the *f* index representing the fraction of the variance in the sample covariance matrix ($\widehat{W}$) accounted for by the model covariance matrix ($W$), as a function of the number of modelled migration events. For population abbreviations see Table 1.

Maximum likelihood assessment of population history with overlaid admixture events using Treemix (Fig. 3, Supplementary Fig. S6) confirmed several aspects already detected by Admixture (Fig. 2). The first four migration edges (gene flow events) accounted for more than half of the total model significance explained by the *f* statistic, with the first migration edge having an *f* value of 0.98. Vectors from 9 to 20 brought only a small increase in *f* value (<0.001) and migration weights close to 0 (Fig. 3, inset). The first four vectors all indicated gene flow between sympatric mouflon and sheep (Fig. 3): vectors 1–3 denote gene flow from domestic sheep into mouflon on Sardinia, Iran and Cyprus, mirroring *K* = 2 results from Admixture (Fig. 2). The fourth Treemix vector connected European mouflon to Sardinian domestic sheep breeds (SAR and SAB), indicating bidirectional gene flow. All 12 significant f3 test results confirmed the admixture of MSar3 (Supplementary Table S1). The two most extreme f3 scores (−25.43 and −22.12) were assigned to the geographically proximate populations of MSar1/SAR and MSar1/SAB as sources of admixture for MSar3, respectively. No significant result was obtained for any f4 test.

### Inferring sheep versus mouflon ancestry of specific genomic locations

The observed widespread signals of genomic admixture prompted us to use PCAdmix to identify specific introgressed genomic regions. Graphical representations of all results are available as supplementary material (Supplementary Fig. S7).

MSar3 was the mouflon population with the highest proportion of its genome assigned to domestic sheep (30.6%), close to the mean estimate obtained from Admixture at *K* = 2 (29%). The average proportion of genomic regions assigned to domestic sheep for MSar1 and MSpa was 10.8% and 4%, respectively. Both estimates were larger than those obtained from Admixture, which identified ~1% sheep component in both mouflon populations. The highest PCAdmix component of MSar1 was assigned to Sardinian mouflon (26.3% assigned to MSar2; Table 2), while Corsican mouflon were assigned with 48.3% to MHun. The proportion of non-assigned regions (i.e. with a posterior probability (PP) smaller than 0.95) were similar for MSar1 and MSar3 (42.6% and 41.1% respectively), and the proportions of Sardinian and Corsican ancestry in MSar3 were each ~10 percentage points lower than for MSar1.

**Table 2:** Genome-wide local ancestry assignment values from PCAdmix.

		Reference populations			
		Sardinian mouflon	Corsican mouflon	Domestic sheep	PP < 0.95
**Focal population**	*Mouflon*				
	MSar1	26.3 ± 0.40	20.4 ± 3.26	10.8 ± 1.74	42.6 ± 1.84
	*MSar3	16.5 ± 0.59	11.5 ± 2.24	30.6 ± 3.49	41.4 ± 0.75
	MSpa	16.8 ± 2.19	48.3 ± 5.50	4.0 ± 0.29	30.9 ± 3.21
	*Domestic sheep*				
	ALT	1.5 ± 0.85	2.6 ± 2.69	82.3 ± 11.43	13.6 ± 7.89
	CHI	1.4 ± 1.16	2.2 ± 2.87	85.8 ± 12.47	10.6 ± 8.45
	CHU	1.7 ± 1.01	2.8 ± 3.03	82 ± 12.68	13.5 ± 8.68
	COM	1.7 ± 0.92	2.5 ± 2.69	82.3 ± 11.47	13.5 ± 7.91
	CFT	1.0 ± 0.73	2.3 ± 3.17	87.7 ± 11.06	9.0 ± 7.16
	RAK	1.3 ± 0.60	2.3 ± 2.82	86.2 ± 10.74	10.2 ± 7.35
	SAR	1.5 ± 0.67	2.1 ± 2.21	85.5 ± 8.22	11.0 ± 5.34
	SBF	1.6 ± 0.84	3.3 ± 3.61	80.8 ± 12.86	14.3 ± 8.42
	SOA	2.3 ± 0.63	4.1 ± 3.37	80.0 ± 10.24	13.6 ± 6.26
	SPW	1.7 ± 1.05	3.3 ± 3.62	81.0 ± 12.76	14.1 ± 8.10
	VBN	1.5 ± 0.88	2.6 ± 3.17	84.4 ± 12.07	11.5 ± 8.03

Shown is the average proportion of genome assigned by PCAdmix with posterior probability ≥ 0.95 to Sardinian mouflon, Corsican mouflon and domestic sheep, respectively. PP < 0.95 denotes the cumulative genome proportion remaining unassigned with posterior probability < 0.95. Averages were calculated across four reference sets (detailed in S5 Table), each comprising the same mouflon references (MSar2 and MHun for Sardinian and Corsican mouflon, respectively) and four different domestic sheep breeds (CAS, ASM, LAC and SAB_p). An asterisk highlights the mouflon population (MSar3) that presents a higher sheep genetic component than mouflon. For population abbreviations see Table 1.

For domestic sheep, the average proportion of the genome assigned by PCAdmix with PP ≥ 0.95 to the domestic gene pool was 83.5% (range 61.9–94.7%; Supplementary Table S2), a value comparable to Admixture results for *K* = 2, showing an average of 88% for the same populations. The highest and lowest mouflon admixture proportions from PCAdmix were consistently recorded by SOA and CFT respectively (Table 2). A marked difference between mouflon and domestic sheep in the certainty of assignment was recorded, with mouflon showing a much higher proportion (~39%) of non-assigned regions (PP < 0.95) than domestic sheep (~12%).

**Figure 4:**
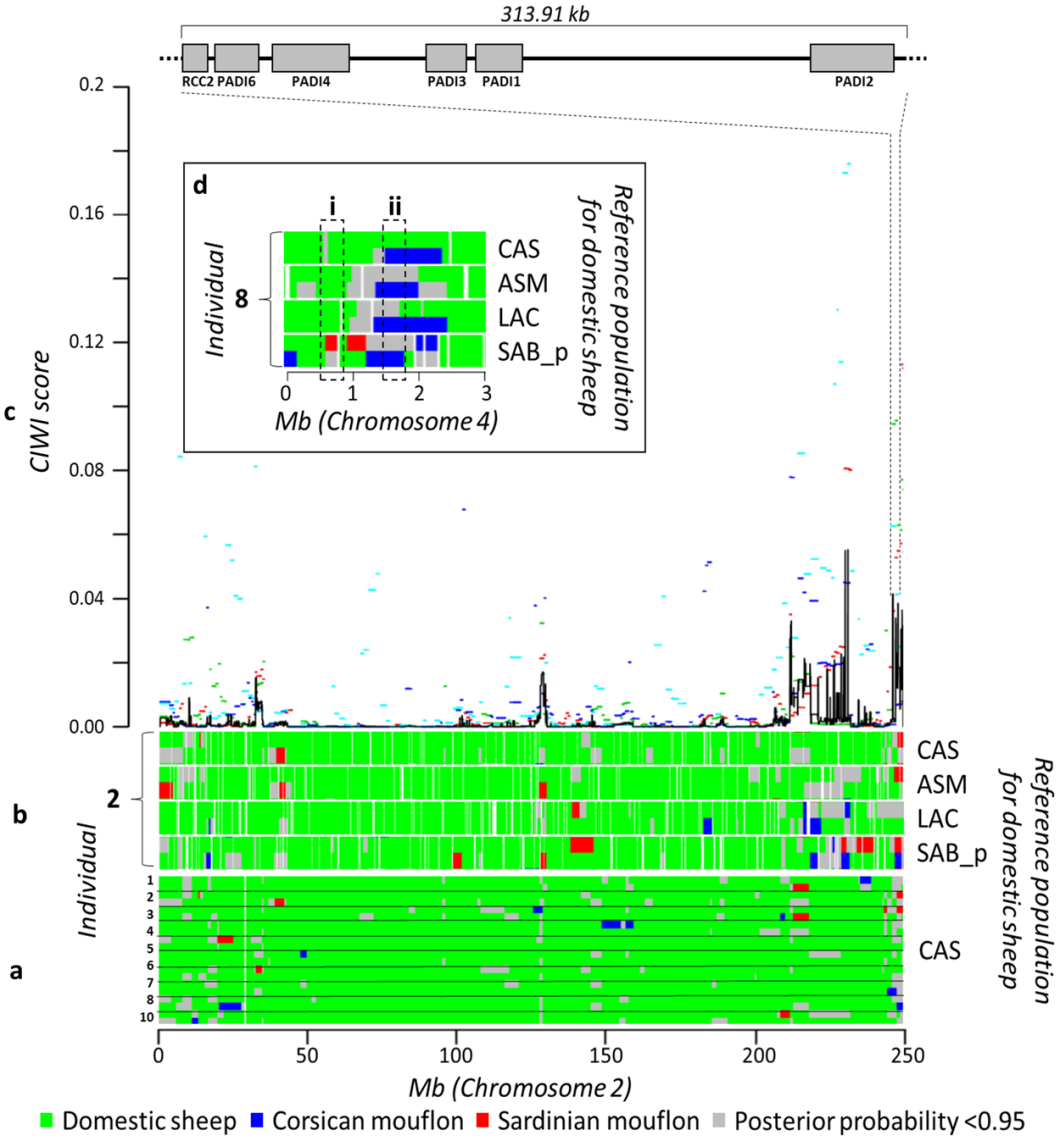
Graphical exemplification of the CIWI approach. Graphical representation of the inferred local ancestry for a domestic sheep breed (SAR) according to PCAdmix. (**a**) The 10 focal diploid individuals belonging to SAR are represented by the 10 numbered lines. Each line represents a diploid individual of the SAR population, and extends for the total length of the ovine chromosome 2 (249.99 Mb). The colour scheme indicates the assignment of each block to one of the three reference populations. (**b**) PCAdmix results for one individual (number 2) of the SAR population, obtained from comparison with four different reference populations for domestic sheep (CAS, ASM, LAC, SAB_p). Genomic regions not analysed by the software due to the absence of SNPs are visible as white gaps. (**c**) Within-analysis concordance scores (A-scores) are calculated along the chromosome to represent the concordance of ancestry assignment among the 10 focal SAR individuals. The A-scores relative to the four reference populations are represented by coloured segments. The CIWI score is then calculated from the A-scores and is represented by the black solid line. The inset expands the genomic region within chromosome 2, where genes related to the citrullination function are located. (**d**) Comparison between PCAdmix results for a portion of chromosome 4 in the same individual when compared to four different domestic sheep reference populations. Regions of discordance (i) and concordance (ii; a CIWI) are highlighted by dashed boxes.

PCAdmix therefore provided estimates of genome-wide admixture proportions for mouflon and domestic sheep that were consistent with results from Admixture. However, PCAdmix results for particular genomic regions – the main goal of using this approach - showed inconsistency with regard to the location and length of inferred admixture regions (Fig. 4-d, Supplementary Fig. S7, S8): with the same genomic region being assigned to either mouflon or sheep ancestry, depending on the reference population used in PCAdmix. Consequently, we developed a consensus approach that jointly analysed the results obtained with the four different domestic sheep reference populations, and across all analysed individuals from the focal population, highlighting genomic regions that, independently of the reference population used, showed highly consistent signals of introgression (Fig. 4-c). The CIWI regions obtained with this approach were then crosschecked against all genes covered by (or adjacent to) SNPs on the ovine 50 k BeadChip. On average across mouflon populations, our method identified introgression signals for 524 genes associated with 2,044 CIWIs when using the 95^th^ percentile confidence threshold, and 116 genes associated with 397 CIWIs for the 99^th^ percentile threshold. Across domestic sheep, a larger number of CIWIs were identified, with on average 996 genes located in 3,205 CIWIs, or 253 genes in 625 CIWIs (based on confidence thresholds of the 95^th^ and 99^th^ percentile, respectively; Supplementary Table S3).

### GO term analysis of introgressed loci

Genes identified within CIWIs in MSar1, MSar3 and MSpa showed no significant enrichment of any gene ontology (GO) terms (FDR ≈ 1), independent of the percentile threshold used. Conversely, seven GO terms associated to mouflon ancestry were identified in domestic breeds. All domestic breeds except SOA and SPW were enriched for GO terms involved in protein citrullination (Supplementary Table S4) whereas in both SAR and SOA, GO terms involved in the perception of bitter taste were significantly enriched (Supplementary Table S4). The genes involved in protein citrullination were located in four distinct chromosomal regions: two on chromosome 2, and one each on chromosomes 3 and 25 (Table 3). The genes related to bitter taste perception were located on chromosomes 2, 4 and 16 (Table 3). Similar results were obtained when the CIWI approach was applied to the extended Sardinian mouflon reference using both MSar1_p and MSar2 (resulting in eight ancestry analyses to be compared: two Sardinian mouflon and four domestics sheep reference populations; Supplementary Text S1). However, in this case no significant enrichment of GO terms related to bitter taste perception was detected (Supplementary Table S4).

**Table 3:** Genes with signals of adaptive introgression from mouflon into domestic sheep.

GO class	Gene	Chr	Position (Mb)
*Citrullination*	CPS1 - carbamoyl-phosphate synthase 1	2	211.16–211.3
	ATIC − 5-aminoimidazole-4-carboxamide ribonucleotide formyltransferase/imp cyclohydrolase	2	216.27–216.3
	PADI6 - peptidyl arginine deiminase, type vi	2	248.06–248.07
	PADI4 - peptidyl arginine deiminase, type iv	2	248.08–248.11
	PADI3 - peptidyl arginine deiminase, type iii	2	248.15–248.16
	PADI1 - peptidyl arginine deiminase, type i	2	248.17–248.19
	PADI2 - peptidyl arginine deiminase, type ii	2	248.31–248.35
	MAT2A - methionine adenosyltransferase ii, alpha	3	57.23–57.24
	MAT1A - methionine adenosyltransferase i, alpha	25	35.29–35.33
*Bitter taste detection*	TAS1R2 - taste receptor, type 1, member 2	2	246.63–246.65
	TAS2R3 - taste receptor, type 2, member 3	4	104.79–104.79
	TAS2R4 - taste receptor, type 2, member 4	4	104.81–104.81
	TAS2R38 - taste receptor, type 2, member 38	4	104.95–104.96
	PIP - prolactin-induced protein	4	105.91–105.92
	TAS2R39 - taste receptor, type 2, member 39	4	106.01–106.01
	TAS2R40 - taste receptor, type 2, member 40	4	106.07–106.07
	TAS2R1 - taste receptor, type 2, member 1	16	63.38–63.38

Genes identified by the CIWI approach, involved in either citrullination or bitter-taste detection (GO class). The chromosome (Chr) and physical position are shown.

## Discussion

We analysed patterns of global and local introgression between European mouflon and domestic sheep. We further investigated the potential adaptive nature of such introgression, since this process might provide a mechanism by which hardy, extensively managed sheep breeds can survive in challenging environments. For the first time to our knowledge, local introgression approaches were applied to genome-wide data in feral and domestic sheep. We found that signals of domestic sheep introgression into mouflon were strongest for one enclosed mouflon population in Sardinia (MSar3), likely resulting from extensive recent crossbreeding. Signals of sheep introgression into other European mouflon populations were generally weaker, with signal strength varying depending on the analysis approach used. We found that putatively introgressed genomic regions in mouflon were not systematically enriched for particular GO terms, while introgressed regions in domestic sheep were enriched for genes related to innate immunity (for most sheep breeds) and bitter taste recognition (for sheep breeds with broad dietary preferences). These results suggest that adaptive introgression has occurred from mouflon into domestic sheep, but not vice versa.

### Levels of genetic variability

Estimates of genetic diversity were similar for most previously studied mouflon populations, although lower variability and higher inbreeding levels were found for mouflon on Cyprus, indicating strong genetic drift and inbreeding. In contrast, one mouflon population from Sardinia (MSar3) showed higher genetic variability and lower inbreeding (Table 1), likely a consequence of introgression of domestic sheep alleles.

Mouflon showed lower heterozygosity, higher inbreeding and lower *N*_*e*_ than domestic sheep breeds. Ascertainment bias may contribute to this observation^29, 42^, given that mouflon were not part of the panel of individuals included when selecting the markers for the OvineSNP50 BeadChip. While this complicates direct comparisons of observed heterozygosity between mouflon and domestic sheep, comparisons within groups (e.g. among mouflon populations) are affected to a lesser extent. Furthermore, ascertainment bias can be alleviated by pruning data for high levels of LD^22^, and by using multilocus or haplotype-dependent analyses that are less affected by ascertainment bias than single locus statistics^42–44^. Hence, here we removed loci with high levels of LD and carried out both multilocus (e.g. Admixture) and haplotype-based (e.g. PCAdmix) analyses. Furthermore, we note that previous studies comparing microsatellite variability of domestic sheep and European mouflon showed the same trend as our SNP chip data, with mouflon populations showing lower heterozygosity than domestic sheep^45–47^. While also microsatellites can be affected by ascertainment bias^48^, their high mutation rates reduce the effect of the bias in comparison to that in SNP data. Our findings therefore suggest that, despite impacts of SNP chip ascertainment bias, European mouflon harbour clearly lower levels of genetic diversity than their domestic counterparts.

The analysed mouflon populations have presumably all passed through several population bottlenecks^14, 49^, although no detailed population history information is available for the Iranian population. These bottlenecks would have reduced genetic variability in mouflon. Furthermore, while many sheep farmers use several rams to sire flocks, breeding practices in dogs, cattle and horses often involve the use of popular sires that are bred to father a large number of offspring^50–54^. Sheep breeding practices, particularly in extensively managed populations, are therefore likely to contribute to the observed higher variability in sheep than in mouflon.

### Admixture between mouflon and domestic sheep

Similar to the findings of Lorenzini *et al*.^12^ who documented the presence of second-generation crossbred or backcrossed Sardinian mouflon sampled in the “Ogliastra” and “Nuoro” provinces, we found concordant signals of recent introgression for MSar3. Admixture, Treemix, and f3 test results suggest that most MSar3 individuals are admixed, with Sardinian sheep as putative introgression source. The MSar3 population was sampled in an enclosure, where mouflon were reared together with crossbred animals (Antonello Carta, *personal communication*). Our results indicate that this population, which so far has been used as a representative of Sardinian mouflon at large^21, 55^ is not representative of pure European mouflon, and that other mouflon population we studied may be more suitable as reference populations. We caution, however, that data from extinct continental European mouflon populations may be necessary to accurately quantify introgression of domestic sheep alleles into extant mouflon.

A large genomic component (~75%; Fig. 2) assigned by Admixture to domestic sheep was also found in the two *O. orientalis* populations MCyp and MIra, a pattern also visible in the PCA (Supplementary Fig. S4). However, Neighbour-net clearly separated MIra from IRS (Supplementary Fig. S5), arguing against a substantial contribution of domestic sheep to MIra. These results are most likely an effect of ascertainment bias, as *O. orientalis* are the most divergent populations with respect to the domestic sheep (i.e. the species for which the SNP chip was designed)^49^. We anticipate that genome sequencing and ascertainment bias-free characterization of genetic diversity will be important in ovine lineages that are more divergent from domestic sheep, such as mouflon from Cyprus and Iran.

Low levels of sheep introgression into mainland European mouflon may be explained by current mouflon management. On the mainland, mouflon populations derive from recent introductions (e.g. Hungary, Romania and Spain). These populations are kept as game for hunters and therefore either confined in enclosures, partially managed or monitored, potentially reducing the chances of crossbreeding. Additionally, sheep *x* mouflon hybrids tend to deviate phenotypically from mouflon (e.g., white fleece patches, woolly coat) and are in some areas actively removed from the wild gene pool by the hunting community^56^. In conclusion, mouflon-sheep hybrids are rare throughout Europe, and might in fact be actively selected against by humans.

Large effective size of historic mouflon populations may have limited the impact of any introgression from sheep. Cetti (1774) described Sardinian mouflon flocks each comprising hundreds of animals, much larger than the currently typical group sizes of less than ten individuals^57^. Furthermore, rarity of sheep introgression into mouflon could result from natural selection, with hybrid fitness being reduced under feral conditions, as recorded for wolf *x* dog hybrids^58^. Indeed, none of the regions in the mouflon genome that we infer to have domestic sheep ancestry shows any significant enrichment of GO terms, whether in the wild or in enclosures (MSar3). This is consistent with Corsican and Sardinian mouflon being adapted to local environmental conditions when domestic sheep arrived on the islands. Under this scenario, introgression of alleles from not locally adapted domestic sheep into resident mouflon might not have been favoured by natural or sexual selection. Hence, our results imply that hybrids – despite their larger body size and hence potentially increased reproductive success^16, 17^ – do not seem to have a larger reproductive success in the wild than purebred mouflon.

Most sheep breeds showed a small percentage of contribution from the mouflon cluster at *K* = 2 (Fig. 2). Among these, all SAR individuals showed similar amounts of Sardinian mouflon ancestry (at *K* = 24; Fig. 2). Additional support came from the Treemix analysis, which shows a migration vector starting from the root of Sardo-Corsican mouflon and ending at the root of the two Sardinian sheep breeds (Fig. 3). These results likely represent ancient admixture events.

While the Admixture and PCAdmix results mostly overlapped qualitatively, introgression estimates did not always coincide quantitatively; e.g. we inferred domestic ancestry in MSar1 of 1% or 11% with Admixture and PCAdmix, respectively (Fig. 2 and Supplementary Table S2). We attribute such discrepancies either to one or a combination of: (i) differences in assumptions/implementations between the two models, (ii) inaccuracies in gene flow estimation resulting from inferring past gene flow based on present populations rather than having ancient DNA data from the actually hybridising populations, and (iii) to biases resulting from phasing of SNP chip genotypes^38^. While the second issue requires sequence data from ancestral mouflon and sheep, the recently developed high-density Ovine HD BeadChip^59^ will provide more accurate phased data for chromosome painting analyses. Caution is therefore required in interpreting our introgression results quantitatively.

### Signals of adaptive introgression

Applying our consensus approach to identify CIWIs and associated genes in domestic sheep, we found mouflon ancestry in genomic regions related to the citrullination process. Citrullination enzymes such as PAD4 are essential in triggering the antibacterial innate immunity response known as neutrophil extracellular traps (NETs)^60^, with histone citrullination as the first step leading to NET assembly^61, 62^. Citrullination also plays a major role in bacteria-dependent inflammatory response in livestock, and enzymes responsible for this process are overrepresented in mastitic Sarda sheep milk^63^. We hypothesise that introgressed mouflon-derived alleles could have been positively selected in domestic sheep, because of fitness effects of higher plasticity of the antibacterial innate immunity provided by NETs. This adaptive introgression from mouflon into sheep could have helped translocated - and thus not necessarily locally adapted - domestic breeds cope in their novel environments.

We also found evidence of enrichment in GO terms related to bitter taste perception in Soay and Sarda sheep. Bitter taste perception is an important trait in ruminants, likely related to the avoidance of toxins and harmful substances in the diet^64, 65^. However, in domestic sheep, perception of bitter taste in a food item does not correlate with its rejection, probably reflecting a trade-off between toxicity avoidance and dietary plasticity^64, 66^. Although taste receptors are mostly located in the tongue, they can also be expressed in other tissues^67^. The function of the majority of these extraoral receptors is unknown, although bitter and sweet receptors in the airways have been linked to innate immunity functions^67^. Soay sheep live under particularly low management conditions^68^, which could explain the selective advantage of an introgressed genetic material from mouflon related to bitter taste perception. Similarly, the Sarda sheep is known to have broad dietary preferences and is characterised by primitive management practices. These aspects could explain why mouflon-derived bitter taste genes proved adaptive in these breeds, regardless whether the involved genes are ultimately related to bitter taste perception or to innate immunity-derived functions.

When including additional Mouflon reference populations, our results generally remained unchanged, although fewer remaining CIWIs were identified. We still recovered signals of adaptive introgression of mouflon-derived protein citrullination functions into domestic breeds. The bitter taste perception signal, however, was no longer significant for SAR and SOA sheep. This finding reflects the typical balance between type-1 and type-2 error in statistical inference, where reducing the false positive rate (as done here by increasing the number of comparisons) simultaneously increases the false negative rate. Despite the power inherent in large data sets, even when analysed with advanced evolutionary models, signals of (adaptive) introgression and incomplete lineage sorting can remain difficult to discriminate^1^. It is therefore possible that domestic sheep from the first wave of domestication such as SOA^8^ might have retained ancestral alleles at bitter taste genes, rather than having acquired them due to adaptive introgression from mouflon. Data polarisation using ancient (pre-split) genomic data could shed light on these scenarios^1^, as well as future attempts to date the putative introgression event through demographic inference.

### Conclusions

We have developed an approach to identify genomic regions that show consistent introgression signals (CIWIs), based on mid-density SNP array data from multiple reference populations and focal individuals. Due to its requirements of high stringency built in to reduce the occurrence of false positives, our strategy is prone to generate false negative results (increasingly so, when more sets of results are compared, see Supplementary Table S4). We also note that our approach focuses on introgression signals that are close to fixation in the receiving population, so cases where introgressed material is more rare might remain undetected^69^.

Despite millennia of coexistence of mouflon and domestic sheep, our findings indicate that – despite some signals of bidirectional introgression – only very limited *adaptive* introgression from domestics into the wild has occurred. Given expected better local adaptation of mouflon compared to recently domesticated and typically geographically translocated domestic breeds, this finding is not unexpected. Conversely, introgression from wild mouflon into sympatric domestics seems to have been favoured by positive selection. Adaptive introgression of mouflon alleles may be explained by limited local adaptation in domestics. Specifically, we here show that genes with functions related to innate immunity and bitter taste have been introgressed into numerous sheep breeds, putatively allowing them adapt to local parasite/disease pressures, and perhaps aiding in the utilization of local food resources.

### Data accessibility

All relevant data and scripts are within the paper and its Supporting Information files.

## Electronic supplementary material

Supplementary Information

Supplementary data
