# Supplementary material for: Genomic signatures of adaptive introgression from European mouflon into domestic sheep
Source: Sci Rep. 2017 Aug 8;7:7623. doi: 10.1038/s41598-017-07382-7 (PMC5548776; doi:10.1038/s41598-017-07382-7)
Supplement: Supplementary file 1 — Supplementary Information [file 41598_2017_7382_MOESM1_ESM.pdf]

Genomic signatures of adaptive introgression from European mouflon into domestic sheep

Mario Barbato, Frank Hailer, Pablo Orozco-terWengel, James Kijas, Paolo Mereu, Pierangela Cabras, Raffaele Mazza, Monica Pirastru and Michael W. Bruford

Index

S1 Text. Supplementary materials and methods....2

Sample information....2

Population structure analyses....2

PCAdmix parameters.....3

Consensus approach....3

S1 Table. f3 tests most significant results....5

S2 Table. Genome-wide local ancestry assignment values from PCAdmix....5

S3 Table. Consensus approach results....6

S4 Table. Results of the GO term enrichment analysis.....7

S1 Figure. Admixture plots....18

S2 Figure. f3/f4 score distribution and outlier selection....19

S3 Figure. Schematic outline of the 'Consistently Introgressed Windows of Interest (CIWI)' approach.....20

S4 Figure. PCA plot....21

S5 Figure. Neighbour-Net plot.....22

S6 Figure. Treemix plots....23

S7 Figure. Graphical representation of PCAdmix results....24

S8 Figure. Consistently Introgressed Windows of Interest (CIWI).....39

S1 Text. Supplementary materials and methods.

Sample information

The samples denoted MSar1 were collected in the mountainous Central-Eastern part of Sardinia belonging to the "Ogliastra" and "Supramonte" toponyms partially enclosed in the Gennargentu National park. This area has been historically sparsely settled, featuring harsh terrain, deep ravines and cold winters. Blood (preserved in EDTA) or tissue samples (preserved in ethanol) from poached animals found by the forestry department in this area were collected and stored at -20°C. Sampling for MSar2, MCor and MCyp was carried out between 2005 and 2012. Peripheral blood was collected from captured animals and stored in EDTA vacutainers. The MSar2 and MCor samples came from animals captured in the Montes area, a protected area within the Gennargentu national park, and the Cinto massif (North-Central Corsica) respectively. The MCyp samples were obtained from captured specimens belonging to mouflon living in the Paphos forest (Cyprus). Data from a third group of Sardinian mouflon subpopulation (MSar3) and a mouflon population from Spain (MSpa) were available from the Sheep HapMap project[1]. Two blood samples of Iranian mouflon (*Ovis orientalis gmelinii*) from North-West Iran were also genotyped.

Ten Sarda sheep individuals (SAR) were sampled in the same area overlapping with MSar1 population. The Sarda sheep is an autochthonous breed comprising the vast majority of the Sardinian sheep population and is almost exclusively bred for milk production. Eight Hungarian Racka sheep (RAK) were also sampled, as Racka is an autochthonous sheep from Hungary [2] used in the past to improve trophy size in mouflon used to repopulate the Caucasus[3].

Apart from SAR individuals all the domestic sheep data were available from the Sheep HapMap project[1]. Among these are 20 individuals of Pecora Nera di Arbus (SAB), the only other autochthonous Sardinian domestic sheep (this breed presents a mixture of modern and ancestral traits e.g., non-shedding and black fleece[4]), and breeds from the first wave of domestication [5] such as Soay from the island of St. Kilda (UK) and Spael from Norway.

Population structure analyses

Admixture

Two additional Admixture analyses (K = 2 to 5) were performed after removal of the most inbred and divergent sheep populations: 1) SOA, CHI and CFT, and 2) SOA, CHI, CFT, VBN, SBF and SPW (Supplementary Fig. S1) to evaluate the possible distortion of mouflon global ancestry inference due to the presence of highly inbred and/or divergent populations in the dataset. Four replicates of a supervised Admixture analysis were also performed using MHun, MSar2, and each of the four domestic reference population used for the local ancestry analyses as starting ancestral populations for the supervised algorithm (Supplementary Fig. S1).

f3/f4 tests

The f3 test investigates a target population *A* as result of admixture between two source populations (*B*, *C*). For each *A*:*B*,*C* combination an f3 score is produced, which if significantly negative is indicative of admixture in *A*. The f4 test investigates the tree topology of four populations ((*A*,*B*),(*C*,*D*)) and generates an f4 score which if deviating from zero indicates more gene flow (admixture) between a certain pair of the quartet than between the other pairs. Three tree topologies are possible for each four populations combination, i.e. ((*A*,*B*),(*C*,*D*)), ((*A*,*C*),(*B*,*D*)), and ((*A*,*D*),(*B*,*C*)). Here, we investigated all the 5,313 three

populations combinations generated by the f3 test, and the 31,878 possible tree topologies generated by four population combinations out of 24 populations.

In order to identify 'extreme' f3 and f4 scores, scores were normalised using the 'MLE_LambertW' function within the R package 'LambertW'[6]. The normalising function was applied with default parameters, except the 'starting distribution type' which was set to 'skewed' and 'heavy tails' for the f3 and f4 dataset, respectively (see Supplementary Figure S1). The R function 'Boxplot' was used to identify outliers as those values lying outside 1.5 times the interquartile range above the upper quartile and below the lower quartile. (Supplementary Figure S2).

PCAdmix parameters.
When a linkage map is not available, the window size used by PCAdmix is defined by a fixed number of SNPs, otherwise different approaches are possible by using physical and genetic distances. Also, LD pruning is encouraged by the authors to "prevent high-LD blocks from having excessive influence on the inferred ancestry of a region, while retaining a dense, informative set of SNPs"[7]. For our dataset a linkage map was not available, therefore a SNP based window size definition was used. The default value for window size in PCAdmix is set to 20 SNPs and refers to high-density datasets (1 SNP every ~5kbp). Given the lower density of the Ovine SNP chip (1 SNP every ~40kbp) we applied 5 as SNPs/window parameter as a lower number of SNPs/window is best suited for sparser datasets [7].

We used MSar2 as representatives of the Sardinian mouflon as it showed no sign of either domestic sheep introgression or mixed cluster components according to Admixture analysis (Fig 2). For the Corsican mouflon, as MCor and MHun showed highly similar signals of ancestry in all 24 clustering solutions produced by Admixture, we used the more extensively sampled MHun. The domestic breeds selected as reference for this analysis were: SAB, a sympatric breed with both SAR and Sardinian mouflon populations, CAS and LAC, two European breeds from Spain and southern France respectively, areas where mouflon are still present: southern France was one of the first area where mouflon were reintroduced at the end of the 18th century. We also included ASM that has been widely used to improve several European breeds [1,8,9]. These breeds were chosen as they live in the same areas where mouflon are present and in order to provide different ancestry assignment for each combination. These reference populations were used to infer local ancestry in the following sheep populations breeds: ALT, CHI, CHU, COM, CFT, RAK, SAR, SBF, SOA, SPW, VBN and the feral MSar1, MSar3 and MSpa populations (populations with less than eight individuals were excluded). A similar analysis was performed using MSar1_p as reference for Sardinian mouflon. When using the SAB and MSar1 samples as reference populations, we removed individuals that showed evidence of introgression (introgressed ancestry component >0.01) based on the Admixture results (see below). These subsampled populations were renamed SAB_p and MSar1_p with the "p" denoting "pure".

Consensus approach
The limitation given by applying a chromosome painting method as PCAdmix to such a sparse dataset resulted in high noise in analyses where only two populations were compared. Despite of the large number of markers in the SNP chip (~54k), the SNPs in this array are distanced from each other on average by ~60k base pairs leaving large gaps that may harbour additional and valuable information. Additionally, SNP array data are not in phase by definition, implying that the correct coupling of alleles needs to be inferred statistically in order to have haplotypes for multiple types of analyses like the ones implemented here. The accuracy of ancestry assignment depends on the correctness of the inferred haplotypes[7], and

it has been shown that it is easier to achieve with denser SNP panels [10]. Therefore, as our data is based on the medium-density OvineSNP50 BeadChip, we developed a consensus approach that uses several domestic breeds as reference populations and minimizes the effect of spurious inferences from incorrect haplotype imputation.

For one or more reference populations (see main text) an assignment score (A-Score) was calculated for each window as defined by PCAdmix. Such score was calculated averaging the posterior probability (PP) assigned to the reference population of interest by root mean square, hence reducing the weight of low PP values in the final score. As using different reference populations resulted in the software defining windows with different sizes and genomic locations, the arrays of A-Scores from different analyses could not be compared directly because the windows would not perfectly overlap. A sliding window method was therefore implemented to normalize the window sizes among different sets of A-Scores by transferring the A-Score information into a window frame common to all of the reference populations. The A-Score information was transferred to a new set of windows of equal size common for all the reference populations. The size of the windows was defined as half the size of the smallest window defined by PCAdmix among the data under analysis. Subsequently, we defined a window concordance score as the harmonic mean of the A-scores within the window. We repeated this analysis for each chromosome, enabling us to define genomic regions that exhibit concordance scores outside the 95th and 99th percentiles of the genome-wide concordance scores and defined in the main text as Constantly Introgressed Windows of Interest (CIWI) (see Supplementary Fig S3).

Bibliography

1. Kijas, J. W. *et al.* Genome-Wide Analysis of the World's Sheep Breeds Reveals High Levels of Historic Mixture and Strong Recent Selection. *PLoS Biol.* **10,** e1001258 (2012).
2. Ryder, M. A survey of European primitive breeds of sheep. *Ann. Genet. Sel. anim* **13,** 381–418 (1981).
3. Tomiczek, H. & Türcke, F. *Das Muffelwild.* (2003).
4. Piras, M., Casu, S., Salaris, S., Usai, M. G. & Carta, A. The Pecora Nera di Arbus: a new sheep breed in Sardinia, Italy. *Anim. Genet. Resour. Inf.* **45,** 91 (2009).
5. Chessa, B. *et al.* Revealing the history of sheep domestication using retrovirus integrations. *Science* **324,** 532–6 (2009).
6. Goerg, G. M. LambertW: An R package for Lambert W x F Random Variables. (2016).
7. Brisbin, A. *et al.* PCAdmix: principal components-based assignment of ancestry along each chromosome in individuals with admixed ancestry from two or more populations. *Hum. Biol.* **84,** 343–64 (2012).
8. Bon, R. *et al.* Le Mouflon de Corse. *Reveud Ecol.* 67–110 (1991).
9. Ciani, E. *et al.* Merino and Merino-derived sheep breeds: a genome-wide intercontinental study. *Genet. Sel. Evol.* **47,** 64 (2015).
10. Hayes, B. J., Bowman, P. J., Daetwyler, H. D., Kijas, J. W. & van der Werf, J. H. J. Accuracy of genotype imputation in sheep breeds. *Anim. Genet.* **43,** 72–80 (2012).

S1 Table. f3 tests most significant results.

Extreme f3 scores suggest *A* being the result of admixture of *B* and *C*.

A	*B*	*C*	*score*
MSar3	MSar1	SAR	*-25.434*
MSar3	MSar1	SAB	*-22.1235*
MSar3	MSar1	IRS	*-19.6942*
MSar3	MSar1	COM	*-18.6248*
MSar3	MSar1	ALT	*-17.5988*
MSar3	MSar1	CFT	*-17.0330*
MSar3	MSar1	CHI	*-16.0818*
MSar3	MSar1	CAS	*-15.9664*
MSar3	MSar1	RAK	*-15.7839*
MSar3	MSar1	LAC	*-15.3357*
MSar3	MSar1	ASM	-14.5606
MSar3	MSar1	CHU	-14.3898

S2 Table. Genome-wide local ancestry assignment values from PCAdmix.

The genome-wide ancestry assignment inferred by PCAdmix (in %), based on comparison of focal individuals with three reference populations (Sardinian mouflon (MSar2), Corsican mouflon (MHun) and a domestic sheep breed. This was replicated in total four times, each using a different domestic breed (CAS, ASM, LAC or SAB_p). The percentage of the genome assigned with posterior probability >0.95 is displayed for each analysed reference populations, along with the corresponding cumulative percentage of regions assigned below the 0.95 probability threshold. For population abbreviations see Table 1.

Reference populations

	MSar2	*MHun*	*CAS*	*<95*	*MSar2*	*MHun*	*ASM*	*<95*	*MSar2*	*MHun*	*LAC*	*<95*	*MSar2*	*MHun*	*SAB_p*	*<95*
Mouflon																
MSar1	**26.6**	**18.0**	12.7	42.7	**25.7**	**18.6**	11.3	44.4	**26.4**	**19.7**	10.7	43.2	**26.3**	**25.1**	8.5	40.0
MSar3	***15.9***	***10.1***	*33.3*	*40.7*	***16.1***	***10.2***	*32.4*	*41.2*	***16.8***	***10.8***	*31.1*	*41.3*	***17.2***	***14.8***	*25.5*	*42.5*
MSpa	**19.0**	**43.6**	4.2	33.3	**18.2**	**44.8**	4.2	32.9	**16.0**	**49.2**	3.6	31.2	**14.1**	**55.7**	3.9	26.3
Sheep																
ALT	0.9	0.8	**90.1**	8.2	1.0	1.1	**88.9**	9.0	1.4	1.8	**84.7**	12.1	2.8	6.6	**65.5**	25.2
CHI	0.6	0.4	**93.9**	5.0	0.8	0.5	**92.9**	5.9	1.0	1.4	**89.1**	8.5	3.1	6.4	**67.4**	23.1
CHU	0.9	1.0	**91.5**	6.7	1.1	1.0	**88.5**	9.4	1.6	1.8	**84.7**	11.9	3.1	7.3	**63.5**	26.1
COM	0.9	0.9	**90.5**	7.7	1.6	1.0	**88.3**	9.2	1.4	1.7	**85.0**	11.9	3.0	6.5	**65.5**	25.0
CTF	0.6	0.4	**94.7**	4.4	0.6	0.6	**93.4**	5.4	0.7	1.1	**91.5**	6.7	2.1	7.0	**71.2**	19.7
RAK	0.8	0.6	**93.3**	5.4	1.3	0.8	**91.2**	6.7	1.1	1.3	**90.2**	7.5	2.2	6.5	**70.2**	21.1
SAR	1.1	0.7	**91.2**	7.0	1.0	0.9	**90.2**	8.0	1.4	1.5	**87.0**	10.1	2.4	5.4	**73.4**	18.7
SBF	1.1	0.9	**89.5**	8.5	1.1	1.3	**88.1**	9.4	1.3	2.4	**83.9**	12.5	2.8	8.7	**61.9**	26.6
SOA	1.8	1.8	**88.0**	8.5	2.1	2.5	**84.6**	10.9	2.3	3.1	**82.3**	12.4	3.2	9.1	**65.0**	22.7
SPW	1.0	1.0	**89.4**	8.6	1.3	1.2	**87.8**	9.7	1.2	2.1	**84.8**	12.0	3.2	8.6	**62.1**	26.0
VBN	0.8	0.7	**92.0**	6.4	1.1	0.9	**90.9**	7.1	1.2	1.5	**88.4**	8.9	2.8	7.4	**66.5**	23.4

S3 Table. Consensus approach results.

Number of Consistently Introgressed Windows of Interest (CIWIs) and genes associated to those windows identified applying the consensus approach described in this work. Two genome-wide consensus score thresholds at the 99th and 95th percentile were used. For population abbreviations see Table 1.

Breed	***CIWI***	***genes***	***CIWI***	***Genes***
	95th percentile		*99th percentile*	
mouflon				
MSar1	1784	641	347	167
MSar3	1740	488	344	57
MSpa	2609	443	501	125
domestic sheep				
ALT	1264	796	252	164
CHI	2678	993	497	183
CHU	5346	1077	1003	299
COM	5057	1722	1014	466
CFT	6170	1225	1199	390
RAK	1823	451	356	74
SAR	1499	769	291	232
SBF	5310	1168	1037	366
SOA	1794	926	359	94
SPW	1776	781	355	222
VBN	2543	1051	507	297

S4 Table. Results of the GO term enrichment analysis.

The two sets of genes identified through the CIWI approach (top 1st and 5th percentile used as threshold) were screened for GO term enrichment. The population acronym, percentile threshold used, GO term identifier, description of the GO term, uncorrected *P-value*, and false discovery rate (FDR) corrected q-values are shown. GO terms with FDR < 0.05 are displayed in bold. A) GO terms identified and discussed in the main text. B) GO terms identified when a similar CIWI analysis was performed comparing eight ancestry assignment analyses results.

For population abbreviations see Table 1.

A)

Population	***percentile threshold***	***GO term identifier.***	***Description***	***P-value***	***FDR q-value***
MSar1	*99*	-	*none*	-	-
	95	GO:0042157	*lipoprotein metabolic process*	5.36E-04	1.00E+00
		GO:0010885	*regulation of cholesterol storage*	6.10E-04	1.00E+00
		GO:2000177	*regulation of neural precursor cell proliferation*	7.59E-04	1.00E+00
		GO:2000179	*positive regulation of neural precursor cell proliferation*	8.89E-04	1.00E+00
MSar3	*99*	GO:0019935	cyclic-nucleotide-mediated signalling	1.08E-04	1.00E+00
		GO:0019933	cAMP-mediated signalling	2.82E-04	1.00E+00
		GO:0001654	eye development	7.64E-04	1.00E+00
		GO:0048598	embryonic morphogenesis	8.14E-04	1.00E+00
	95	GO:0051972	regulation of telomerase activity	4.49E-04	1.00E+00
		GO:0045216	cell-cell junction organization	6.03E-04	1.00E+00
MSpa	*99*	GO:0050819	negative regulation of coagulation	9.03E-05	1.00E+00
		GO:0050818	regulation of coagulation	1.29E-04	7.75E-01
		GO:0010543	regulation of platelet activation	3.39E-04	1.00E+00
	95	GO:2000009	negative regulation of protein localization to cell surface	6.65E-04	1.00E+00
SAR	*99*	GO:0018101	protein citrullination	9.39E-06	1.11E-01
		GO:0050912	detection of chemical stimulus involved in sensory perception of taste	2.02E-05	1.20E-01
		GO:0019240	citrulline biosynthetic process	7.98E-05	3.14E-01
		GO:0001580	detection of chemical stimulus involved in sensory perception of bitter taste	1.26E-04	3.73E-01
		GO:0036413	histone H3-R26 citrullination	1.79E-04	4.24E-01
		GO:0036414	histone citrullination	1.79E-04	3.53E-01
		GO:2001199	negative regulation of dendritic cell differentiation	1.79E-04	3.03E-01
		GO:0018195	peptidyl-arginine modification	2.66E-04	3.92E-01
		GO:0000052	citrulline metabolic process	2.66E-04	3.49E-01
		GO:0045909	positive regulation of vasodilation	4.77E-04	5.64E-01
		GO:0042398	cellular modified amino acid biosynthetic process	4.96E-04	5.33E-01
		GO:2001198	regulation of dendritic cell differentiation	5.33E-04	5.25E-01
		GO:0007028	cytoplasm organization	5.33E-04	4.85E-01

	95	**GO:0018101**	**protein citrullination**	**3.99E-07**	**4.72E-03**
		**GO:0019240**	**citrulline biosynthetic process**	**5.32E-07**	**3.15E-03**
		**GO:0001580**	**detection of chemical stimulus involved in sensory perception of bitter taste**	**6.81E-07**	**2.69E-03**
		**GO:0050912**	**detection of chemical stimulus involved in sensory perception of taste**	**8.97E-07**	**2.65E-03**
		**GO:0000052**	**citrulline metabolic process**	**7.66E-06**	**1.81E-02**
		**GO:0042398**	**cellular modified amino acid biosynthetic process**	**2.07E-05**	**4.09E-02**
		GO:0018195	peptidyl-arginine modification	1.41E-04	2.39E-01
		GO:0006997	nucleus organization	5.60E-04	8.28E-01
		GO:1901607	alpha-amino acid biosynthetic process	8.13E-04	1.00E+00
ALT	*99*		none		
	95	GO:0019240	citrulline biosynthetic process	6.46E-4	1E0
CHI	*99*	GO:0000956	nuclear-transcribed mRNA catabolic process	4.18E-4	1E0
		GO:0006402	mRNA catabolic process	7.06E-4	1E0
		GO:0019058	viral life cycle	8.92E-4	1E0
		GO:0060669	embryonic placenta morphogenesis	9.18E-4	1E0
	95	**GO:0018101**	**protein citrullination**	**2.18E-6**	**2.64E-2**
		GO:0019240	citrulline biosynthetic process	1.01E-4	6.13E-1
		GO:0071442	positive regulation of histone H3-K14 acetylation	4.03E-4	1E0
		GO:0018195	peptidyl-arginine modification	6.91E-4	1E0
		GO:0000052	citrulline metabolic process	6.91E-4	1E0
CHU	*99*	**GO:0019240**	**citrulline biosynthetic process**	**2.04E-9**	**2.46E-5**
		**GO:0018101**	**protein citrullination**	**3.72E-9**	**2.24E-5**
		**GO:0000052**	**citrulline metabolic process**	**3.2E-8**	**1.28E-4**
		**GO:0018195**	**peptidyl-arginine modification**	**1.55E-6**	**4.67E-3**
		GO:0042398	cellular modified amino acid biosynthetic process	2.41E-5	5.79E-2
		GO:1901607	alpha-amino acid biosynthetic process	1.61E-4	3.22E-1
		GO:0008652	cellular amino acid biosynthetic process	3.98E-4	6.85E-1
		GO:0036413	histone H3-R26 citrullination	4.32E-4	6.49E-1
		GO:0036414	histone citrullination	4.32E-4	5.77E-1
	95	**GO:0018101**	**protein citrullination**	**2.23E-6**	**2.69E-2**
		**GO:0019240**	**citrulline biosynthetic process**	**4.04E-6**	**2.44E-2**
		GO:0000052	citrulline metabolic process	5.48E-5	2.21E-1
		GO:0018195	peptidyl-arginine modification	7.04E-4	1E0
		GO:0018130	heterocycle biosynthetic process	9.78E-4	1E0
COM	*99*	**GO:0018101**	**protein citrullination**	**4.92E-08**	**5.84E-04**
		**GO:0019240**	**citrulline biosynthetic process**	**2.53E-06**	**1.50E-02**
		**GO:0018195**	**peptidyl-arginine modification**	**1.91E-05**	**7.56E-02**
		GO:0000052	citrulline metabolic process	1.91E-05	5.67E-02
		GO:0006824	cobalt ion transport	3.95E-04	9.37E-01

		GO:0015889	cobalamin transport	3.95E-04	7.81E-01
	95	GO:0018101	protein citrullination	3.28E-05	3.93E-01
		GO:0021756	striatum development	5.51E-04	1.00E+00
		GO:0035637	multicellular organismal signalling	6.97E-04	1.00E+00
VBN	*99*	**GO:0018101**	**protein citrullination**	**1.80E-09**	**2.13E-05**
		**GO:0019240**	**citrulline biosynthetic process**	**9.63E-08**	**5.72E-04**
		**GO:0018195**	**peptidyl-arginine modification**	**7.60E-07**	**3.01E-03**
		**GO:0000052**	**citrulline metabolic process**	**7.60E-07**	**2.26E-03**
		GO:0042398	cellular modified amino acid biosynthetic process	1.50E-04	3.57E-01
		GO:0036413	histone H3-R26 citrullination	3.23E-04	6.40E-01
		GO:0036414	histone citrullination	3.23E-04	5.48E-01
		GO:0060448	dichotomous subdivision of terminal units involved in lung branching	3.23E-04	4.80E-01
		GO:1901607	alpha-amino acid biosynthetic process	5.57E-04	7.35E-01
		GO:0008652	cellular amino acid biosynthetic process	8.91E-04	1.00E+00
		GO:0044406	adhesion of symbiont to host	9.59E-04	1.00E+00
	95	**GO:0018101**	**protein citrullination**	**1.96E-06**	**2.34E-02**
		GO:0000052	citrulline metabolic process	8.91E-05	5.31E-01
		GO:0019240	citrulline biosynthetic process	9.12E-05	3.63E-01
		GO:0071322	cellular response to carbohydrate stimulus	2.45E-04	7.30E-01
		GO:0018195	peptidyl-arginine modification	6.26E-04	1.00E+00
RAK	*99*	GO:1902224	ketone body metabolic process	3.27E-04	1.00E+00
		GO:0046950	cellular ketone body metabolic process	3.27E-04	1.00E+00
		GO:2001022	positive regulation of response to DNA damage stimulus	5.89E-04	1.00E+00
	95	**GO:0018101**	**protein citrullination**	**4.08E-08**	**4.85E-04**
		**GO:0019240**	**citrulline biosynthetic process**	**2.10E-06**	**1.25E-02**
		GO:0018195	peptidyl-arginine modification	1.60E-05	6.32E-02
		**GO:0000052**	**citrulline metabolic process**	**1.60E-05**	**4.74E-02**
		GO:0044260	cellular macromolecule metabolic process	2.50E-04	5.95E-01
		GO:0031929	TOR signalling	3.38E-04	6.69E-01
		GO:0044237	cellular metabolic process	3.77E-04	6.40E-01
		GO:0043170	macromolecule metabolic process	5.26E-04	7.81E-01
		GO:0006955	immune response	5.51E-04	7.28E-01
		GO:0007220	Notch receptor processing	6.94E-04	8.25E-01
		GO:0010468	regulation of gene expression	8.50E-04	9.19E-01
CFT	*99*	**GO:0018101**	**protein citrullination**	**1.64E-08**	**1.95E-04**
		**GO:0019240**	**citrulline biosynthetic process**	**8.57E-07**	**5.10E-03**
		**GO:0018195**	**peptidyl-arginine modification**	**6.60E-06**	**2.62E-02**
		**GO:0000052**	**citrulline metabolic process**	**6.60E-06**	**1.96E-02**
		GO:0045109	intermediate filament organization	1.10E-04	2.61E-01
		GO:0071680	response to indole-3-methanol	2.07E-04	4.10E-01
		GO:0071681	cellular response to indole-3-methanol	2.07E-04	3.52E-01

		GO:0006266	DNA ligation	2.07E-04	3.08E-01
		GO:0045103	intermediate filament-based process	3.65E-04	4.82E-01
		GO:0045104	intermediate filament cytoskeleton organization	3.65E-04	4.34E-01
		GO:0086073	bundle of His cell-Purkinje myocyte adhesion involved in cell communication	4.05E-04	4.38E-01
		GO:0014070	response to organic cyclic compound	5.77E-04	5.72E-01
		GO:0097305	response to alcohol	6.08E-04	5.57E-01
		GO:0072364	regulation of cellular ketone metabolic process by regulation of transcription from RNA polymerase II promoter	6.95E-04	5.90E-01
		GO:0086042	cardiac muscle cell-cardiac muscle cell adhesion	6.95E-04	5.51E-01
		GO:0051987	positive regulation of attachment of spindle microtubules to kinetochore	7.78E-04	5.78E-01
		GO:0036413	histone H3-R26 citrullination	7.78E-04	5.44E-01
		GO:0036414	histone citrullination	7.78E-04	5.14E-01
		GO:2001199	negative regulation of dendritic cell differentiation	7.78E-04	4.87E-01
		GO:1901624	negative regulation of lymphocyte chemotaxis	7.78E-04	4.63E-01
	95	GO:0018101	protein citrullination	5.85E-06	6.99E-02
		GO:0070192	chromosome organization involved in meiosis	6.30E-05	3.77E-01
		GO:0018195	peptidyl-arginine modification	1.63E-04	6.48E-01
		GO:0019240	citrulline biosynthetic process	2.60E-04	7.76E-01
		GO:0090280	positive regulation of calcium ion import	2.60E-04	6.21E-01
		GO:0022038	corpus callosum development	3.03E-04	6.03E-01
		GO:0090279	regulation of calcium ion import	4.06E-04	6.93E-01
		GO:0006307	DNA dealkylation involved in DNA repair	8.44E-04	1.00E+00
		GO:0051216	cartilage development	9.32E-04	1.00E+00
SOA	*99*	GO:0010165	response to X-ray	3.62E-04	1.00E+00
		GO:0007185	transmembrane receptor protein tyrosine phosphatase signalling pathway	9.17E-04	1.00E+00
	95	**GO:0001580**	**detection of chemical stimulus involved in sensory perception of bitter taste**	**1.79E-06**	**2.14E-02**
		GO:0050912	detection of chemical stimulus involved in sensory perception of taste	4.58E-05	2.74E-01
		GO:0060193	positive regulation of lipase activity	2.32E-04	9.24E-01
		GO:2000269	regulation of fibroblast apoptotic process	2.82E-04	8.42E-01
		GO:0010518	positive regulation of phospholipase activity	4.61E-04	1.00E+00
		GO:1902106	negative regulation of leukocyte differentiation	7.09E-04	1.00E+00
		GO:1902751	positive regulation of cell cycle G2/M phase transition	7.89E-04	1.00E+00
		GO:0010971	positive regulation of G2/M transition of mitotic cell cycle	7.89E-04	1.00E+00

		GO:0060191	regulation of lipase activity	8.82E-04	1.00E+00
SPW	*99*	GO:0070997	neuron death	8.04E-04	1.00E+00
	95	GO:0036149	phosphatidylinositol acyl-chain remodelling	4.18E-05	4.99E-01
		GO:1902031	regulation of NADP metabolic process	1.45E-04	8.65E-01
		GO:0036152	phosphatidylethanolamine acyl-chain remodelling	5.33E-04	1.00E+00
		GO:0030901	midbrain development	7.65E-04	1.00E+00
		GO:0072329	monocarboxylic acid catabolic process	7.93E-04	1.00E+00
SBF	*99*	**GO:0018101**	**protein citrullination**	**2.38E-06**	**2.82E-02**
		**GO:0000052**	**citrulline metabolic process**	**5.14E-06**	**3.05E-02**
		GO:0019240	citrulline biosynthetic process	3.12E-05	1.24E-01
		GO:0018195	peptidyl-arginine modification	1.38E-04	4.10E-01
		GO:0017038	protein import	2.70E-04	6.41E-01
		GO:0034504	protein localization to nucleus	4.11E-04	8.14E-01
		GO:0003149	membranous septum morphogenesis	7.02E-04	1.00E+00
	95	GO:0018101	protein citrullination	4.20E-06	5.02E-02
		GO:0000052	citrulline metabolic process	1.12E-04	6.70E-01
		GO:0019240	citrulline biosynthetic process	1.90E-04	7.54E-01
		GO:0046599	regulation of centriole replication	7.39E-04	1.00E+00

B)

Population	***percentile threshold***	***GO term identifier.***	***Description***	***P-value***	***FDR q-value***
MSar1	*99*	-	*none*	-	-
	95	GO:0042157	*lipoprotein metabolic process*	5.36E-04	1.00E+00
		GO:0010885	*regulation of cholesterol storage*	6.10E-04	1.00E+00
		GO:2000177	*regulation of neural precursor cell proliferation*	7.59E-04	1.00E+00
		GO:2000179	*positive regulation of neural precursor cell proliferation*	8.89E-04	1.00E+00
MSar2	*99*	-	*none*	-	-
	95	-	*none*	-	-
MSar3	*99*	-	*none*	-	-
	95	GO:0032845	*negative regulation of homeostatic process*	5.5E-4	1E0
MSpa	*99*	GO:0035176	*social behaviour*	1.54E-04	1.00E+00
		GO:0051703	*intraspecies interaction between organisms*	1.54E-04	9.53E-01
		GO:0070942	*neutrophil mediated cytotoxicity*	2.89E-04	1.00E+00
		GO:0070943	*neutrophil mediated killing of symbiont cell*	2.89E-04	8.97E-01
		GO:0051873	*killing by host of symbiont cells*	5.75E-04	1.00E+00
		GO:0051852	*disruption by host of symbiont cells*	5.75E-04	1.00E+00
		GO:0006686	*sphingomyelin biosynthetic process*	5.75E-04	1.00E+00
		GO:0050765	*negative regulation of phagocytosis*	5.75E-04	8.91E-01
		GO:0050764	*regulation of phagocytosis*	6.89E-04	9.49E-01
		GO:0051705	*multi-organism behaviour*	9.03E-04	1.00E+00

		GO:0045073	*regulation of chemokine biosynthetic process*	9.52E-04	1.00E+00
		GO:0048569	*post-embryonic organ development*	9.52E-04	9.84E-01
	95	GO:0010738	*regulation of protein kinase A signalling*	7.26E-05	9.04E-01
		GO:2000977	*regulation of forebrain neuron differentiation*	9.56E-05	5.96E-01
		GO:0051961	*negative regulation of nervous system development*	4.56E-04	1.00E+00
		GO:0042363	*fat-soluble vitamin catabolic process*	8.92E-04	1.00E+00
		GO:0048569	*post-embryonic organ development*	8.92E-04	1.00E+00
		GO:0031642	*negative regulation of myelination*	8.92E-04	1.00E+00
ALT	*99*	**GO:0018101**	***protein citrullination***	**2.99E-06**	**3.71E-02**
		GO:0019240	*citrulline biosynthetic process*	1.65E-05	1.02E-01
		GO:0000052	*citrulline metabolic process*	3.51E-05	1.45E-01
		GO:0018195	*peptidyl-arginine modification*	6.37E-05	1.97E-01
		GO:0090309	*positive regulation of methylation-dependent chromatin silencing*	2.72E-04	6.75E-01
		GO:0099643	*signal release from synapse*	5.92E-04	1.00E+00
		GO:0031937	*positive regulation of chromatin silencing*	6.75E-04	1.00E+00
	95	**GO:0018101**	***protein citrullination***	**5.76E-07**	**7.18E-03**
		GO:0019240	*citrulline biosynthetic process*	2.79E-05	1.74E-01
		GO:0000052	*citrulline metabolic process*	1.14E-04	4.75E-01
		GO:0018195	*peptidyl-arginine modification*	3.26E-04	1.00E+00
		GO:0010324	*membrane invagination*	4.64E-04	1.00E+00
		GO:0090309	*positive regulation of methylation-dependent chromatin silencing*	6.95E-04	1.00E+00
		GO:0031935	*regulation of chromatin silencing*	7.50E-04	1.00E+00
CHI	*99*	GO:0010224	*response to UV-B*	4.11E-05	5.10E-01
		GO:0071493	*cellular response to UV-B*	1.93E-04	1.00E+00
		GO:0071214	*cellular response to abiotic stimulus*	4.66E-04	1.00E+00
		GO:0039663	*membrane fusion involved in viral entry into host cell*	4.70E-04	1.00E+00
		GO:0019064	*fusion of virus membrane with host plasma membrane*	4.70E-04	1.00E+00
		GO:1901628	*positive regulation of postsynaptic membrane organization*	4.70E-04	9.73E-01
		GO:0044800	*multi-organism membrane fusion*	4.70E-04	8.34E-01
		GO:0071478	*cellular response to radiation*	6.29E-04	9.77E-01
		GO:0030212	*hyaluronan metabolic process*	9.71E-04	1.00E+00
	95	GO:0018101	*protein citrullination*	4.61E-06	5.76E-02
		GO:0019240	*citrulline biosynthetic process*	2.07E-04	1.00E+00
		GO:0042398	*cellular modified amino acid biosynthetic process*	2.31E-04	9.61E-01
		GO:1901628	*positive regulation of postsynaptic membrane organization*	6.31E-04	1.00E+00
		GO:0000052	*citrulline metabolic process*	8.05E-04	1.00E+00
CHU	*99*	**GO:0019240**	***citrulline biosynthetic process***	**2.09E-09**	**2.60E-05**
		**GO:0018101**	***protein citrullination***	**3.80E-09**	**2.36E-05**

		**GO:0000052**	***citrulline metabolic process***	**1.52E-08**	**6.27E-05**
		**GO:0018195**	***peptidyl-arginine modification***	**2.67E-06**	**8.28E-03**
		GO:0042398	*cellular modified amino acid biosynthetic process*	3.02E-05	7.50E-02
		GO:1901607	*alpha-amino acid biosynthetic process*	1.42E-04	2.94E-01
		GO:0036413	*histone H3-R26 citrullination*	4.35E-04	7.72E-01
		GO:0036414	*histone citrullination*	4.35E-04	6.75E-01
		GO:0008652	*cellular amino acid biosynthetic process*	4.57E-04	6.30E-01
		GO:0006638	*neutral lipid metabolic process*	6.37E-04	7.90E-01
	95	**GO:0018101**	***protein citrullination***	**2.23E-06**	**2.78E-02**
		**GO:0019240**	***citrulline biosynthetic process***	**4.04E-06**	**2.52E-02**
		GO:0000052	*citrulline metabolic process*	2.66E-05	1.11E-01
		GO:0007265	*Ras protein signal transduction*	6.68E-04	1.00E+00
COM	*99*	**GO:0018101**	***protein citrullination***	**9.19E-09**	**1.14E-04**
		**GO:0019240**	***citrulline biosynthetic process***	**4.84E-07**	**3.01E-03**
		**GO:0000052**	***citrulline metabolic process***	**2.09E-06**	**8.66E-03**
		**GO:0018195**	***peptidyl-arginine modification***	**6.30E-06**	**1.96E-02**
		GO:0032423	*regulation of mismatch repair*	6.18E-04	1.00E+00
		GO:1900114	*positive regulation of histone H3-K9 trimethylation*	6.18E-04	1.00E+00
		GO:0036413	*histone H3-R26 citrullination*	6.18E-04	1.00E+00
		GO:0036414	*histone citrullination*	6.18E-04	9.60E-01
		GO:0097094	*craniofacial suture morphogenesis*	7.79E-04	1.00E+00
		GO:0042398	*cellular modified amino acid biosynthetic process*	7.86E-04	9.77E-01
		GO:0010324	*membrane invagination*	8.72E-04	9.85E-01
	95	**GO:0018101**	***protein citrullination***	**1.83E-06**	**2.28E-02**
		GO:0019240	*citrulline biosynthetic process*	8.52E-05	5.32E-01
		GO:1903310	*positive regulation of chromatin modification*	2.79E-04	1.00E+00
		GO:0000052	*citrulline metabolic process*	3.40E-04	1.00E+00
		GO:0045343	*regulation of MHC class I biosynthetic process*	3.62E-04	9.05E-01
		GO:0046532	*regulation of photoreceptor cell differentiation*	3.62E-04	7.54E-01
		GO:0031058	*positive regulation of histone modification*	5.37E-04	9.59E-01
		GO:0045216	*cell-cell junction organization*	6.51E-04	1.00E+00
		GO:0019083	*viral transcription*	7.11E-04	9.86E-01
		GO:0043491	*protein kinase B signalling*	8.09E-04	1.00E+00
		GO:0018195	*peptidyl-arginine modification*	9.47E-04	1.00E+00
CTF	*99*	**GO:0018101**	***protein citrullination***	**3.85E-06**	**4.77E-02**
		GO:0019240	*citrulline biosynthetic process*	3.29E-05	2.04E-01
		GO:0000052	*citrulline metabolic process*	7.79E-05	3.22E-01
		GO:0018195	*peptidyl-arginine modification*	1.51E-04	4.67E-01

		GO:0090309	*positive regulation of methylation-dependent chromatin silencing*	5.86E-04	1.00E+00
	95	GO:0050907	*detection of chemical stimulus involved in sensory perception*	1.12E-04	1.00E+00
		GO:0050911	*detection of chemical stimulus involved in sensory perception of smell*	1.34E-04	8.35E-01
		GO:0009593	*detection of chemical stimulus*	2.42E-04	1.00E+00
		GO:0018101	*protein citrullination*	4.96E-04	1.00E+00
		GO:0048265	*response to pain*	6.66E-04	1.00E+00
RAK	*99*	**GO:0018101**	***protein citrullination***	**1.41E-09**	**1.76E-05**
		**GO:0019240**	***citrulline biosynthetic process***	**7.59E-08**	**4.72E-04**
		**GO:0000052**	***citrulline metabolic process***	**3.32E-07**	**1.38E-03**
		**GO:0018195**	***peptidyl-arginine modification***	**1.02E-06**	**3.15E-03**
		GO:0042398	*cellular modified amino acid biosynthetic process*	1.42E-04	3.53E-01
		GO:0051987	*positive regulation of attachment of spindle microtubules to kinetochore*	2.94E-04	6.09E-01
		GO:0036413	*histone H3-R26 citrullination*	2.94E-04	5.22E-01
		GO:0036414	*histone citrullination*	2.94E-04	4.57E-01
		GO:1901607	*alpha-amino acid biosynthetic process*	5.07E-04	7.00E-01
		GO:0051171	*regulation of nitrogen compound metabolic process*	6.62E-04	8.22E-01
		GO:0019219	*regulation of nucleobase-containing compound metabolic process*	7.39E-04	8.35E-01
	95	GO:0018101	*protein citrullination*	4.74E-06	5.92E-02
		GO:0019240	*citrulline biosynthetic process*	2.12E-04	1.00E+00
		GO:0045343	*regulation of MHC class I biosynthetic process*	2.57E-04	1.00E+00
		GO:0010721	*negative regulation of cell development*	6.64E-04	1.00E+00
		GO:0048096	*chromatin-mediated maintenance of transcription*	7.17E-04	1.00E+00
		GO:0000052	*citrulline metabolic process*	8.25E-04	1.00E+00
SAR	*99*	GO:0006469	*negative regulation of protein kinase activity*	3.80E-05	4.71E-01
		GO:0033673	*negative regulation of kinase activity*	4.94E-05	3.06E-01
		GO:0001933	*negative regulation of protein phosphorylation*	1.41E-04	5.83E-01
		GO:0042326	*negative regulation of phosphorylation*	2.46E-04	7.63E-01
		GO:0051348	*negative regulation of transferase activity*	2.74E-04	6.79E-01
		GO:0002710	*negative regulation of T cell mediated immunity*	5.32E-04	1.00E+00
		GO:0014741	*negative regulation of muscle hypertrophy*	5.32E-04	9.42E-01
		GO:0021819	*layer formation in cerebral cortex*	7.42E-04	1.00E+00
	95	**GO:0018101**	***protein citrullination***	**1.37E-08**	**1.70E-04**

		**GO:0019240**	***citrulline biosynthetic process***	**7.17E-07**	**4.45E-03**
		**GO:0000052**	***citrulline metabolic process***	**3.09E-06**	**1.28E-02**
		**GO:0018195**	***peptidyl-arginine modification***	**9.28E-06**	**2.88E-02**
		GO:1901607	*alpha-amino acid biosynthetic process*	8.29E-05	2.06E-01
		GO:0008652	*cellular amino acid biosynthetic process*	3.11E-04	6.43E-01
		GO:0060416	*response to growth hormone*	6.25E-04	1.00E+00
		GO:0060544	*regulation of necroptotic process*	6.25E-04	9.71E-01
		GO:0036413	*histone H3-R26 citrullination*	7.24E-04	9.99E-01
		GO:0036414	*histone citrullination*	7.24E-04	8.99E-01
SBF	*99*	GO:0018101	*protein citrullination*	2.98E-05	3.69E-01
		GO:0019240	*citrulline biosynthetic process*	2.49E-04	1.00E+00
		GO:0036413	*histone H3-R26 citrullination*	3.87E-04	1.00E+00
		GO:0036414	*histone citrullination*	3.87E-04	1.00E+00
		GO:0000052	*citrulline metabolic process*	5.81E-04	1.00E+00
		GO:0072009	*nephron epithelium development*	8.18E-04	1.00E+00
	95	GO:0045815	*positive regulation of gene expression, epigenetic*	1.77E-04	1.00E+00
		GO:0046677	*response to antibiotic*	2.69E-04	1.00E+00
		GO:0007263	*nitric oxide mediated signal transduction*	5.63E-04	1.00E+00
		GO:0034612	*response to tumour necrosis factor*	8.53E-04	1.00E+00
SOA	*99*	GO:0018101	*protein citrullination*	9.53E-06	1.18E-01
		GO:0019240	*citrulline biosynthetic process*	8.09E-05	5.02E-01
		GO:0036413	*histone H3-R26 citrullination*	1.81E-04	7.49E-01
		GO:0036414	*histone citrullination*	1.81E-04	5.62E-01
		GO:0000052	*citrulline metabolic process*	1.90E-04	4.73E-01
		GO:0018195	*peptidyl-arginine modification*	3.67E-04	7.59E-01
		GO:0019226	*transmission of nerve impulse*	6.23E-04	1.00E+00
	95	GO:0002823	*negative regulation of adaptive immune response based on somatic recombination of immune receptors built from immunoglobulin superfamily domains*	7.95E-05	9.91E-01
		GO:0002820	*negative regulation of adaptive immune response*	2.82E-04	1.00E+00
		GO:0045620	*negative regulation of lymphocyte differentiation*	5.92E-04	1.00E+00
		GO:0006643	*membrane lipid metabolic process*	6.81E-04	1.00E+00
		GO:0046467	*membrane lipid biosynthetic process*	7.35E-04	1.00E+00
		GO:0018101	*protein citrullination*	7.69E-04	1.00E+00
		GO:0034244	*negative regulation of transcription elongation from RNA polymerase II promoter*	7.69E-04	1.00E+00
SPW	*99*	-	*none*	-	-
	95	**GO:0018101**	***protein citrullination***	**4.85E-07**	**6.05E-03**

		**GO:0019240**	***citrulline biosynthetic process***	**6.70E-07**	**4.18E-03**
		**GO:0000052**	***citrulline metabolic process***	**4.57E-06**	**1.90E-02**
		GO:0050904	*diapedesis*	1.64E-04	5.10E-01
		GO:0002698	*negative regulation of immune effector process*	1.84E-04	4.59E-01
		GO:0018195	*peptidyl-arginine modification*	2.78E-04	5.78E-01
		GO:0042398	*cellular modified amino acid biosynthetic process*	9.61E-04	1.00E+00
VBN	*99*	GO:0018101	*protein citrullination*	7.37E-06	9.15E-02
		GO:0019240	*citrulline biosynthetic process*	6.27E-05	3.90E-01
		GO:0000052	*citrulline metabolic process*	1.48E-04	6.12E-01
		GO:0018195	*peptidyl-arginine modification*	2.85E-04	8.85E-01
		GO:0048050	*post-embryonic eye morphogenesis*	4.54E-04	1.00E+00
		GO:0042398	*cellular modified amino acid biosynthetic process*	4.80E-04	9.93E-01
		GO:0048048	*embryonic eye morphogenesis*	4.85E-04	8.61E-01
		GO:0044283	*small molecule biosynthetic process*	5.92E-04	9.19E-01
		GO:0097193	*intrinsic apoptotic signalling pathway*	6.69E-04	9.24E-01
		GO:0003198	*epithelial to mesenchymal transition involved in endocardial cushion formation*	9.00E-04	1.00E+00
	95	GO:0090140	*regulation of mitochondrial fission*	4.60E-05	5.73E-01
		GO:0070098	*chemokine-mediated signalling pathway*	5.51E-05	3.43E-01
		GO:0044344	*cellular response to fibroblast growth factor stimulus*	3.78E-04	1.00E+00
		GO:0018101	*protein citrullination*	5.77E-04	1.00E+00
		GO:0000052	*citrulline metabolic process*	8.07E-04	1.00E+00

A)

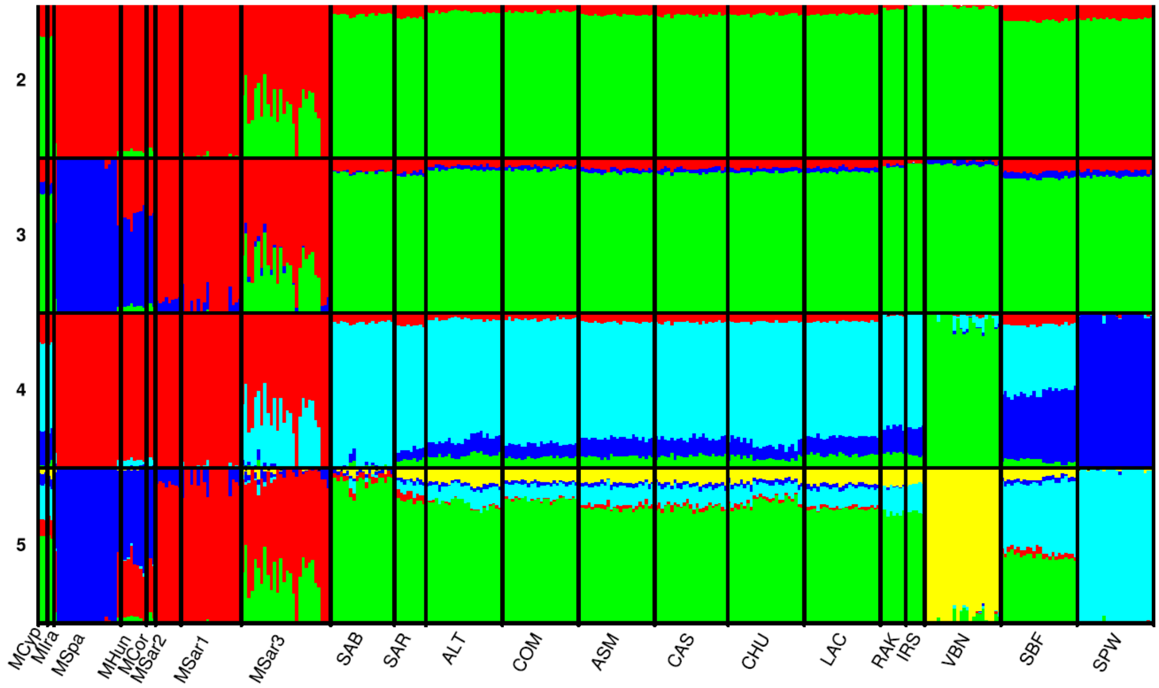
2
3
4
5
MCyp
MIra
MSpa
MHun
MCor
MSar2
MSar1
MSar3
SAB
SAR
ALT
COM
ASM
CAS
CHU
LAC
RAK
IRS
VBN
SBF
SPW

B)

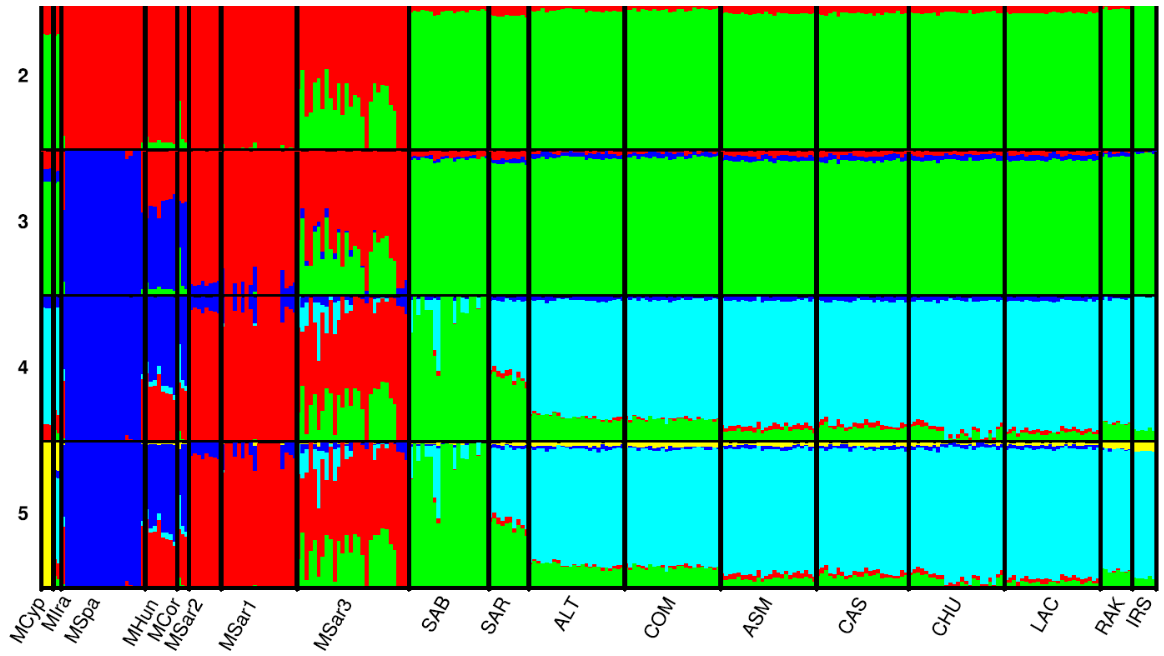
2
3
4
5
MCyp
MIra
MSpa
MHun
MCor
MSar2
MSar1
MSar3
SAB
SAR
ALT
COM
ASM
CAS
CHU
LAC
RAK
IRS

C)

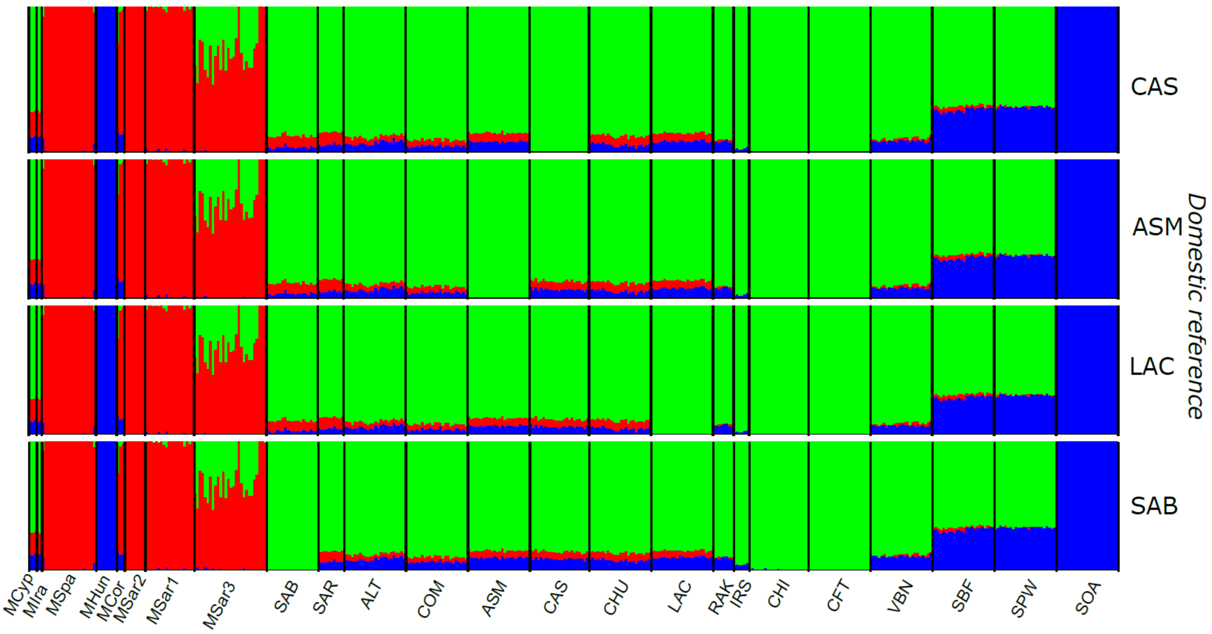

D)

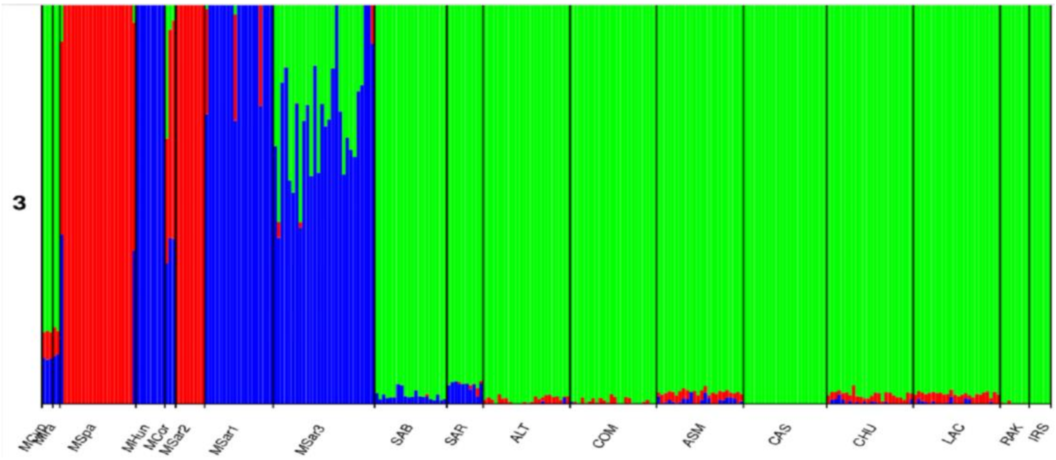

S1 Figure. Admixture plots.

Admixture plots (K = 2-5) after removal of SBF, SPW, SOA (A), and SBF, SPW, SOA, CHI, CFT, and VBN (B). C) Supervised analysis performed using the same reference populations used for the local ancestry analyses as starting ancestral populations. Four replicates were performed for each domestic reference used. D) Supervised analysis after removing SOA, SPW, SBF, SBN, CFT, and CHI.

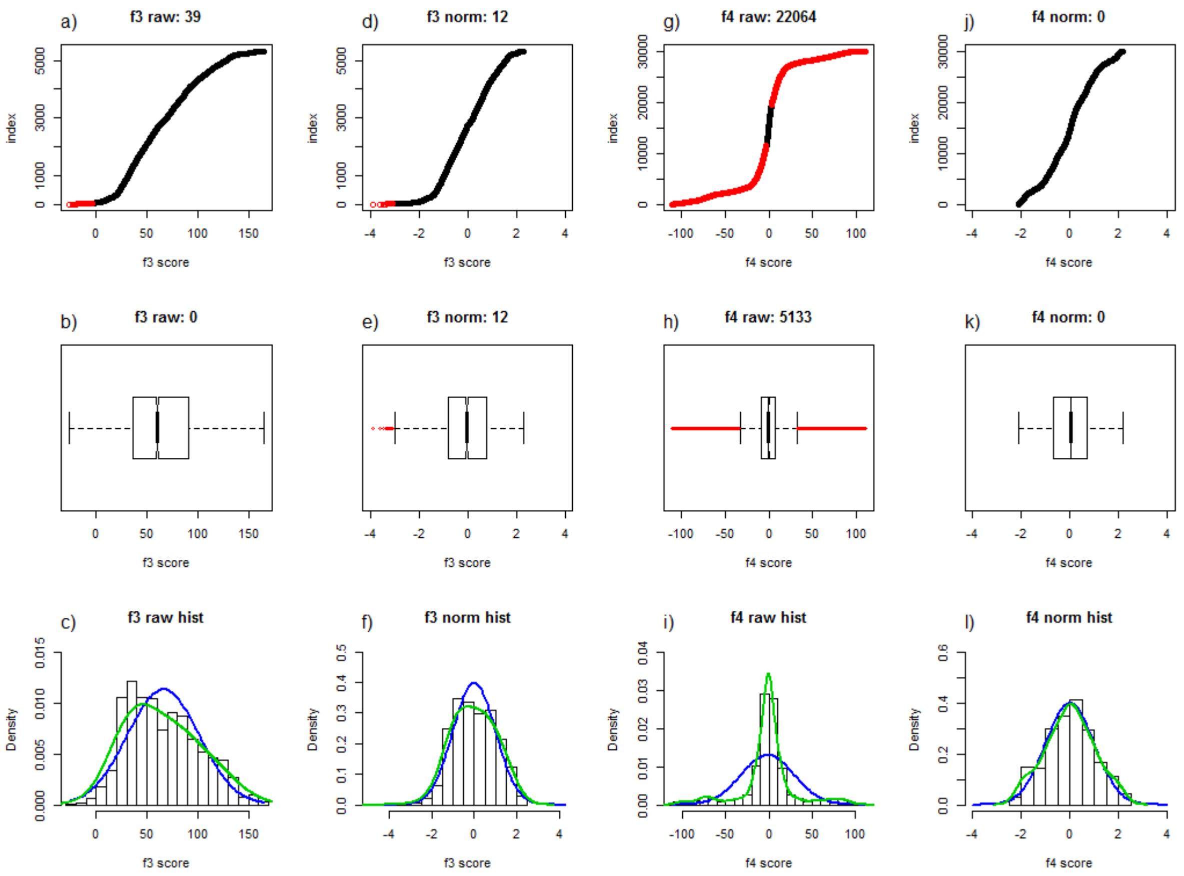

S2 Figure. f3/f4 score distribution and outlier selection.

The label 'raw' (plots a-b-c and g-h-i) refers to the non-transformed scores as obtained from the software used to calculate the f3/f4 statistics, whereas 'norm' (plots d-e-f and j-k-l) refers to the same statistics after normalization. In the top and central row are Cleveland plots (plots a-d-g-j), and Boxplots (plots b-e-h-k). The number of the most extreme values is shown in the title and highlighted in red in each graph. Extreme values in the Cleveland plots are selected as those values having |score|>3. Extreme values in the Boxplots are selected as those values lying outside 1.5x the interquartile range above the upper quartile and below the lower quartile. The histograms and green lines in the bottom row (plots c-f-i-l) display data distribution. The blue line shows the theoretical data distribution under the assumption of normality obtained using the same mean, standard deviation and number of observation as in the original data.

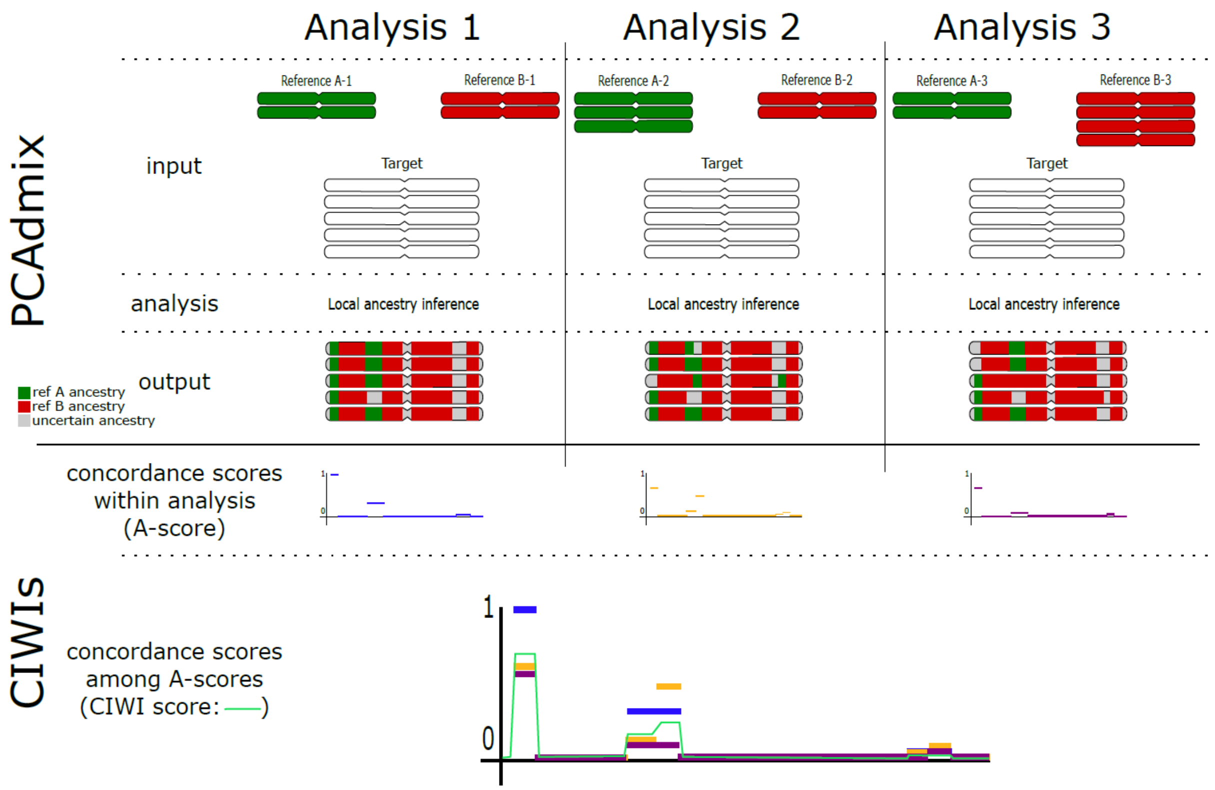

S3 Figure. Schematic outline of the 'Consistently Introgressed Windows of Interest (CIWI)' approach.

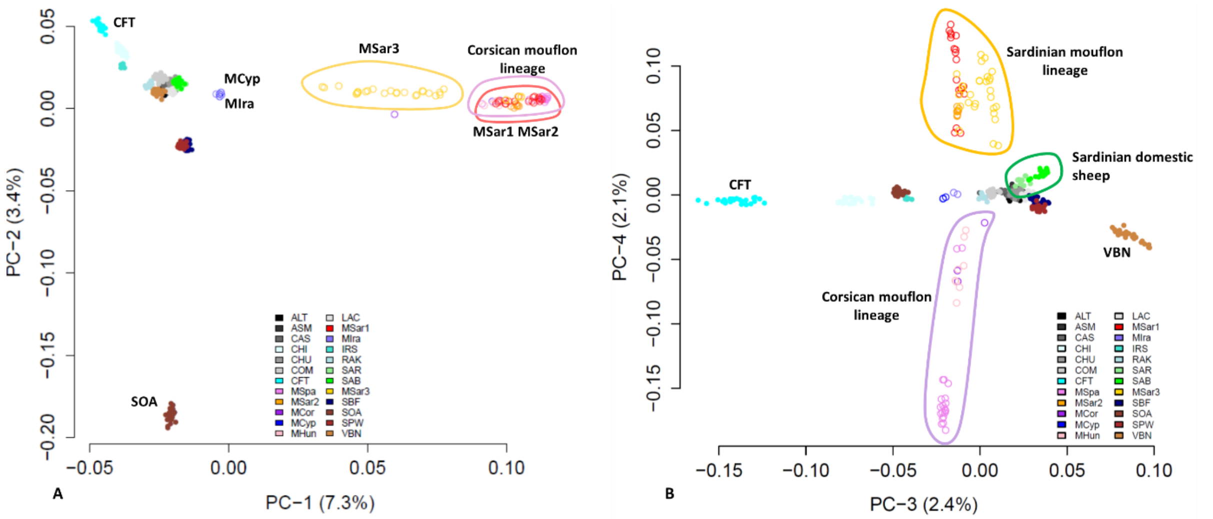

S4 Figure. PCA plot.

Principal component analysis of the dataset. The first four components (PC-1 – PC-4) are displayed. Mouflon individuals are identified by open circles whereas domestic sheep are identified by filled circles. For population abbreviations see Table 1.

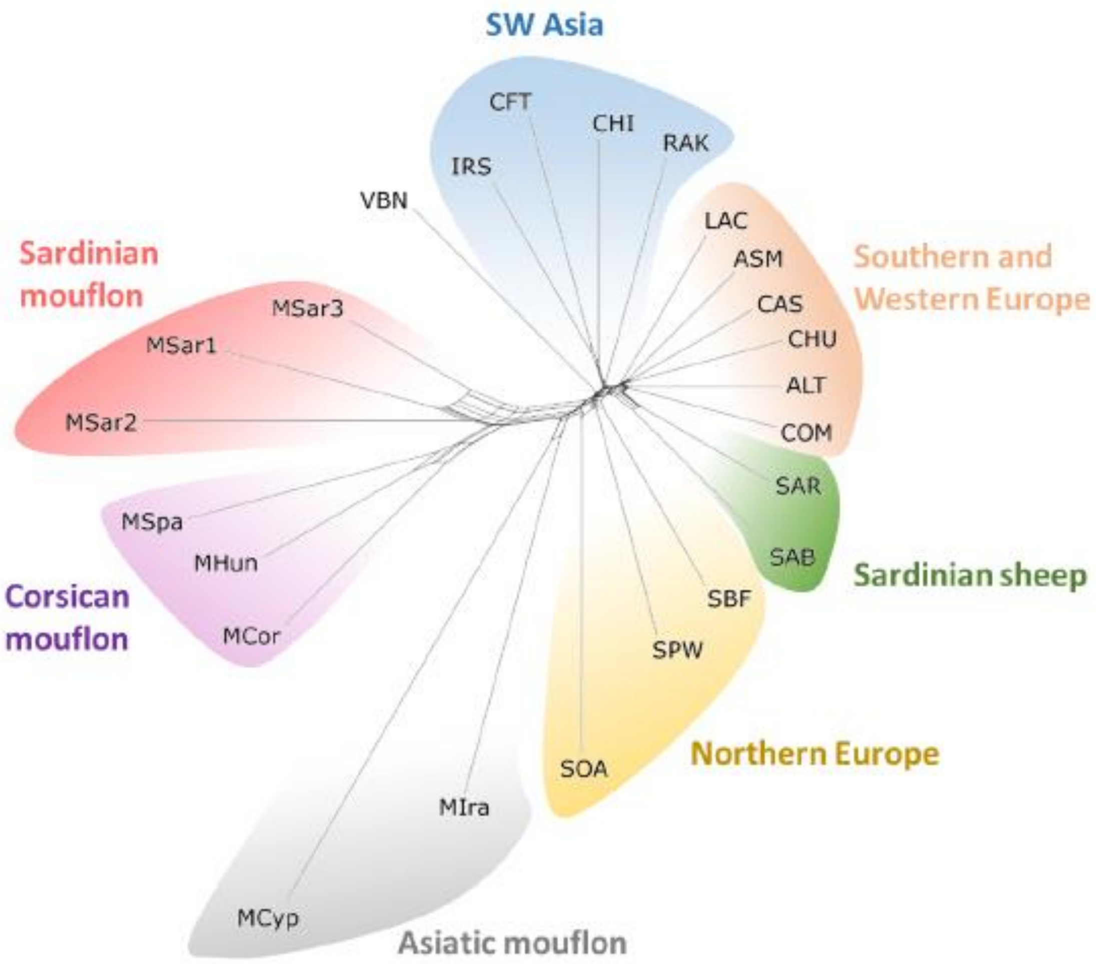

S5 Figure. Neighbour-Net plot.

Neighbour-Net from Splitstree4, based on pairwise Reynolds' distances.

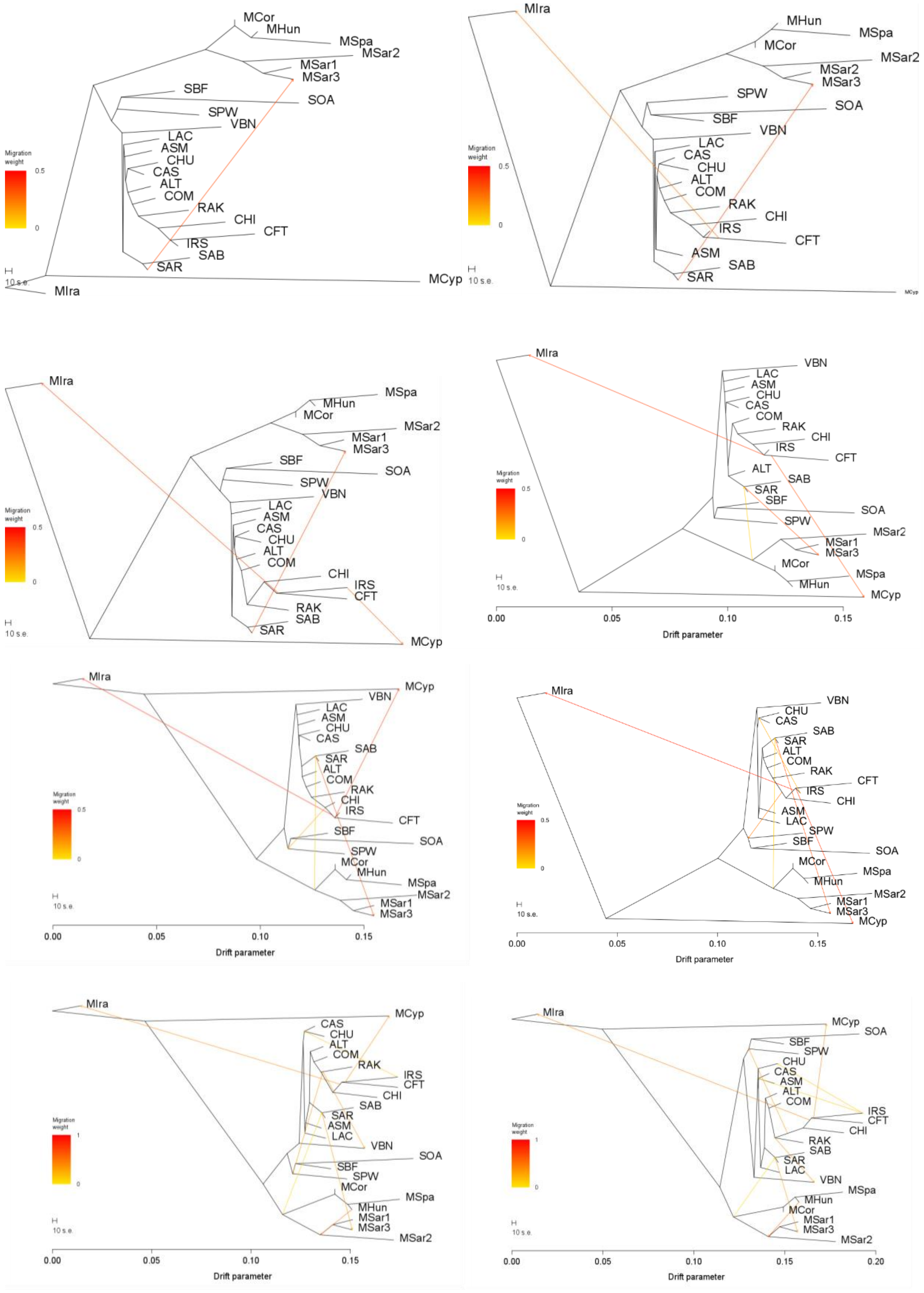

S6 Figure. Treemix plots.

S7 Figure. Graphical representation of PCAdmix results.

In each of the following pages the results obtained for a single population are displayed (abbreviation in top-left corner of each page). M2Sar (red) and MHun (blue) were always used as references for the Sardinian and Corsican mouflon lineage, respectively. For each population analysed four domestic sheep (green) reference populations were used: CAS (top-left image), ASM (top-right image), LAC (bottom-left) and SAB_p (bottom-right). Genomic regions assigned by PCAdmix with posterior probability <0.95 are displayed in grey. The horizontal axes represent the chromosome size in bp. The numbers in the vertical axis identifies the chromosomes. Each line within a chromosome, represents a haploid individual.

MSar1

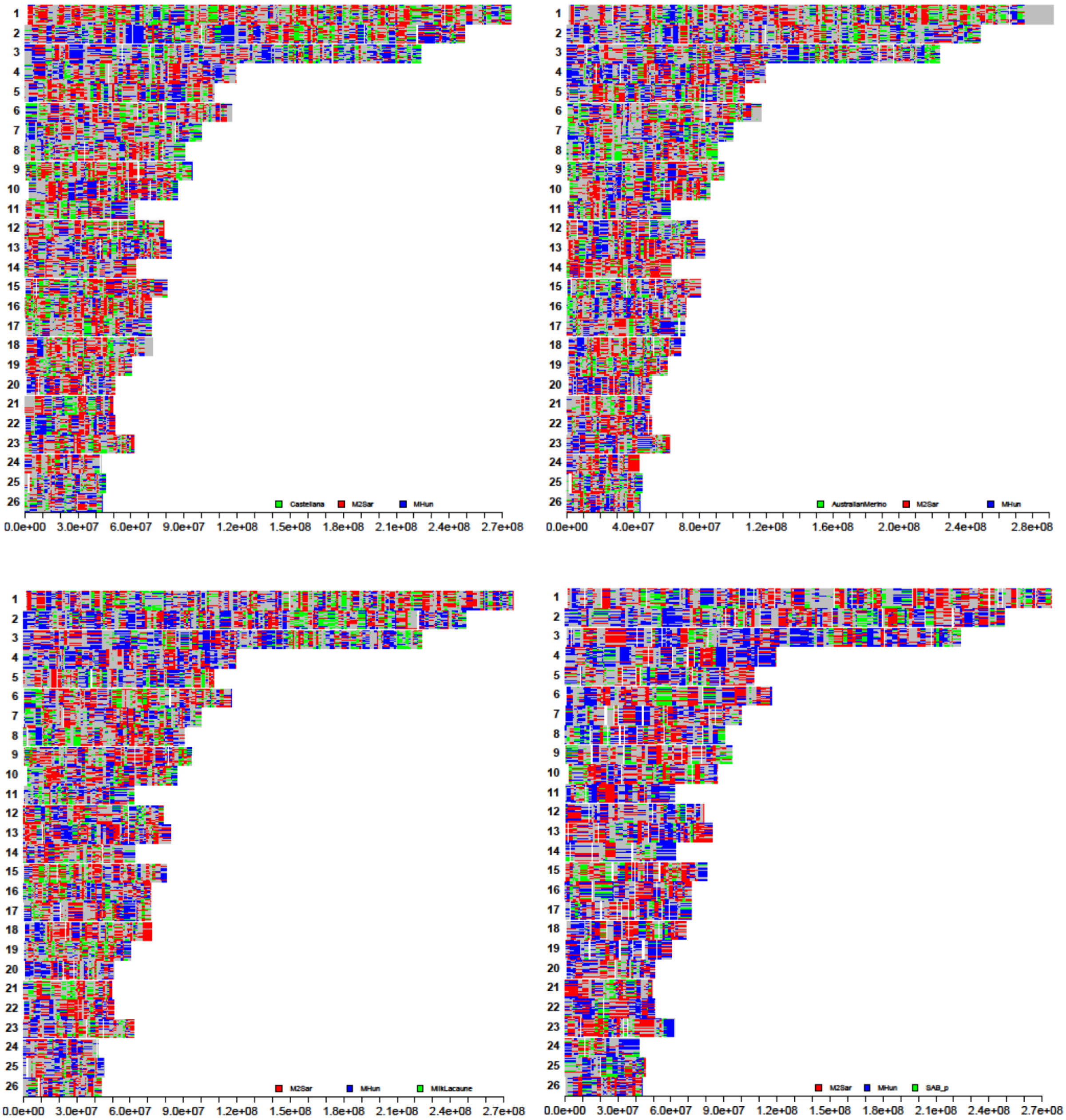
Castellana
M2Sar
MHun
AustralianMerino
M2Sar
MHun
M2Sar
MHun
MilkLacaune
M2Sar
MHun
SAB_p

MSar3

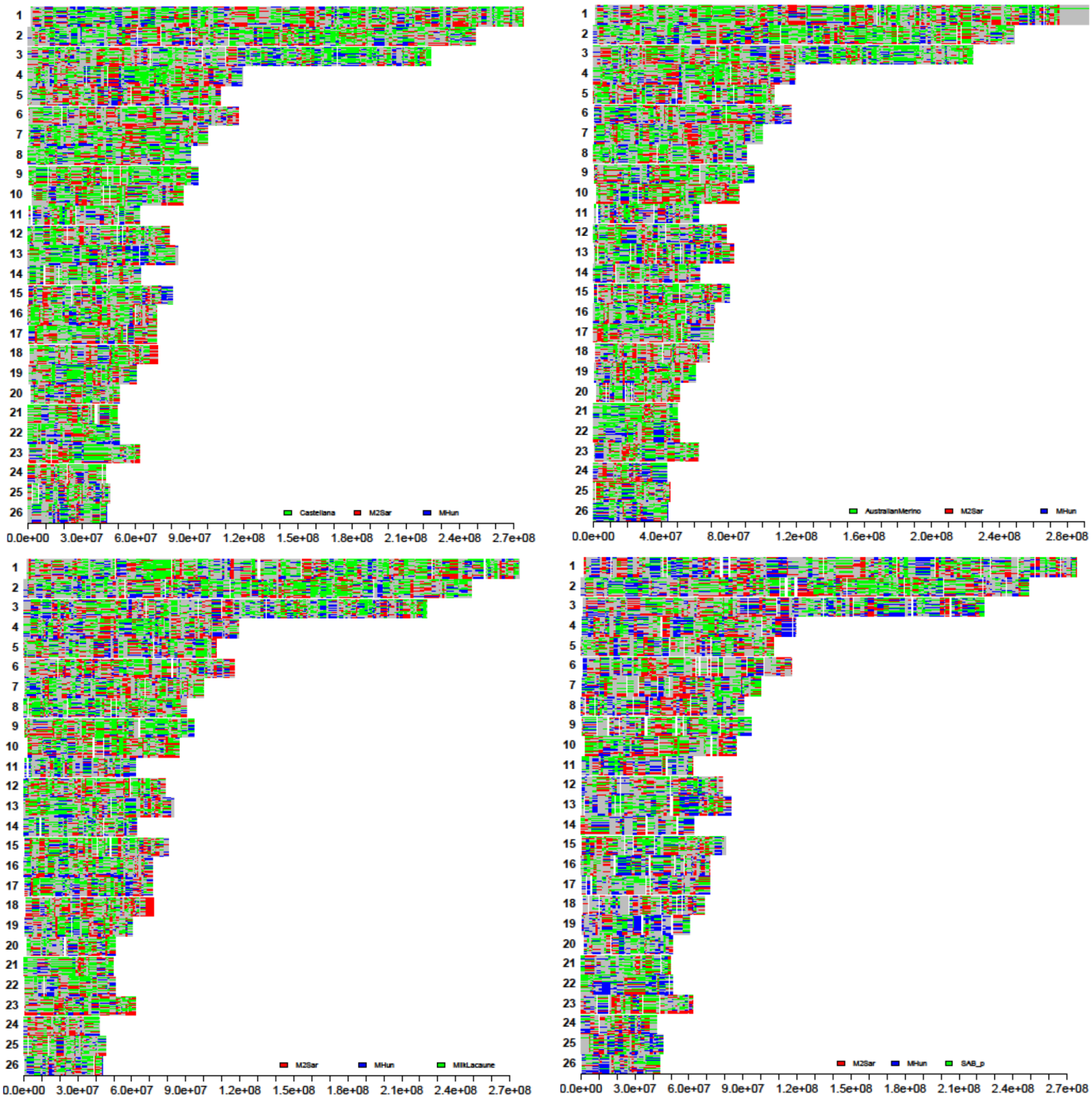
Castellana
M2Sar
MHun
AustralianMerino
M2Sar
MHun
M2Sar
MHun
MilkLacaune
M2Sar
MHun
SAB_p

MSpa

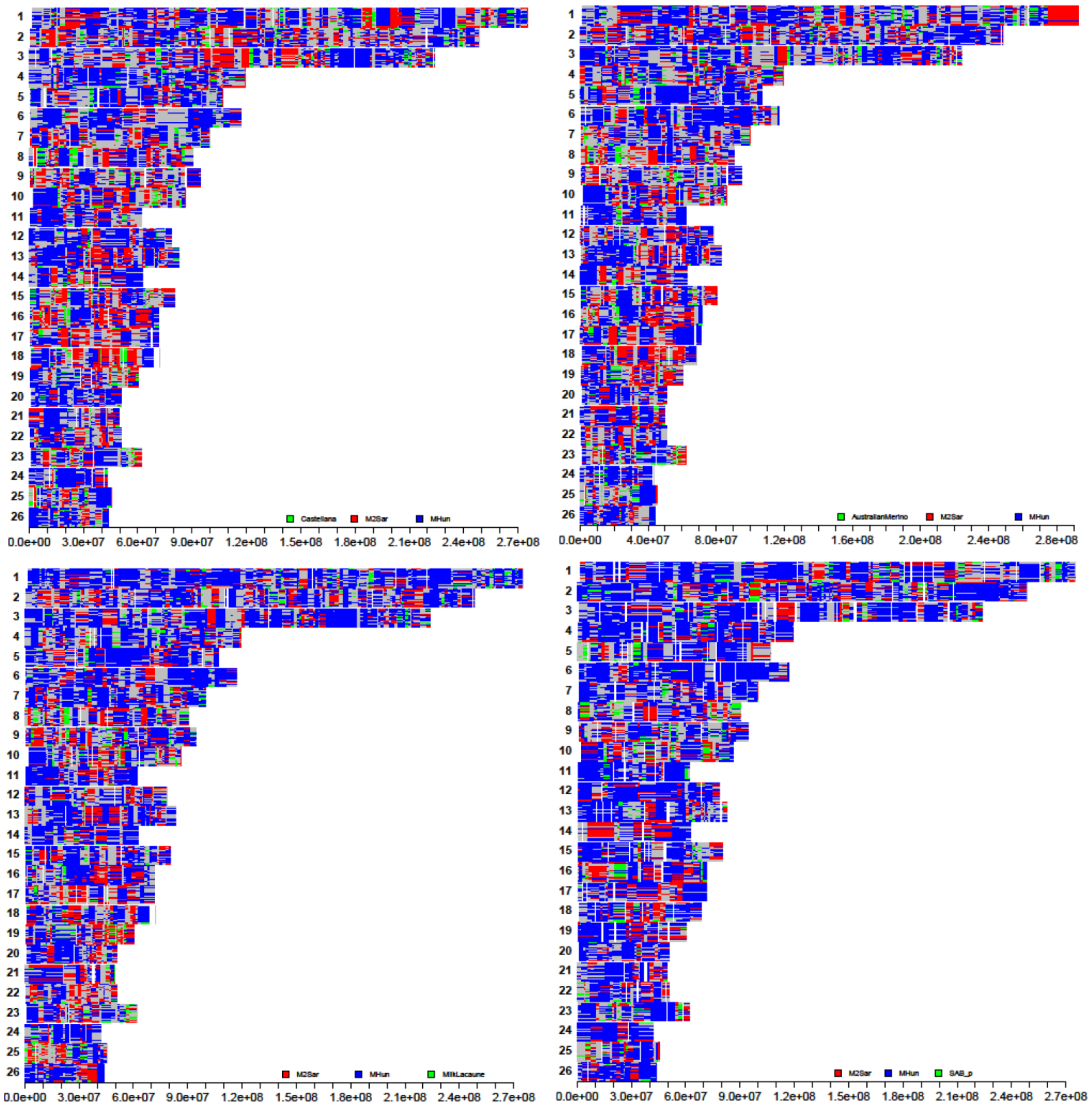
Castellana
M2Sar
MHun
0.0e+00 3.0e+07 6.0e+07 9.0e+07 1.2e+08 1.5e+08 1.8e+08 2.1e+08 2.4e+08 2.7e+08
AustralianMerino
M2Sar
MHun
0.0e+00 4.0e+07 8.0e+07 1.2e+08 1.6e+08 2.0e+08 2.4e+08 2.8e+08
M2Sar
MHun
MilkLacaune
0.0e+00 3.0e+07 6.0e+07 9.0e+07 1.2e+08 1.5e+08 1.8e+08 2.1e+08 2.4e+08 2.7e+08
M2Sar
MHun
SAB_p
0.0e+00 3.0e+07 6.0e+07 9.0e+07 1.2e+08 1.5e+08 1.8e+08 2.1e+08 2.4e+08 2.7e+08

ALT

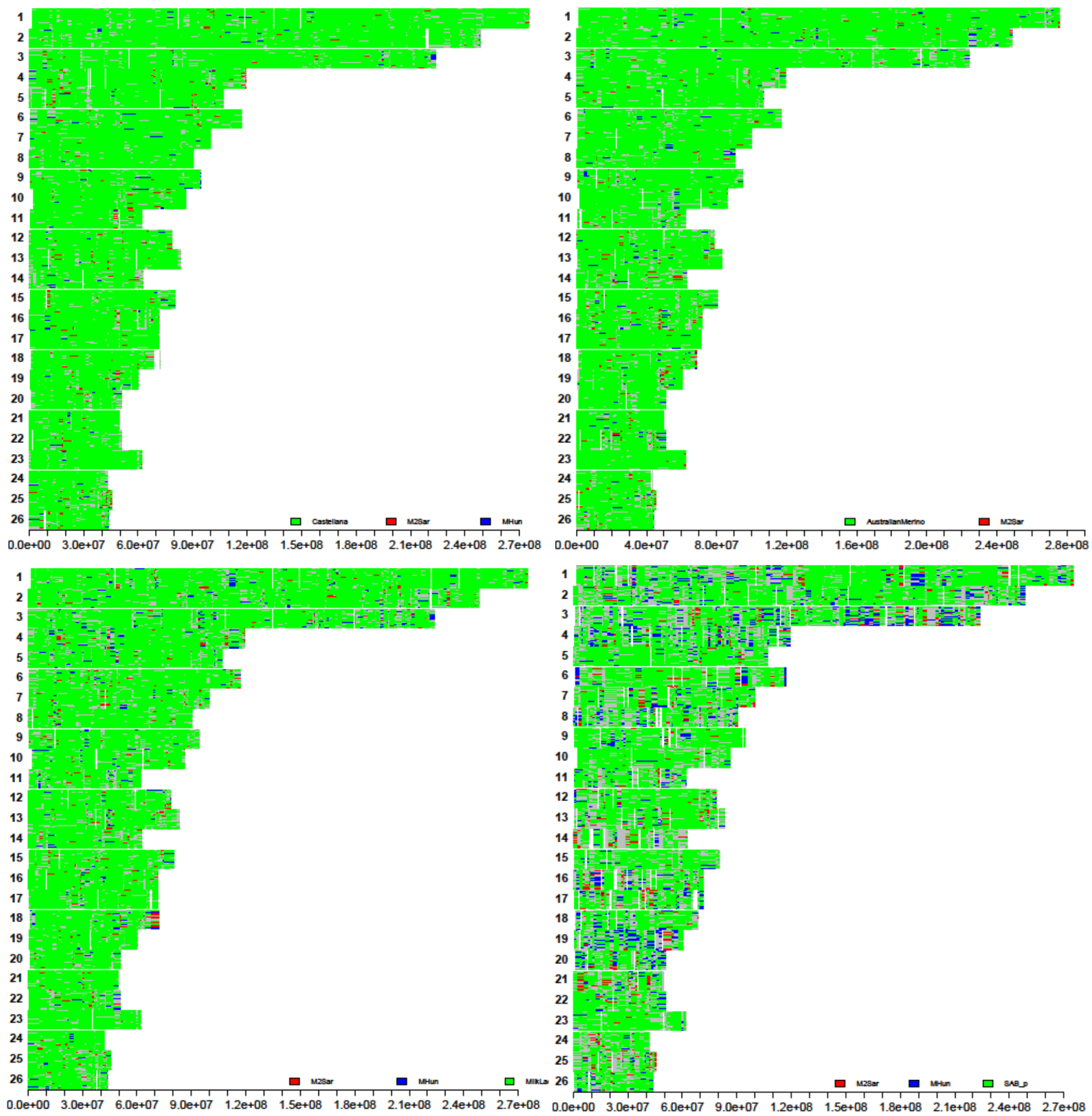
Castellana
M2Sar
MHun
AustralianMerino
M2Sar
M2Sar
MHun
M2Sar
MHun
SAB_p

CHI

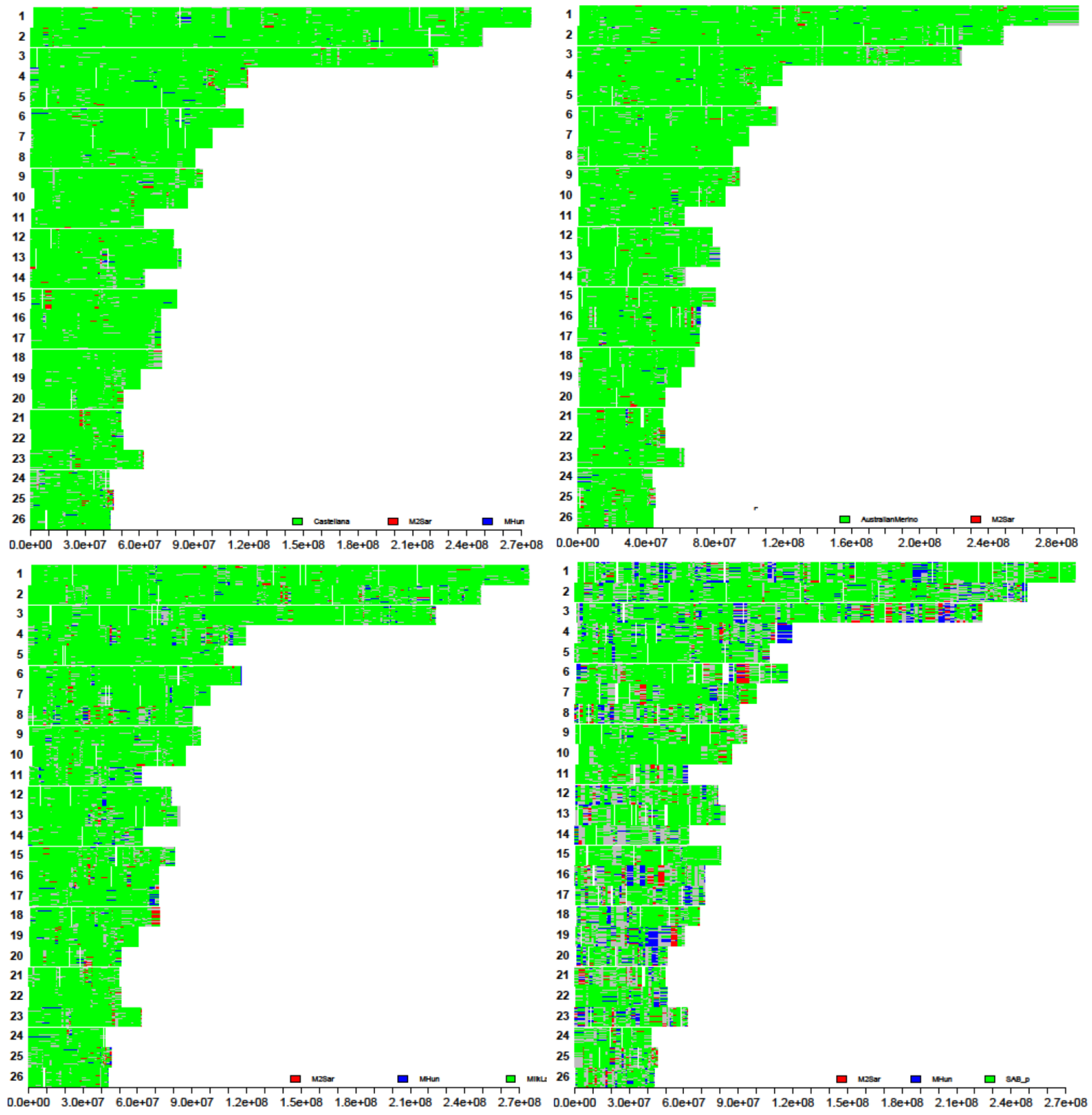
Castellana
M2Sar
MHun
AustralianMerino
M2Sar
M2Sar
MHun
MIlkL
M2Sar
MHun
SAB_p
0.0e+00
3.0e+07
6.0e+07
9.0e+07
1.2e+08
1.5e+08
1.8e+08
2.1e+08
2.4e+08
2.7e+08
4.0e+07
8.0e+07
1.6e+08
2.0e+08
2.8e+08

CHU

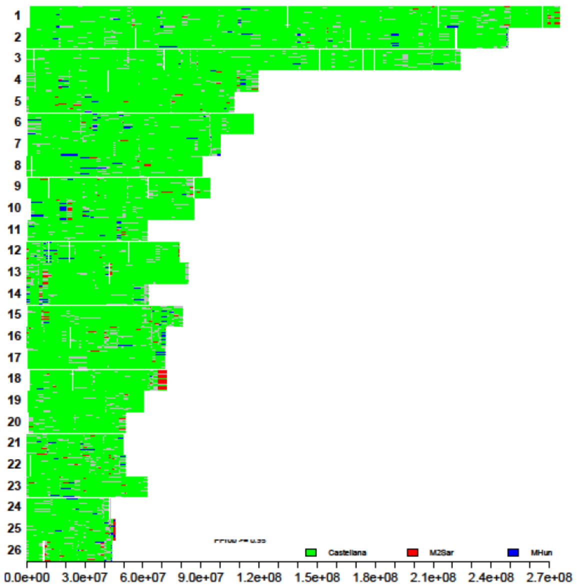
1 2 3 4 5 6 7 8 9 10 11 12 13 14 15 16 17 18 19 20 21 22 23 24 25 26
Castellana
M2Sar
MHun
0.0e+00 3.0e+07 6.0e+07 9.0e+07 1.2e+08 1.5e+08 1.8e+08 2.1e+08 2.4e+08 2.7e+08

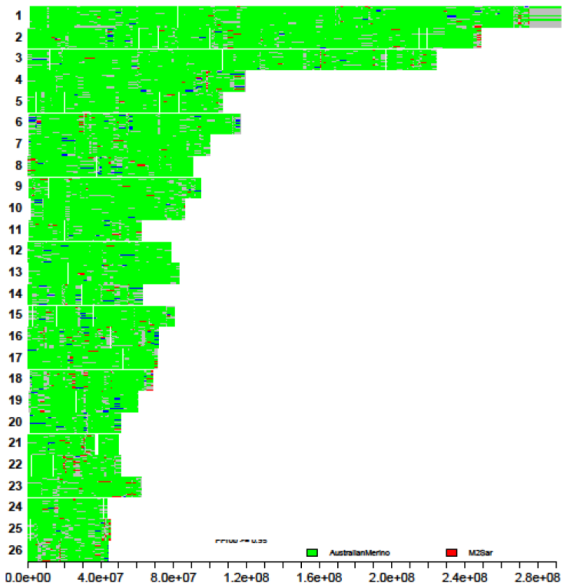
1 2 3 4 5 6 7 8 9 10 11 12 13 14 15 16 17 18 19 20 21 22 23 24 25 26
AustralianMerino
M2Sar
0.0e+00 4.0e+07 8.0e+07 1.2e+08 1.6e+08 2.0e+08 2.4e+08 2.8e+08

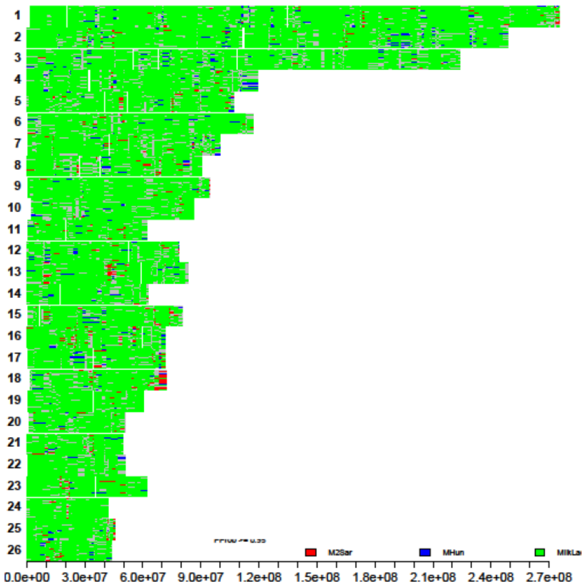
1 2 3 4 5 6 7 8 9 10 11 12 13 14 15 16 17 18 19 20 21 22 23 24 25 26
M2Sar
MHun
MilkLa
0.0e+00 3.0e+07 6.0e+07 9.0e+07 1.2e+08 1.5e+08 1.8e+08 2.1e+08 2.4e+08 2.7e+08

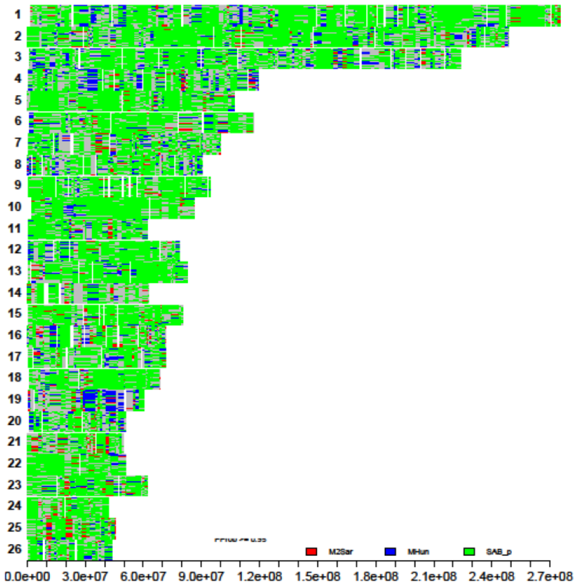
1 2 3 4 5 6 7 8 9 10 11 12 13 14 15 16 17 18 19 20 21 22 23 24 25 26
M2Sar
MHun
SAB_p
0.0e+00 3.0e+07 6.0e+07 9.0e+07 1.2e+08 1.5e+08 1.8e+08 2.1e+08 2.4e+08 2.7e+08

7

COM

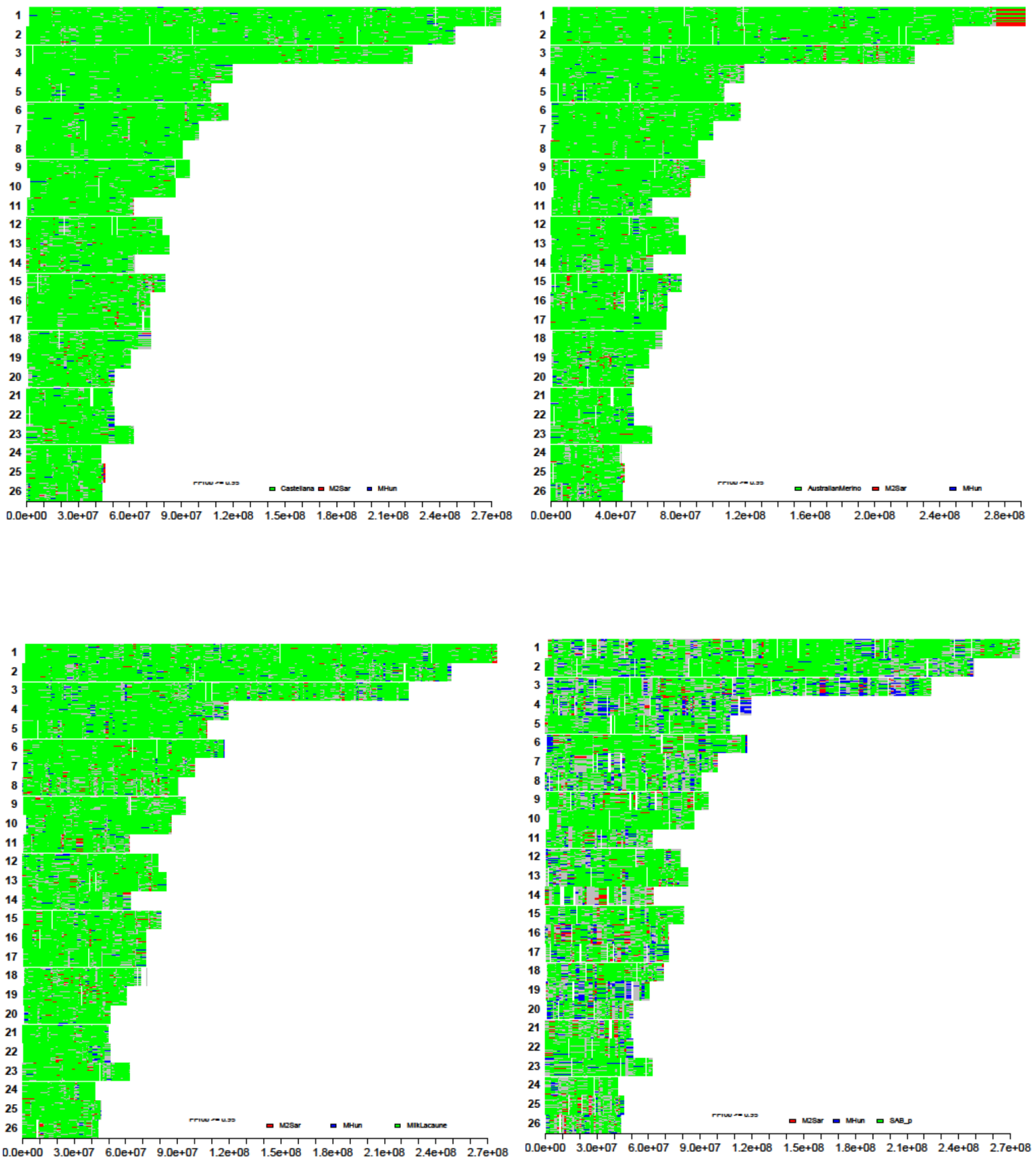
Castellana
M2Sar
MHun
AustralianMerino
M2Sar
MHun
M2Sar
MHun
MilkLacaune
M2Sar
MHun
SAB_p

CFT

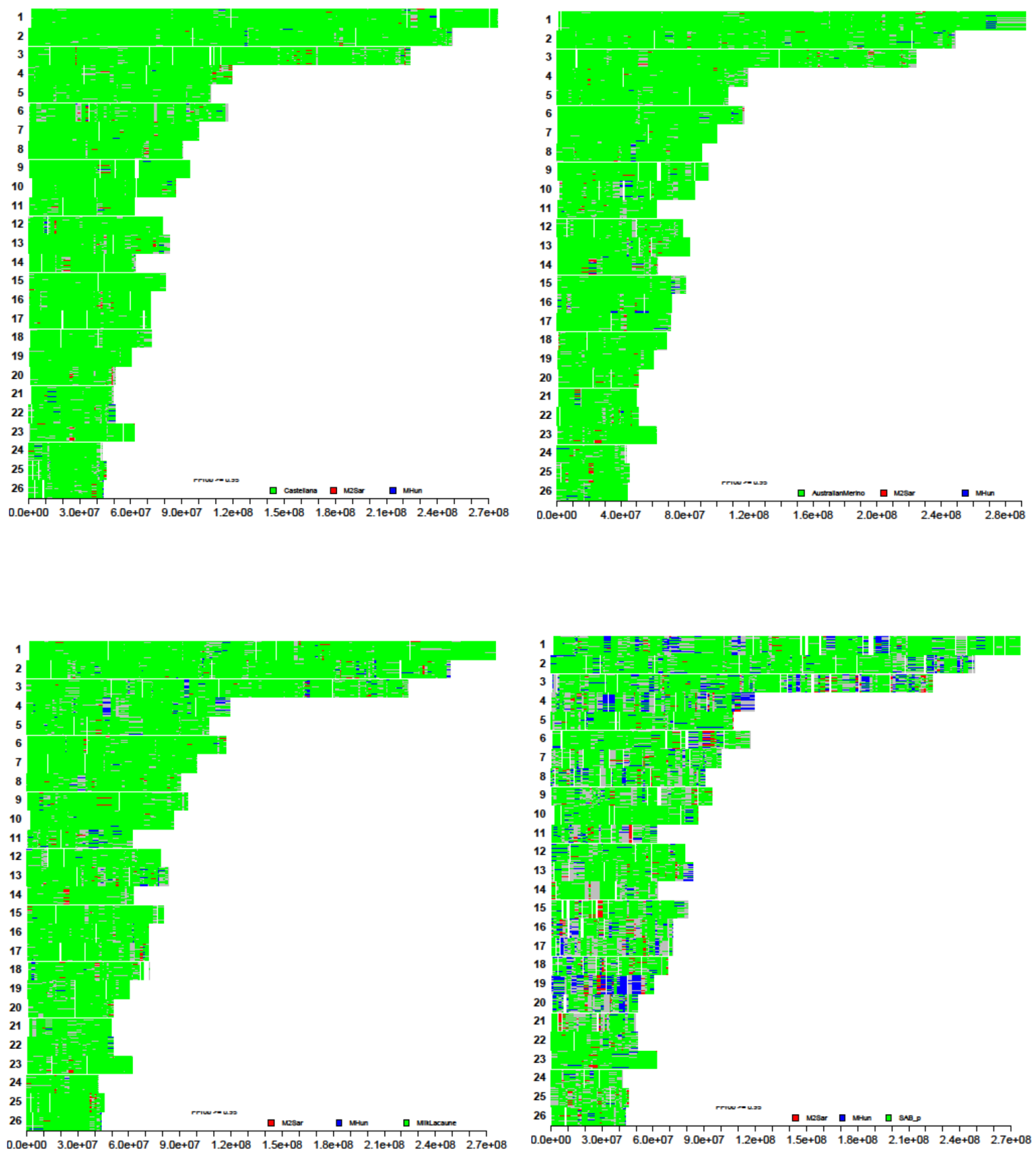
Castellana
M2Sar
MHun
AustralianMerino
M2Sar
MHun
M2Sar
MHun
MilkLacaune
M2Sar
MHun
SAB_p

RAK

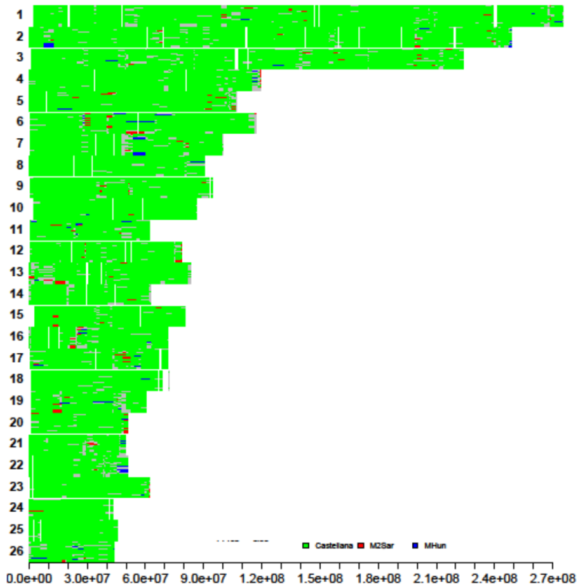
Castellana
M2Sar
MHun

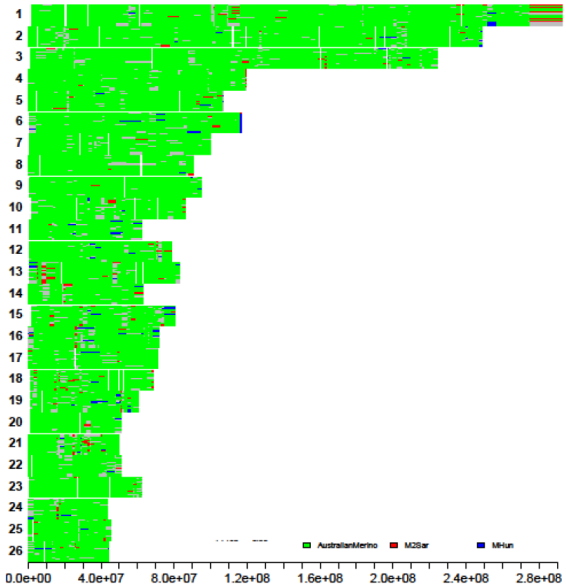
AustralianMerino
M2Sar
MHun

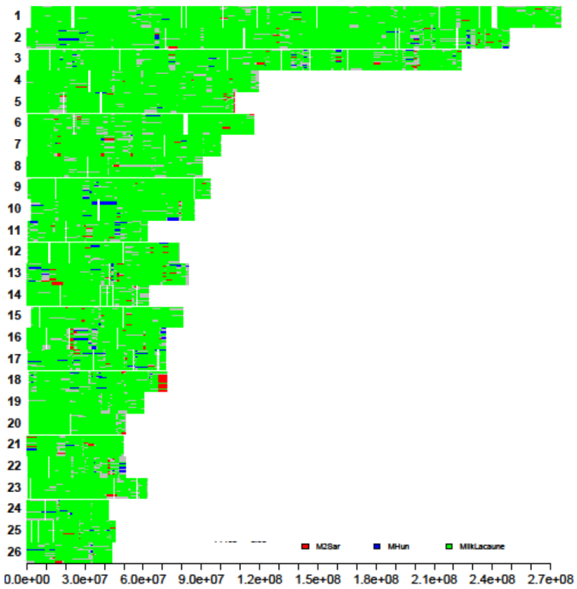
M2Sar
MHun
MilkLacaune

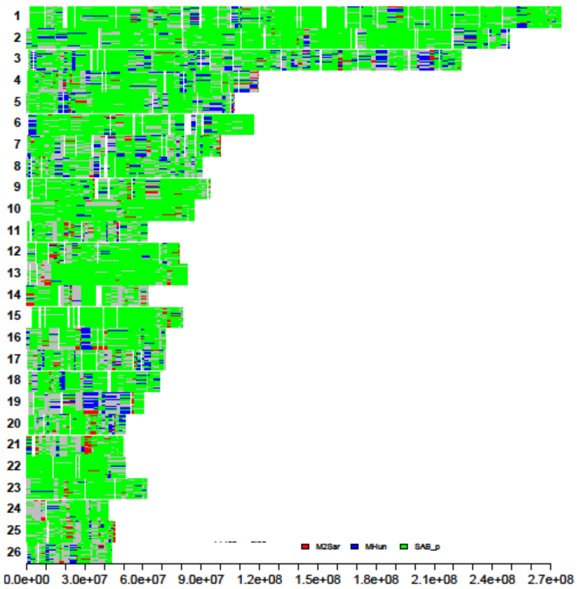
M2Sar
MHun
SAB_p

SAR

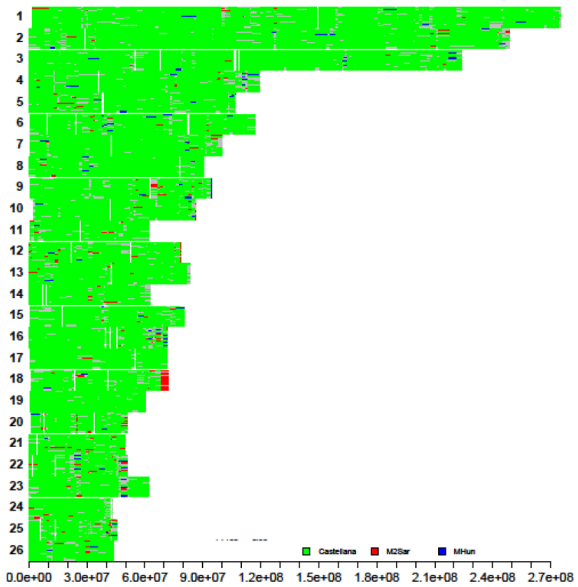
Castellana
M2Sar
MHun

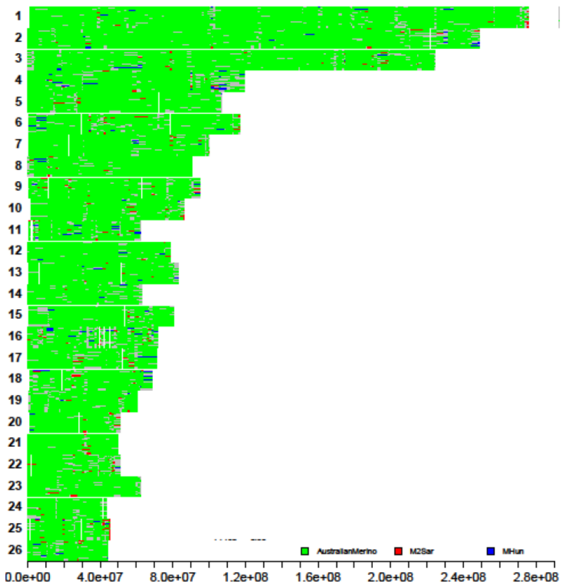
AustralianMerino
M2Sar
MHun

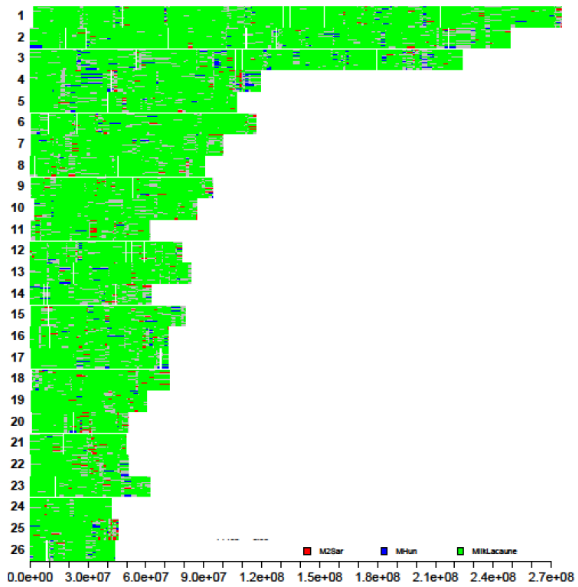
M2Sar
MHun
MilkLacaune

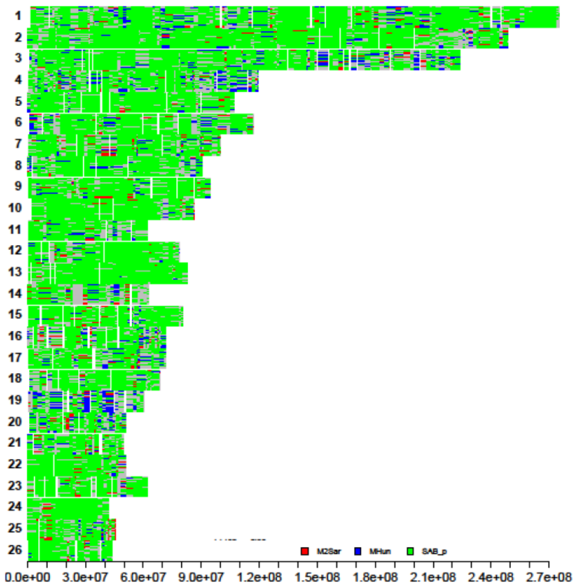
M2Sar
MHun
SAB_p

SBF

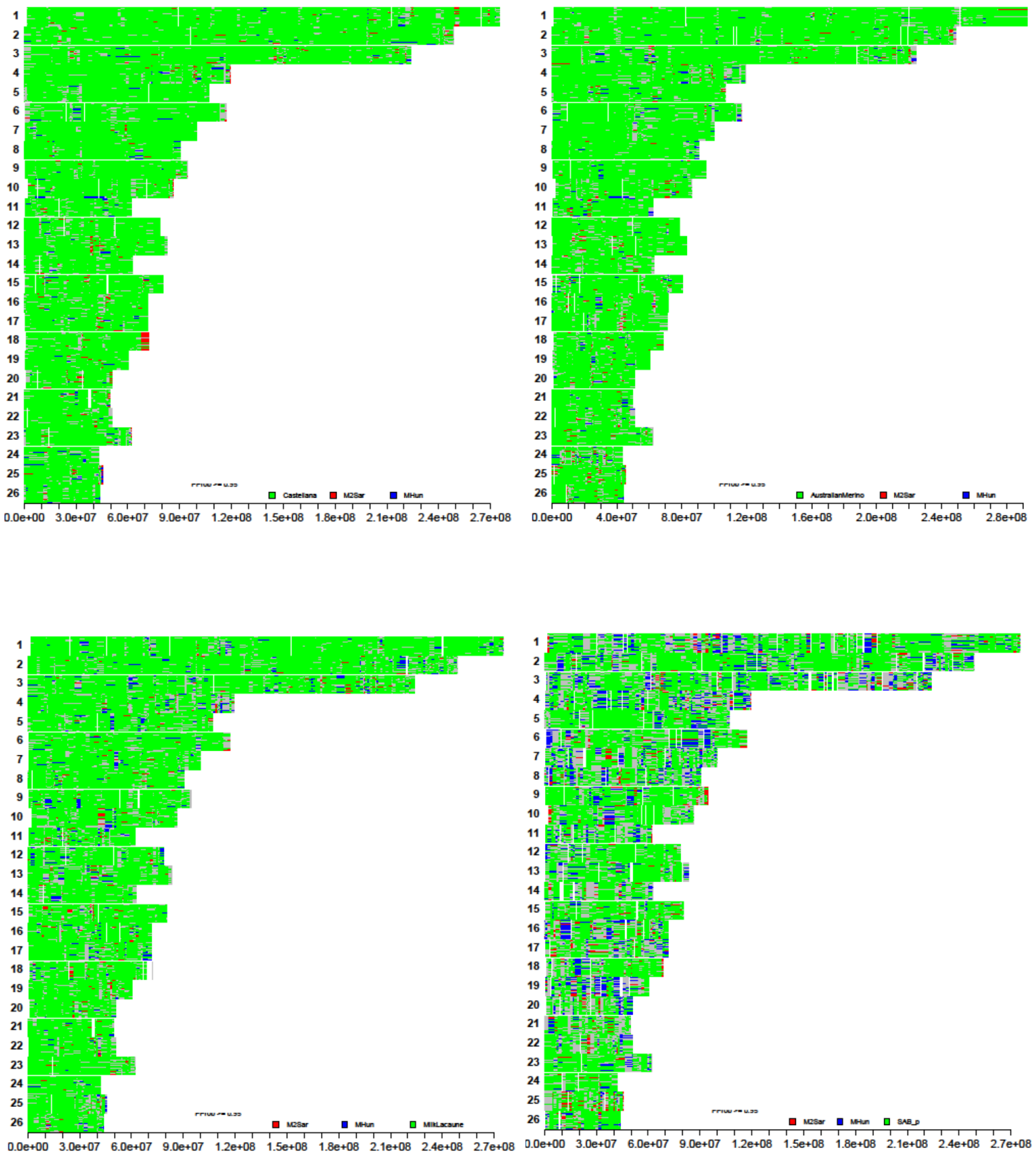
Castellana
M2Sar
MHun
AustralianMerino
M2Sar
MHun
M2Sar
MHun
MilkLacaune
M2Sar
MHun
SAB_p

SOA

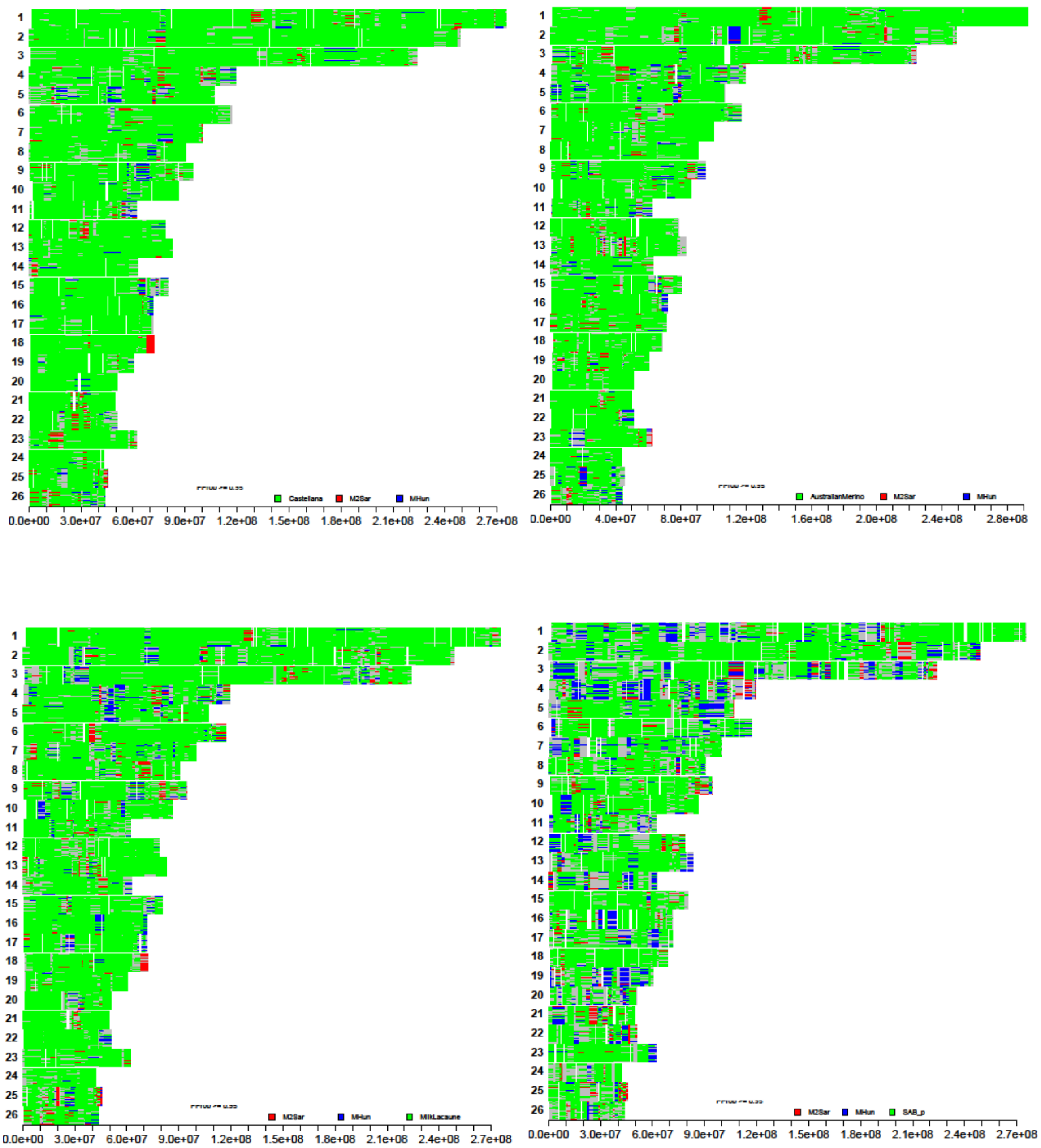
Castellana
M2Sar
MHun
AustralianMerino
M2Sar
MHun
M2Sar
MHun
MilkLacaune
M2Sar
MHun
SAB_p

SPW

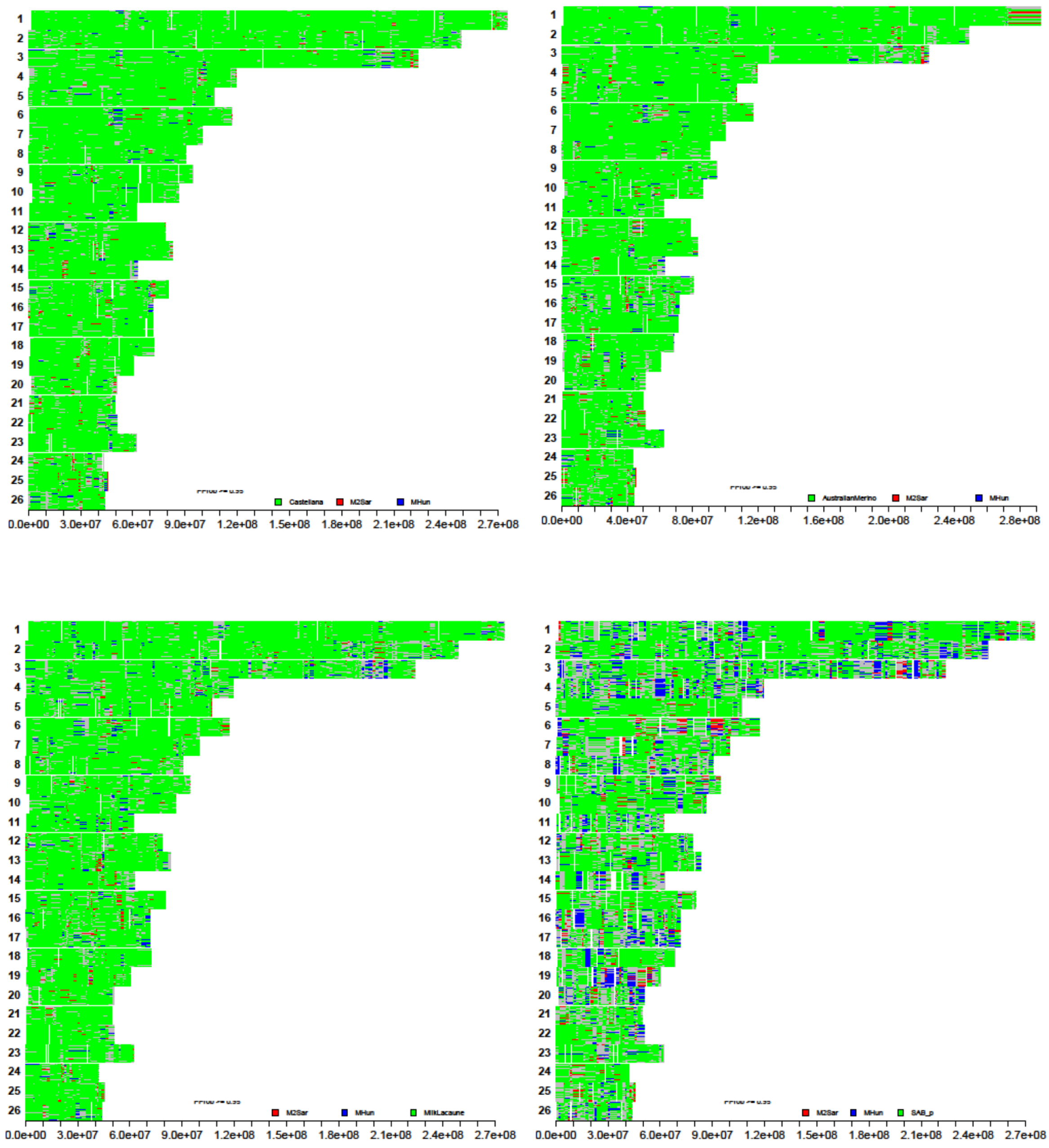
Castellana
M2Sar
MHun
0.0e+00 3.0e+07 6.0e+07 9.0e+07 1.2e+08 1.5e+08 1.8e+08 2.1e+08 2.4e+08 2.7e+08
AustralianMerino
M2Sar
MHun
0.0e+00 4.0e+07 8.0e+07 1.2e+08 1.6e+08 2.0e+08 2.4e+08 2.8e+08
M2Sar
MHun
MilkLacaune
0.0e+00 3.0e+07 6.0e+07 9.0e+07 1.2e+08 1.5e+08 1.8e+08 2.1e+08 2.4e+08 2.7e+08
M2Sar
MHun
SAB_p
0.0e+00 3.0e+07 6.0e+07 9.0e+07 1.2e+08 1.5e+08 1.8e+08 2.1e+08 2.4e+08 2.7e+08

VBN

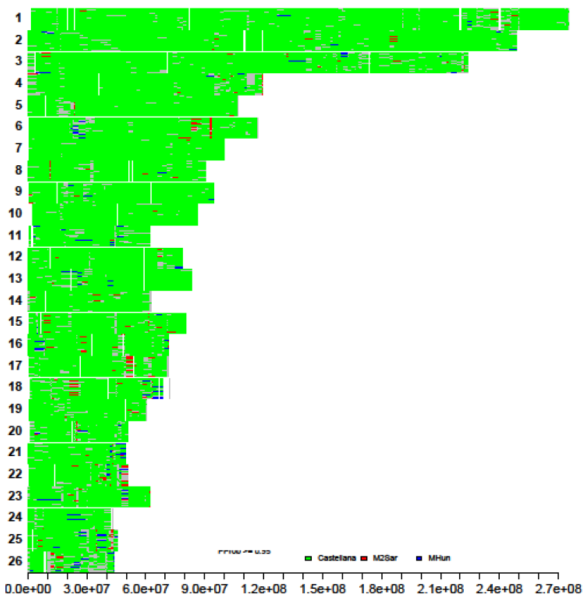
Castellana
M2Sar
MHun

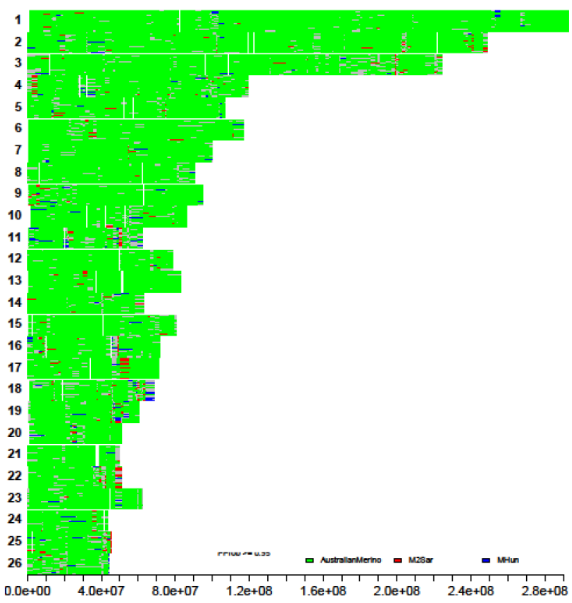
AustralianMerino
M2Sar
MHun

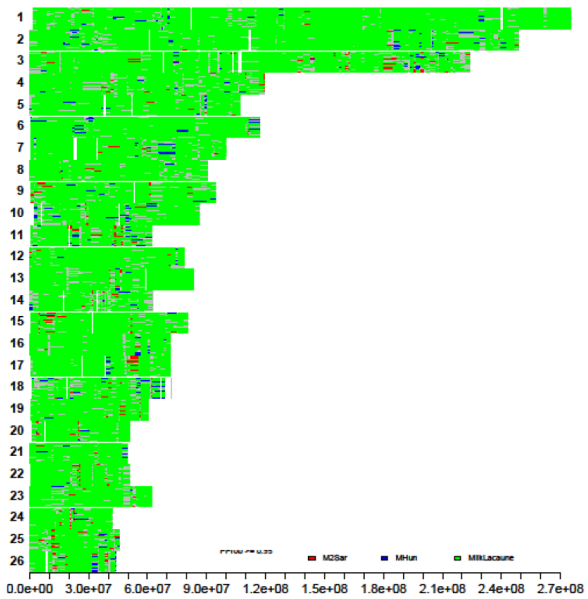
M2Sar
MHun
MilkLacaune

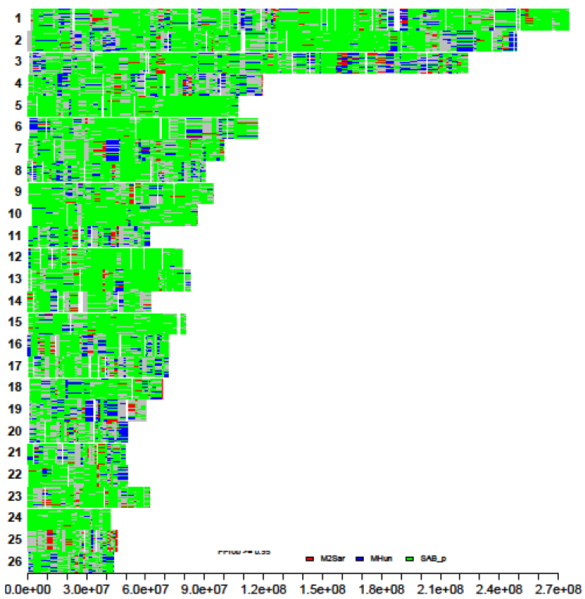
M2Sar
MHun
SAB_p

S8 Figure. Consistently Introgressed Windows of Interest (CIWI).

In each of the following pages the results obtained for a single population are displayed (abbreviation in top-left corner of each page). In each page the CIWI obtained for each of the 26 autosomes are displayed. The horizontal axis represents the chromosome size expressed in bp. The vertical axis represent the CIWI score. Within each graph, the segments represent the A-scores obtained using the four reference combinations (each reference set is identified by a different colour). The CIWI score is represented by the black line. For mouflon populations the CIWIs represent concordance of sheep ancestry, whereas for sheep populations they indicate mouflon ancestry.

MSar1

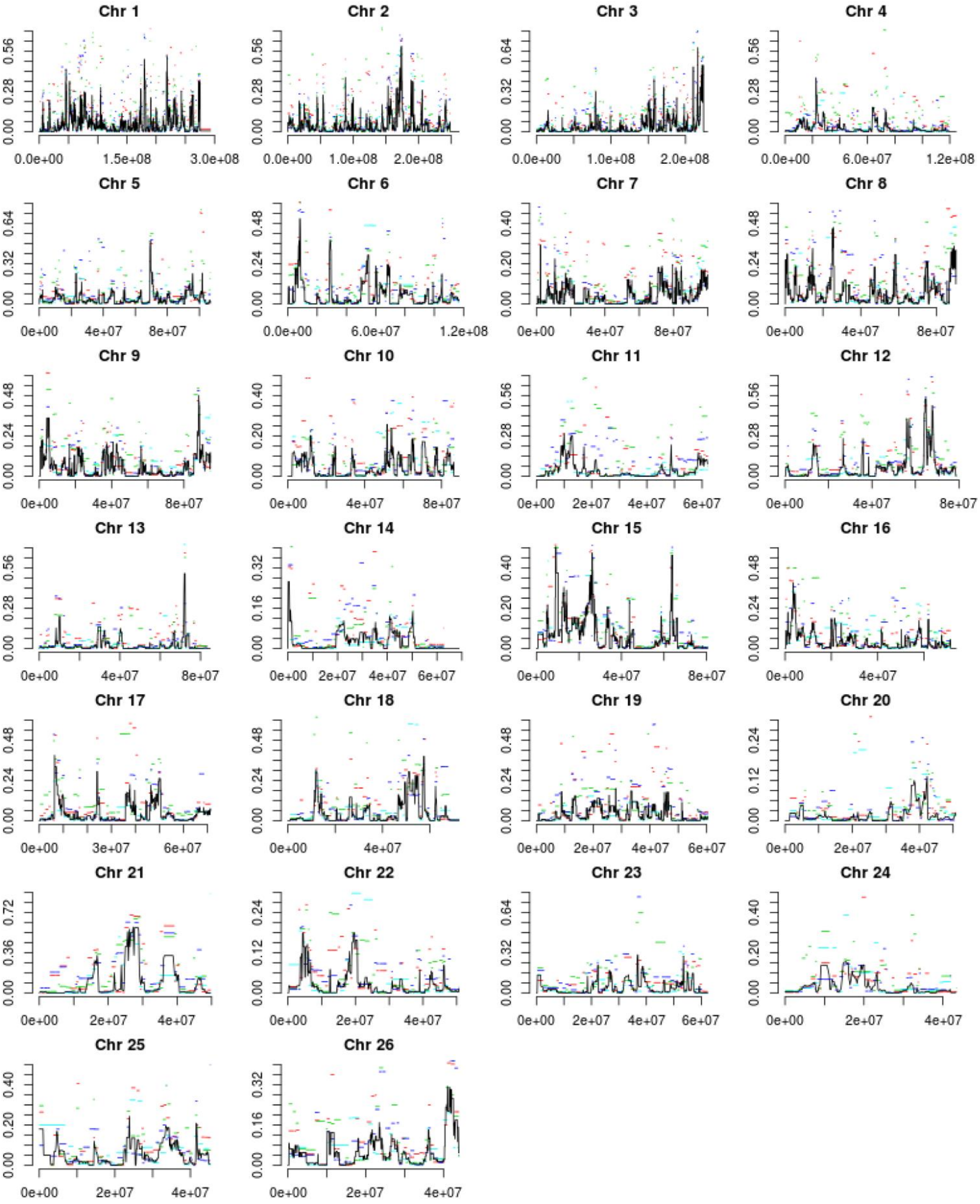

Chr 1
Chr 2
Chr 3
Chr 4
Chr 5
Chr 6
Chr 7
Chr 8
Chr 9
Chr 10
Chr 11
Chr 12
Chr 13
Chr 14
Chr 15
Chr 16
Chr 17
Chr 18
Chr 19
Chr 20
Chr 21
Chr 22
Chr 23
Chr 24
Chr 25
Chr 26

MSar3

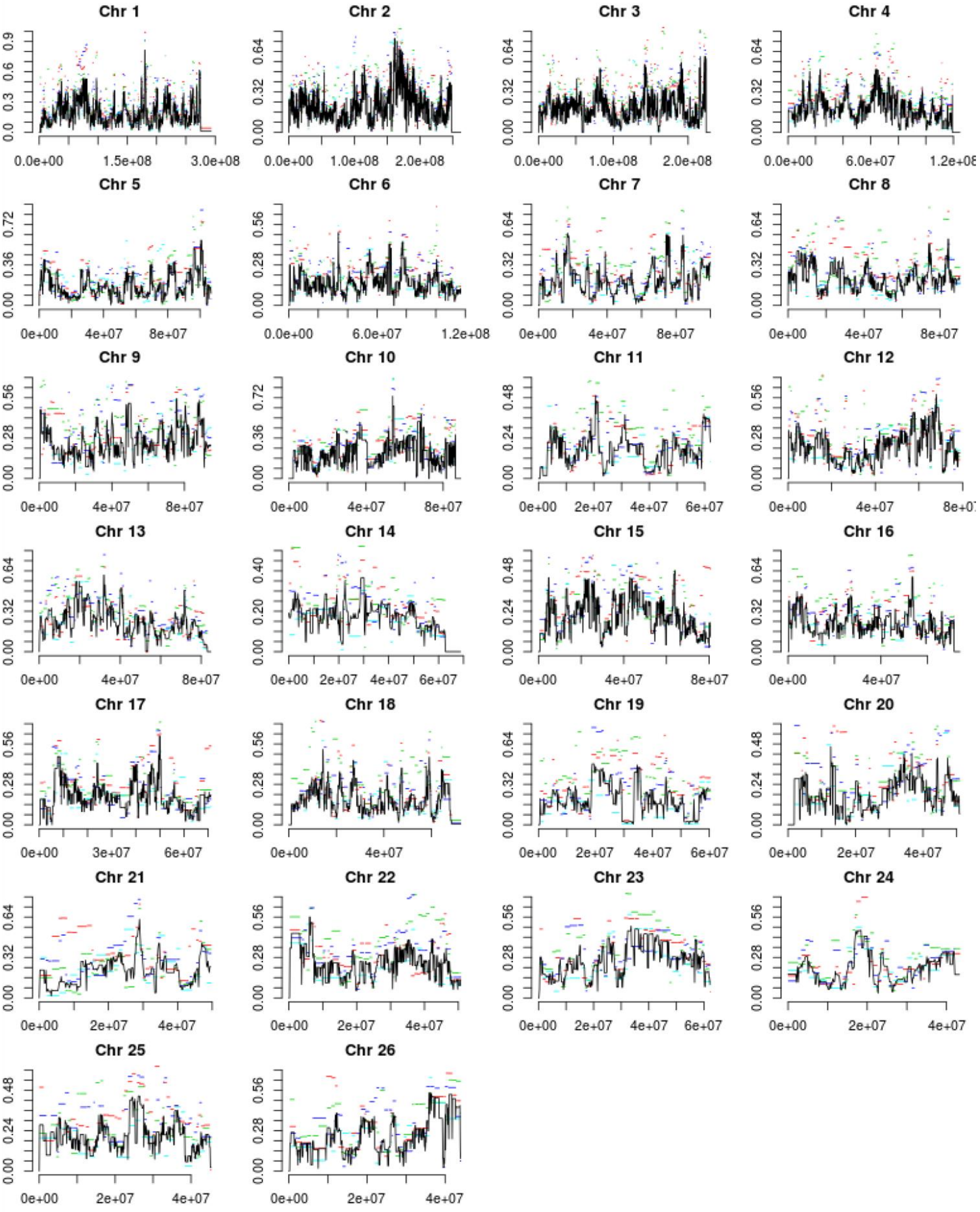
Chr 1
0.0 0.3 0.6 0.9
0.0e+00 1.5e+08 3.0e+08
Chr 2
0.00 0.32 0.64
0.0e+00 1.0e+08 2.0e+08
Chr 3
0.00 0.32 0.64
0.0e+00 1.0e+08 2.0e+08
Chr 4
0.00 0.32 0.64
0.0e+00 6.0e+07 1.2e+08
Chr 5
0.00 0.36 0.72
0e+00 4e+07 8e+07
Chr 6
0.00 0.28 0.56
0.0e+00 6.0e+07 1.2e+08
Chr 7
0.00 0.32 0.64
0e+00 4e+07 8e+07
Chr 8
0.00 0.32 0.64
0e+00 4e+07 8e+07
Chr 9
0.00 0.28 0.56
0e+00 4e+07 8e+07
Chr 10
0.00 0.36 0.72
0e+00 4e+07 8e+07
Chr 11
0.00 0.24 0.48
0e+00 2e+07 4e+07 6e+07
Chr 12
0.00 0.28 0.56
0e+00 4e+07
Chr 13
0.00 0.32 0.64
0e+00 4e+07 8e+07
Chr 14
0.00 0.20 0.40
0e+00 2e+07 4e+07 6e+07
Chr 15
0.00 0.24 0.48
0e+00 4e+07 8e+07
Chr 16
0.00 0.32 0.64
0e+00 4e+07
Chr 17
0.00 0.28 0.56
0e+00 3e+07 6e+07
Chr 18
0.00 0.28 0.56
0e+00 4e+07
Chr 19
0.00 0.32 0.64
0e+00 2e+07 4e+07 6e+07
Chr 20
0.00 0.24 0.48
0e+00 2e+07 4e+07
Chr 21
0.00 0.32 0.64
0e+00 2e+07 4e+07
Chr 22
0.00 0.28 0.56
0e+00 2e+07 4e+07
Chr 23
0.00 0.28 0.56
0e+00 2e+07 4e+07 6e+07
Chr 24
0.00 0.28 0.56
0e+00 2e+07 4e+07
Chr 25
0.00 0.24 0.48
0e+00 2e+07 4e+07
Chr 26
0.00 0.28 0.56
0e+00 2e+07 4e+07

MSpa

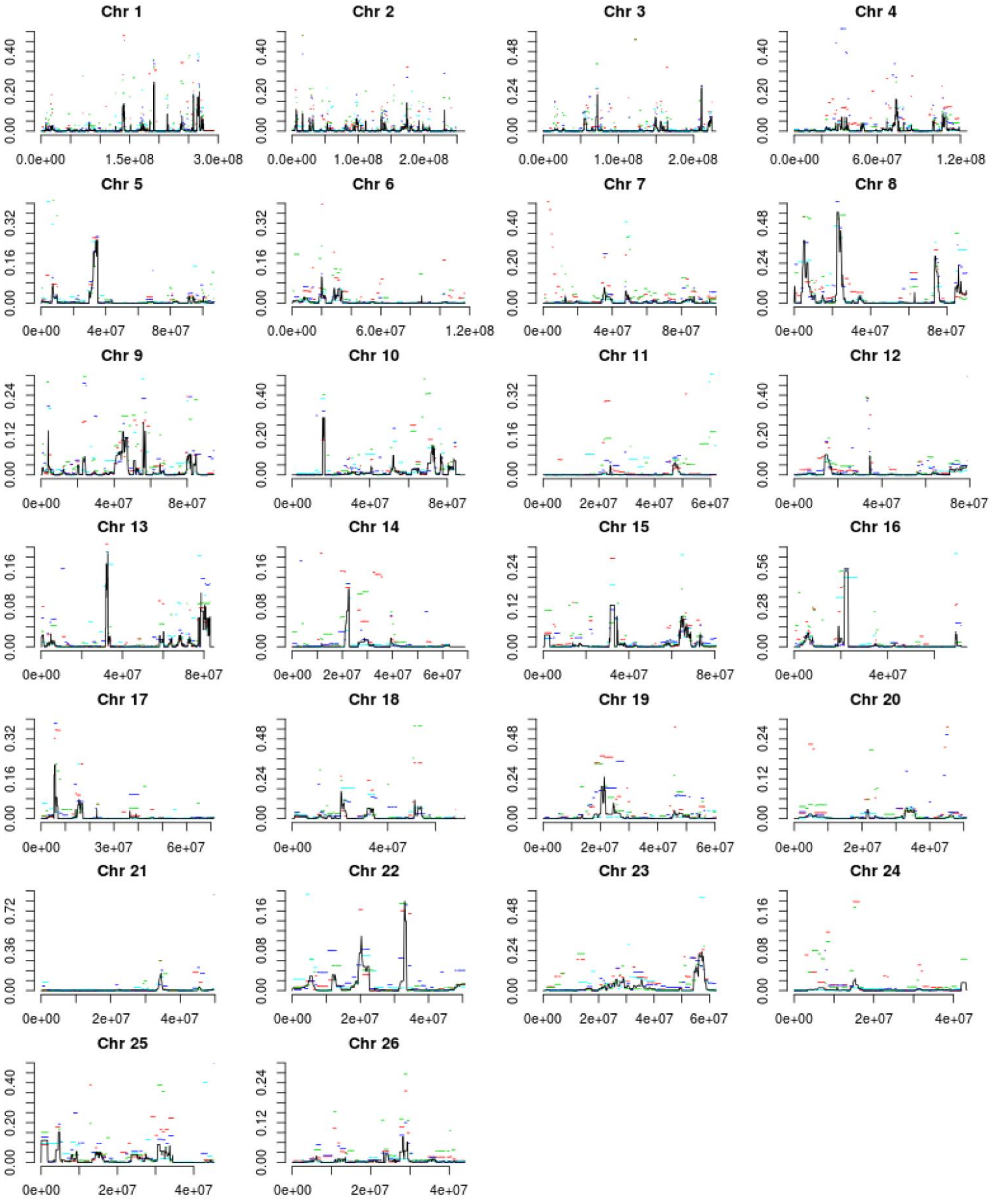
Chr 1
Chr 2
Chr 3
Chr 4
Chr 5
Chr 6
Chr 7
Chr 8
Chr 9
Chr 10
Chr 11
Chr 12
Chr 13
Chr 14
Chr 15
Chr 16
Chr 17
Chr 18
Chr 19
Chr 20
Chr 21
Chr 22
Chr 23
Chr 24
Chr 25
Chr 26

ALT

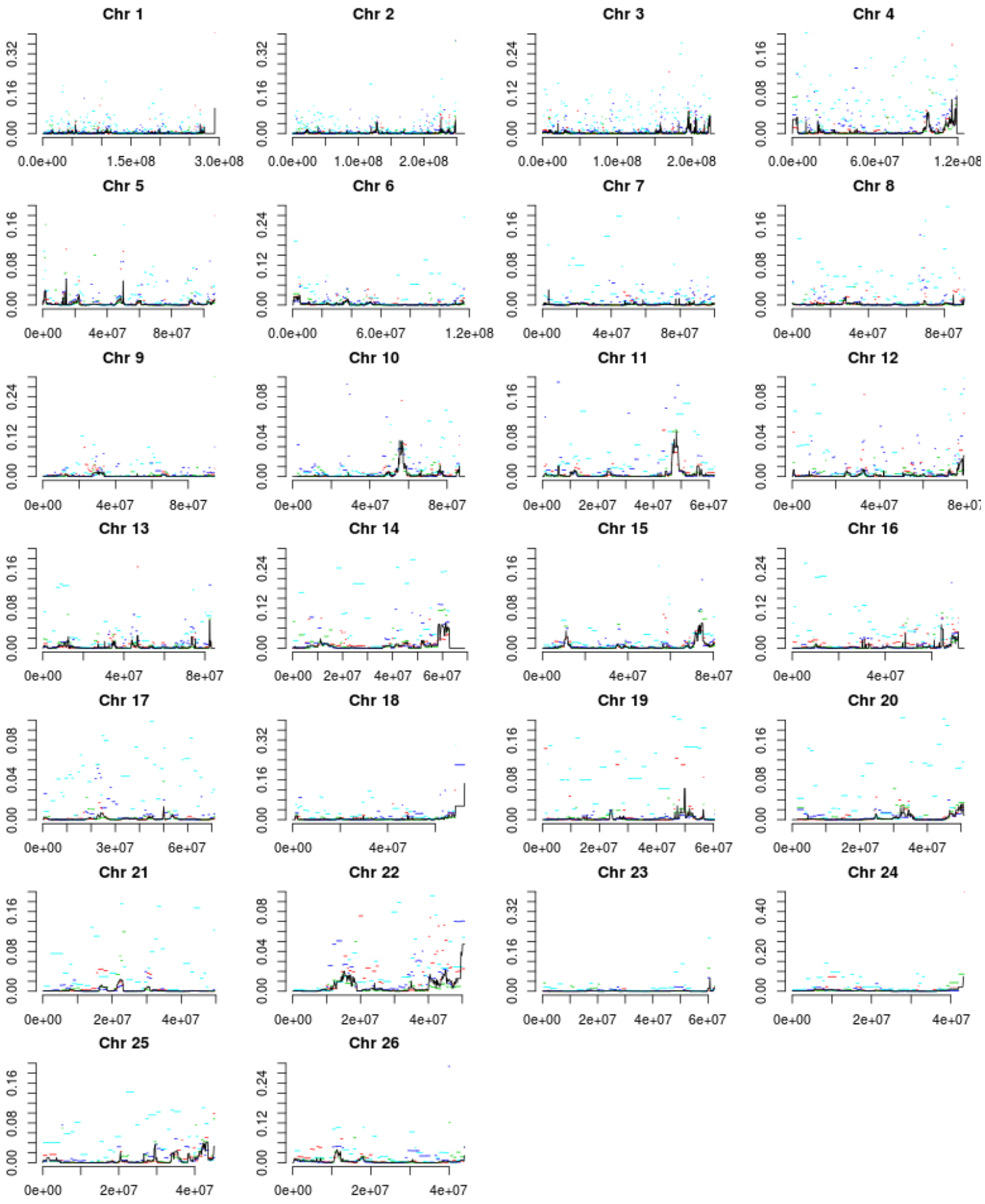
Chr 1
0.00 0.16 0.32
0.0e+00 1.5e+08 3.0e+08
Chr 2
0.00 0.16 0.32
0.0e+00 1.0e+08 2.0e+08
Chr 3
0.00 0.12 0.24
0.0e+00 1.0e+08 2.0e+08
Chr 4
0.00 0.08 0.16
0.0e+00 6.0e+07 1.2e+08
Chr 5
0.00 0.08 0.16
0e+00 4e+07 8e+07
Chr 6
0.00 0.12 0.24
0.0e+00 6.0e+07 1.2e+08
Chr 7
0.00 0.08 0.16
0e+00 4e+07 8e+07
Chr 8
0.00 0.08 0.16
0e+00 4e+07 8e+07
Chr 9
0.00 0.12 0.24
0e+00 4e+07 8e+07
Chr 10
0.00 0.04 0.08
0e+00 4e+07 8e+07
Chr 11
0.00 0.08 0.16
0e+00 2e+07 4e+07 6e+07
Chr 12
0.00 0.04 0.08
0e+00 4e+07 8e+07
Chr 13
0.00 0.08 0.16
0e+00 4e+07 8e+07
Chr 14
0.00 0.12 0.24
0e+00 2e+07 4e+07 6e+07
Chr 15
0.00 0.08 0.16
0e+00 4e+07 8e+07
Chr 16
0.00 0.12 0.24
0e+00 4e+07
Chr 17
0.00 0.04 0.08
0e+00 3e+07 6e+07
Chr 18
0.00 0.16 0.32
0e+00 4e+07
Chr 19
0.00 0.08 0.16
0e+00 2e+07 4e+07 6e+07
Chr 20
0.00 0.08 0.16
0e+00 2e+07 4e+07
Chr 21
0.00 0.08 0.16
0e+00 2e+07 4e+07
Chr 22
0.00 0.04 0.08
0e+00 2e+07 4e+07
Chr 23
0.00 0.16 0.32
0e+00 2e+07 4e+07 6e+07
Chr 24
0.00 0.20 0.40
0e+00 2e+07 4e+07
Chr 25
0.00 0.08 0.16
0e+00 2e+07 4e+07
Chr 26
0.00 0.12 0.24
0e+00 2e+07 4e+07

CHI

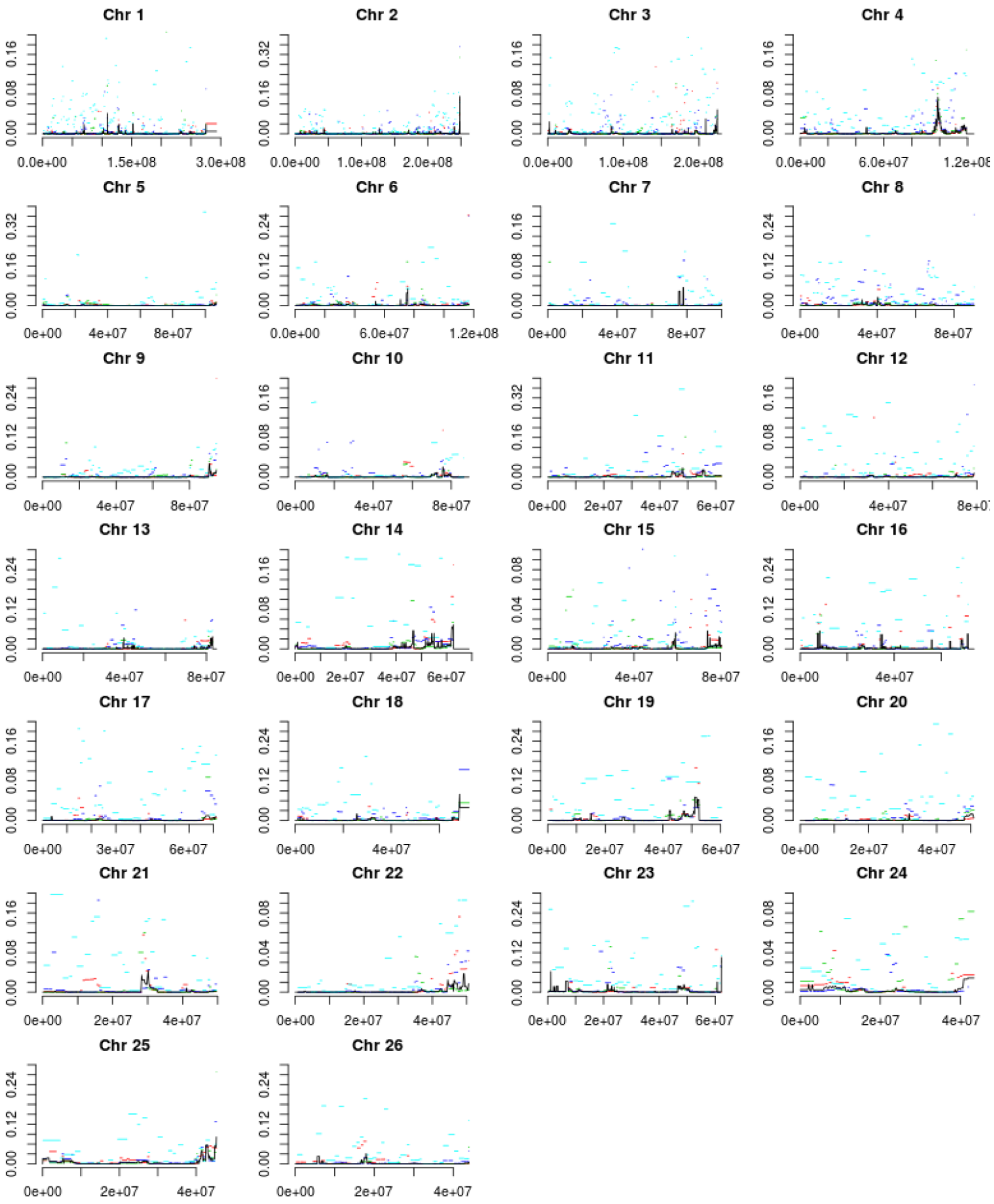

Chr 1
0.00 0.08 0.16
0.0e+00 1.5e+08 3.0e+08
Chr 2
0.00 0.16 0.32
0.0e+00 1.0e+08 2.0e+08
Chr 3
0.00 0.08 0.16
0.0e+00 1.0e+08 2.0e+08
Chr 4
0.00 0.08 0.16
0.0e+00 6.0e+07 1.2e+0
Chr 5
0.00 0.16 0.32
0e+00 4e+07 8e+07
Chr 6
0.00 0.12 0.24
0.0e+00 6.0e+07 1.2e+08
Chr 7
0.00 0.08 0.16
0e+00 4e+07 8e+07
Chr 8
0.00 0.12 0.24
0e+00 4e+07 8e+07
Chr 9
0.00 0.12 0.24
0e+00 4e+07 8e+07
Chr 10
0.00 0.08 0.16
0e+00 4e+07 8e+07
Chr 11
0.00 0.16 0.32
0e+00 2e+07 4e+07 6e+07
Chr 12
0.00 0.08 0.16
0e+00 4e+07 8e+0
Chr 13
0.00 0.12 0.24
0e+00 4e+07 8e+07
Chr 14
0.00 0.08 0.16
0e+00 2e+07 4e+07 6e+07
Chr 15
0.00 0.04 0.08
0e+00 4e+07 8e+07
Chr 16
0.00 0.12 0.24
0e+00 4e+07
Chr 17
0.00 0.08 0.16
0e+00 3e+07 6e+07
Chr 18
0.00 0.12 0.24
0e+00 4e+07
Chr 19
0.00 0.12 0.24
0e+00 2e+07 4e+07 6e+07
Chr 20
0.00 0.08 0.16
0e+00 2e+07 4e+07
Chr 21
0.00 0.08 0.16
0e+00 2e+07 4e+07
Chr 22
0.00 0.04 0.08
0e+00 2e+07 4e+07
Chr 23
0.00 0.12 0.24
0e+00 2e+07 4e+07 6e+07
Chr 24
0.00 0.04 0.08
0e+00 2e+07 4e+07
Chr 25
0.00 0.12 0.24
0e+00 2e+07 4e+07
Chr 26
0.00 0.12 0.24
0e+00 2e+07 4e+07

CHU

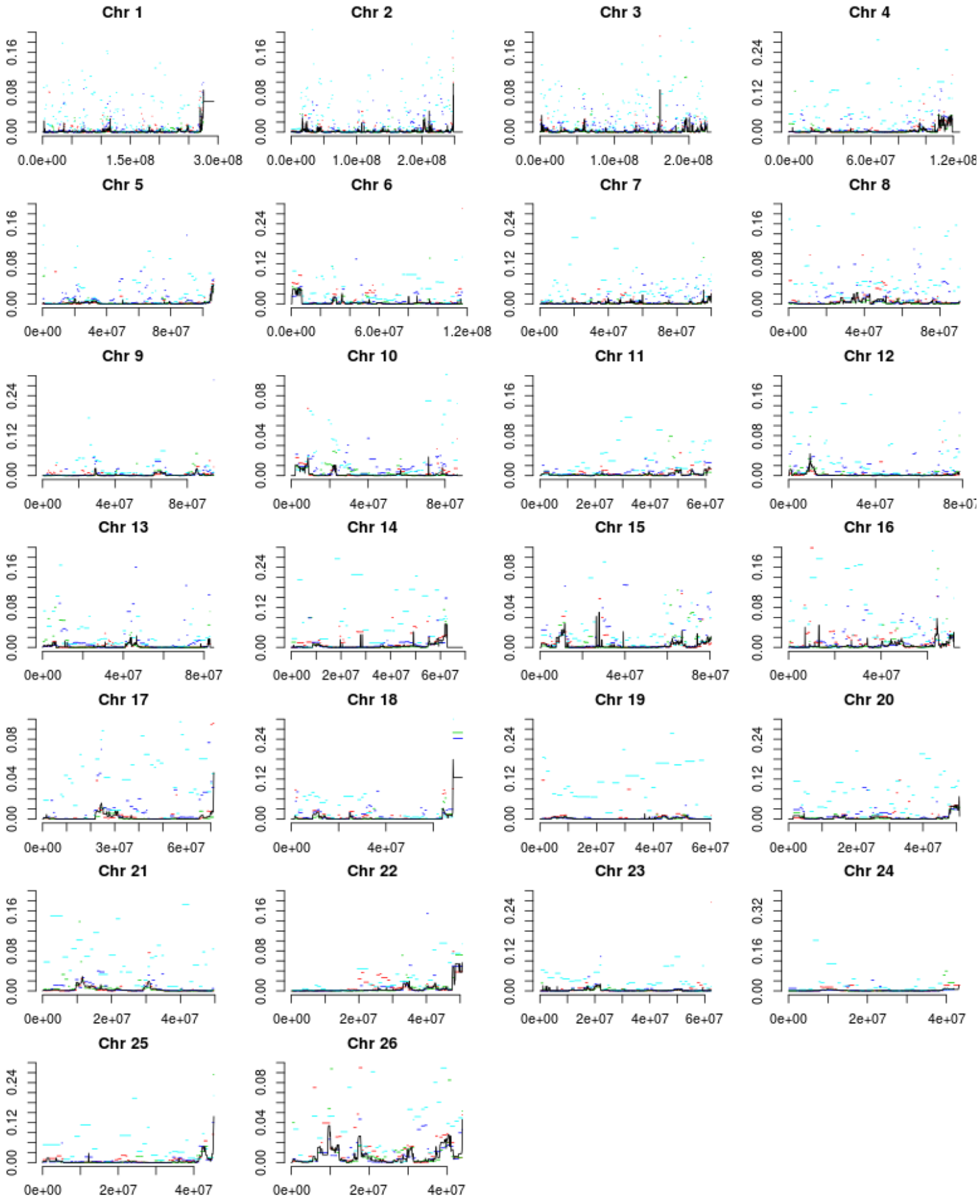
Chr 1
Chr 2
Chr 3
Chr 4
Chr 5
Chr 6
Chr 7
Chr 8
Chr 9
Chr 10
Chr 11
Chr 12
Chr 13
Chr 14
Chr 15
Chr 16
Chr 17
Chr 18
Chr 19
Chr 20
Chr 21
Chr 22
Chr 23
Chr 24
Chr 25
Chr 26

COM

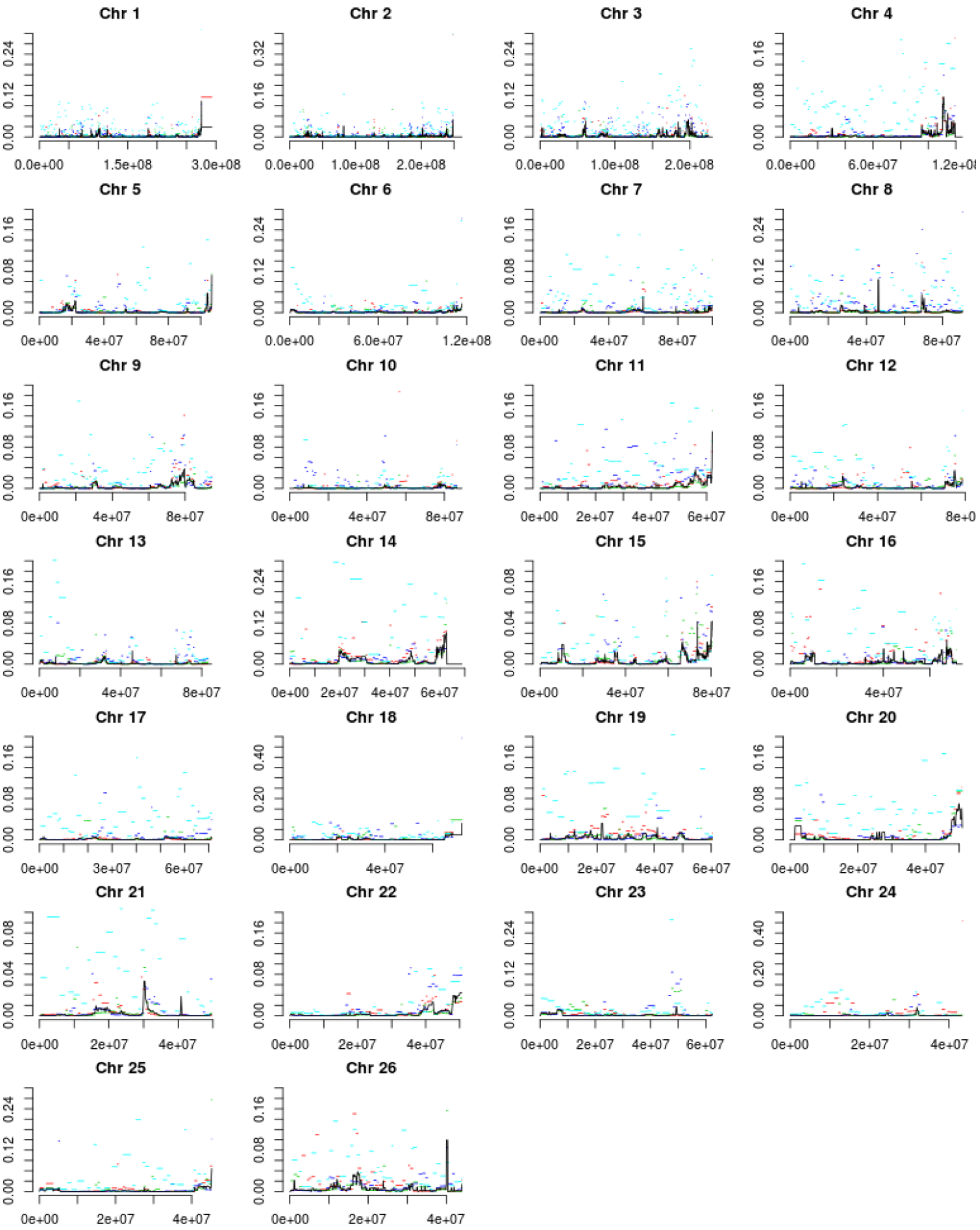
Chr 1
Chr 2
Chr 3
Chr 4
Chr 5
Chr 6
Chr 7
Chr 8
Chr 9
Chr 10
Chr 11
Chr 12
Chr 13
Chr 14
Chr 15
Chr 16
Chr 17
Chr 18
Chr 19
Chr 20
Chr 21
Chr 22
Chr 23
Chr 24
Chr 25
Chr 26

CFT

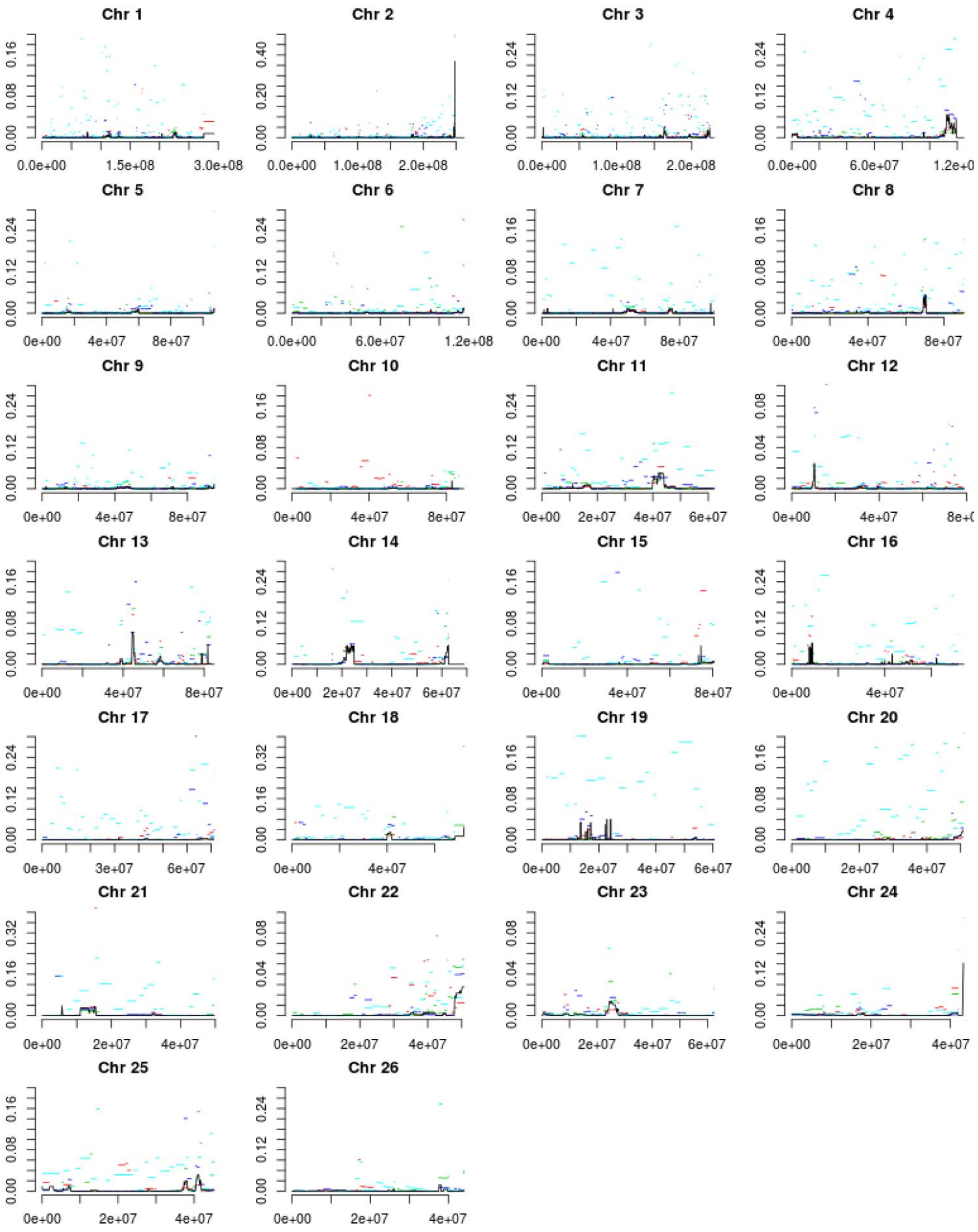
Chr 1
Chr 2
Chr 3
Chr 4
Chr 5
Chr 6
Chr 7
Chr 8
Chr 9
Chr 10
Chr 11
Chr 12
Chr 13
Chr 14
Chr 15
Chr 16
Chr 17
Chr 18
Chr 19
Chr 20
Chr 21
Chr 22
Chr 23
Chr 24
Chr 25
Chr 26

RAK

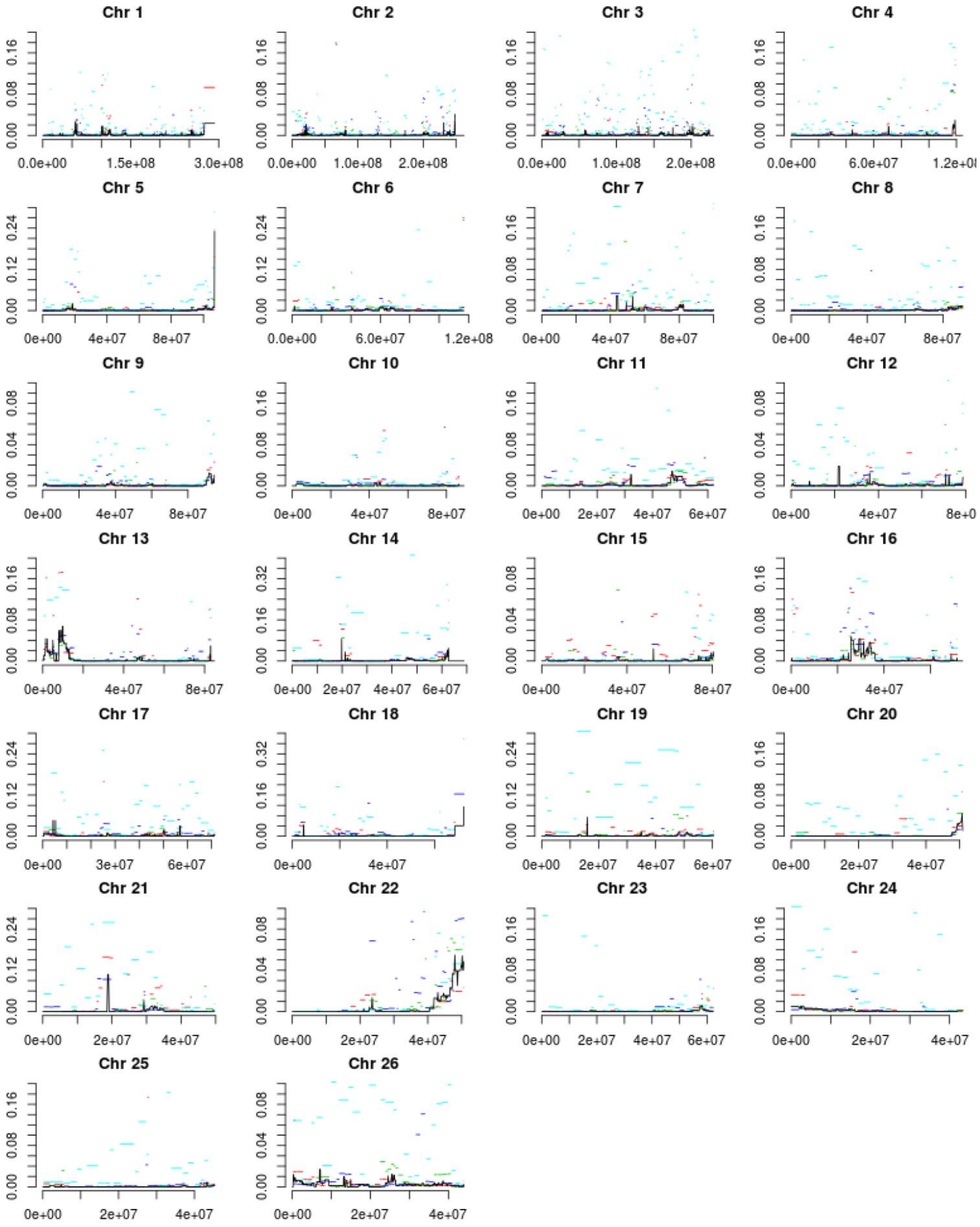
Chr 1
Chr 2
Chr 3
Chr 4
Chr 5
Chr 6
Chr 7
Chr 8
Chr 9
Chr 10
Chr 11
Chr 12
Chr 13
Chr 14
Chr 15
Chr 16
Chr 17
Chr 18
Chr 19
Chr 20
Chr 21
Chr 22
Chr 23
Chr 24
Chr 25
Chr 26

SAR

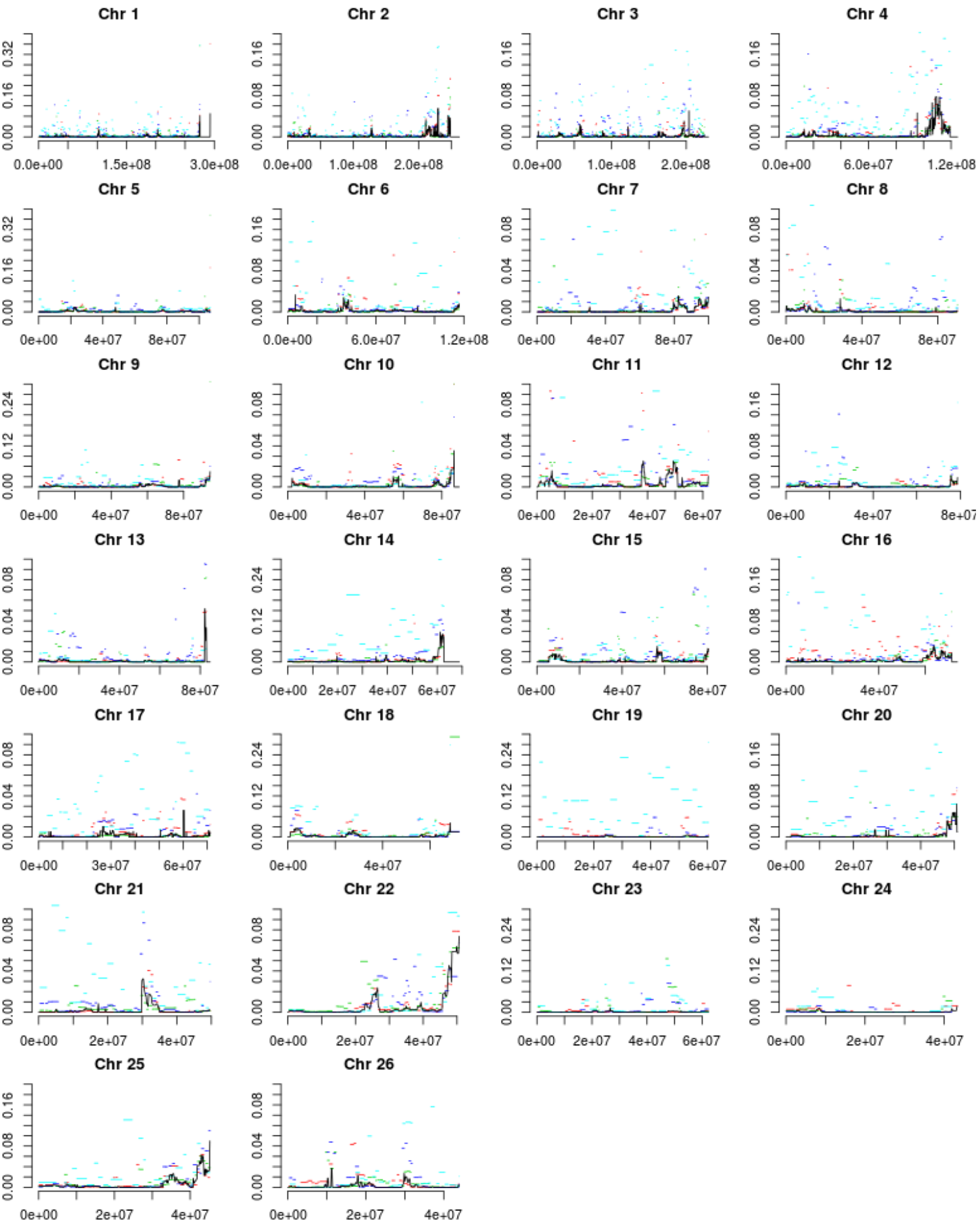
Chr 1
Chr 2
Chr 3
Chr 4
Chr 5
Chr 6
Chr 7
Chr 8
Chr 9
Chr 10
Chr 11
Chr 12
Chr 13
Chr 14
Chr 15
Chr 16
Chr 17
Chr 18
Chr 19
Chr 20
Chr 21
Chr 22
Chr 23
Chr 24
Chr 25
Chr 26

SBF

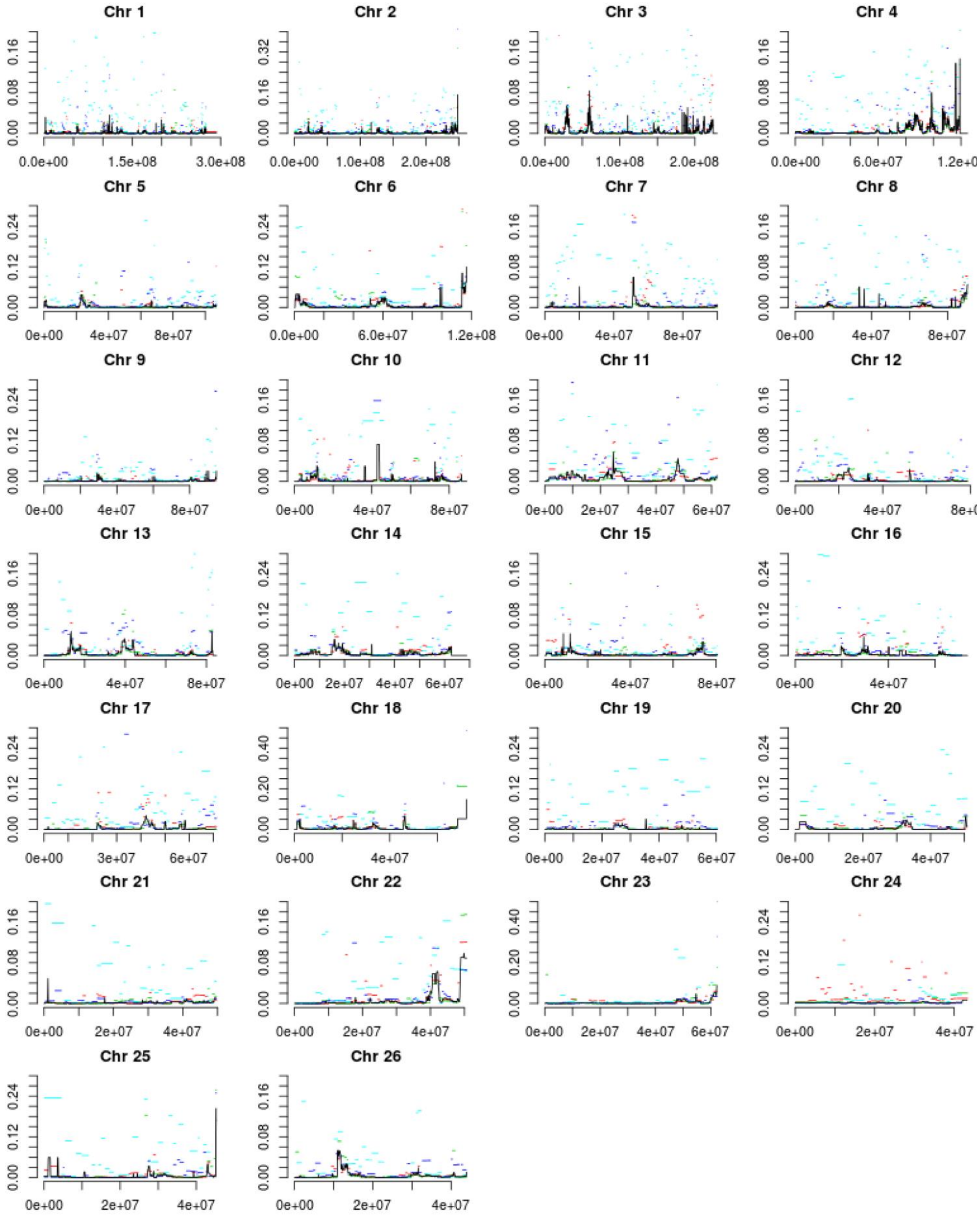
Chr 1
Chr 2
Chr 3
Chr 4
Chr 5
Chr 6
Chr 7
Chr 8
Chr 9
Chr 10
Chr 11
Chr 12
Chr 13
Chr 14
Chr 15
Chr 16
Chr 17
Chr 18
Chr 19
Chr 20
Chr 21
Chr 22
Chr 23
Chr 24
Chr 25
Chr 26

SOA

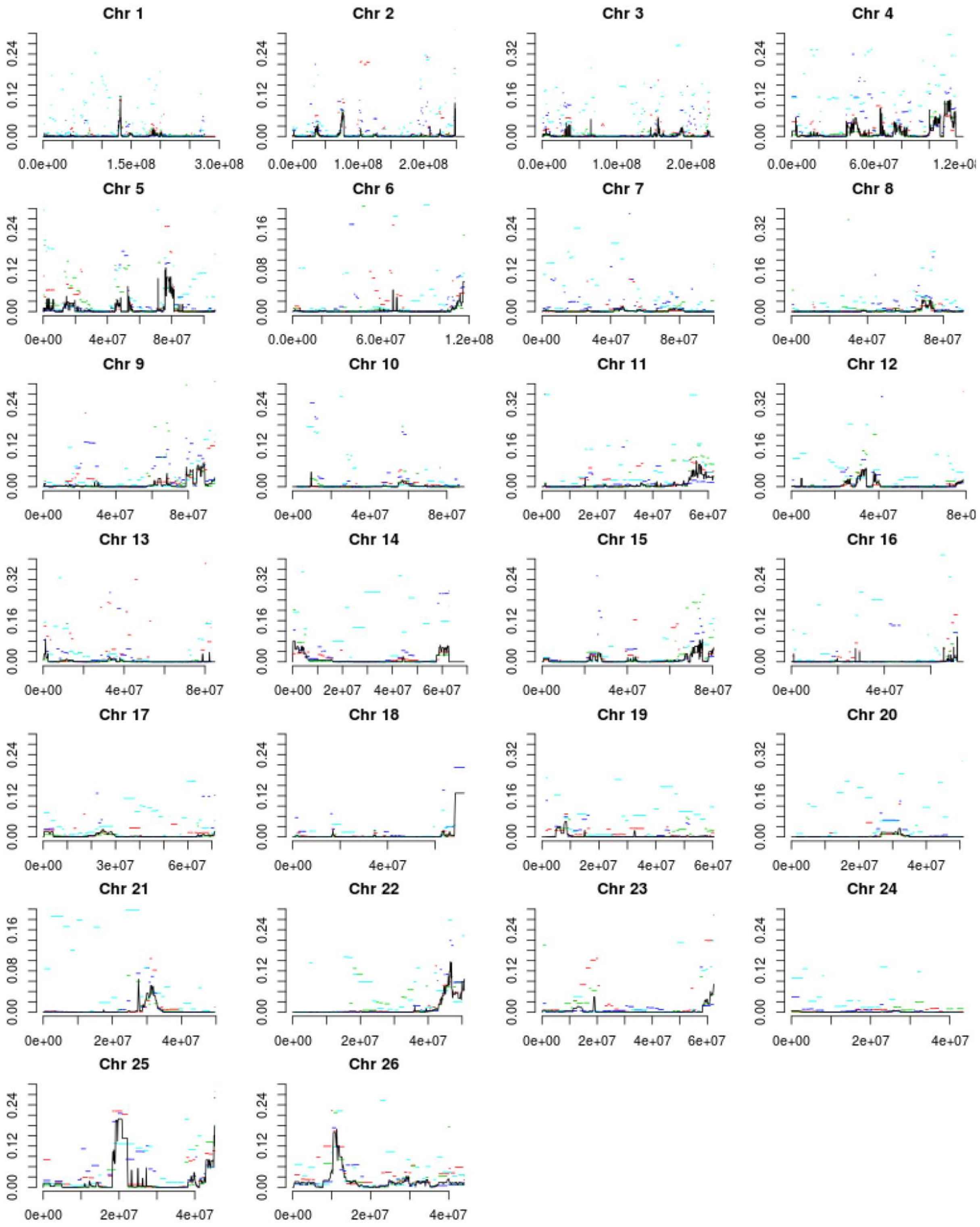

Chr 1
Chr 2
Chr 3
Chr 4
Chr 5
Chr 6
Chr 7
Chr 8
Chr 9
Chr 10
Chr 11
Chr 12
Chr 13
Chr 14
Chr 15
Chr 16
Chr 17
Chr 18
Chr 19
Chr 20
Chr 21
Chr 22
Chr 23
Chr 24
Chr 25
Chr 26

SPW

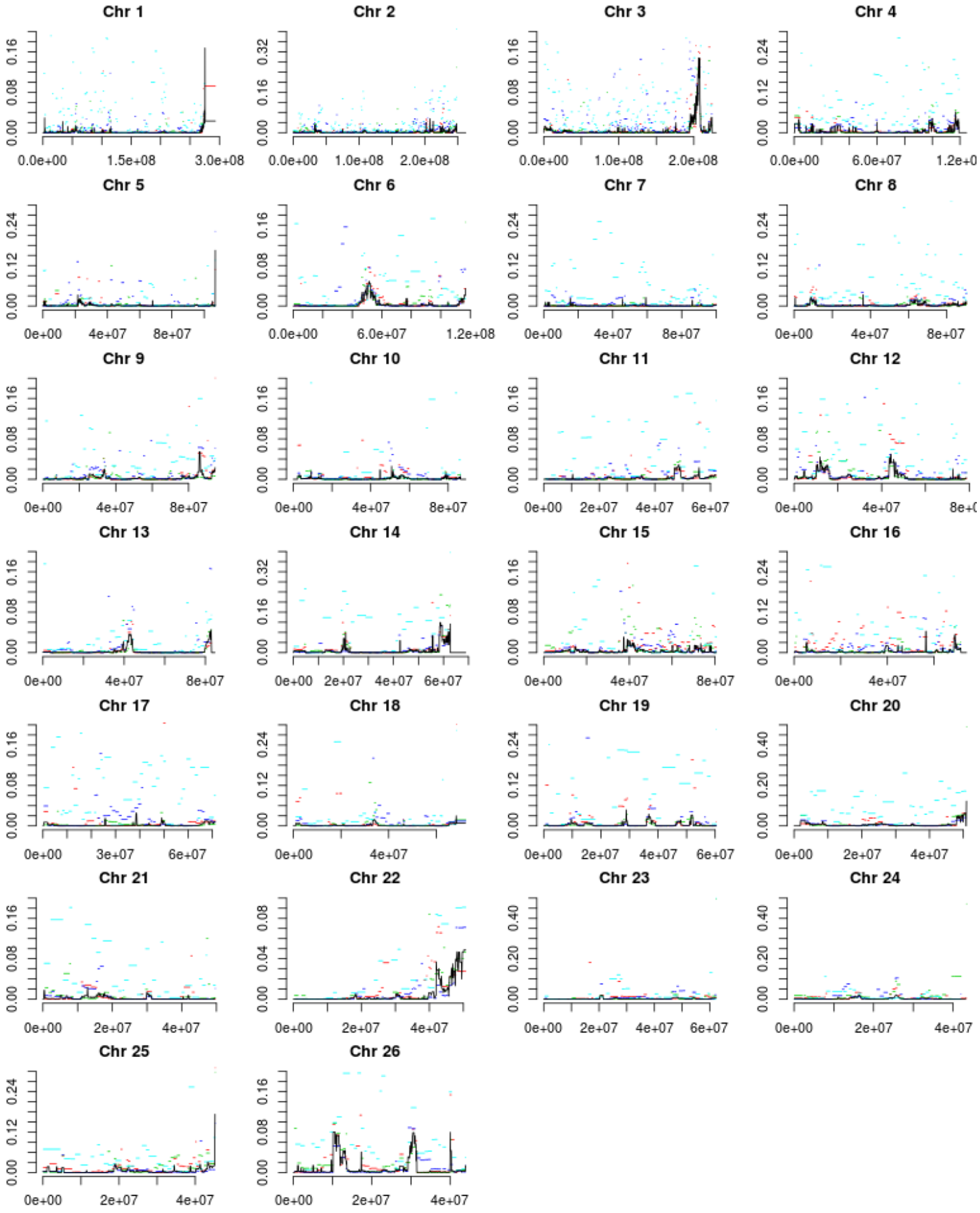
Chr 1
Chr 2
Chr 3
Chr 4
Chr 5
Chr 6
Chr 7
Chr 8
Chr 9
Chr 10
Chr 11
Chr 12
Chr 13
Chr 14
Chr 15
Chr 16
Chr 17
Chr 18
Chr 19
Chr 20
Chr 21
Chr 22
Chr 23
Chr 24
Chr 25
Chr 26

VBN

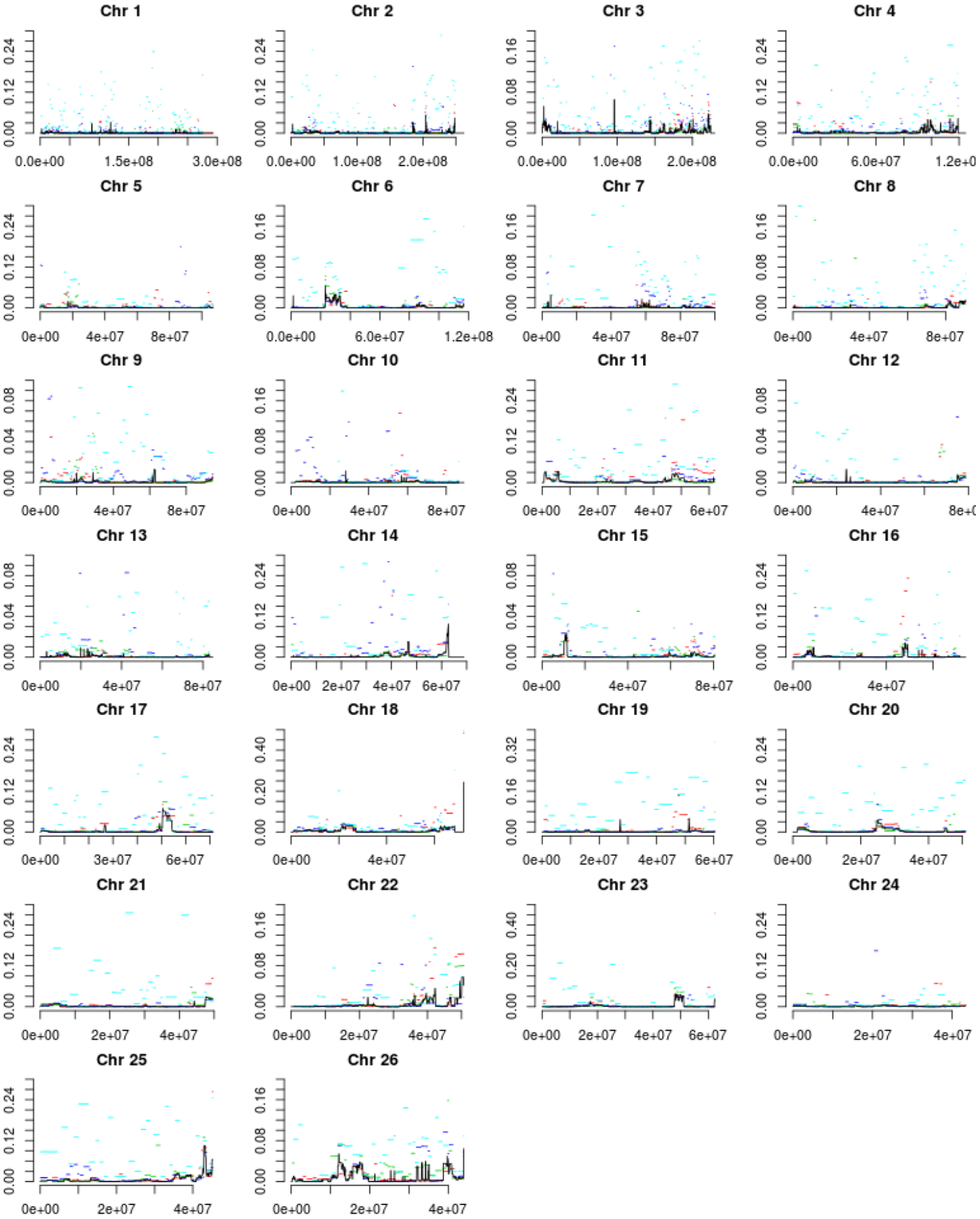
Chr 1
Chr 2
Chr 3
Chr 4
Chr 5
Chr 6
Chr 7
Chr 8
Chr 9
Chr 10
Chr 11
Chr 12
Chr 13
Chr 14
Chr 15
Chr 16
Chr 17
Chr 18
Chr 19
Chr 20
Chr 21
Chr 22
Chr 23
Chr 24
Chr 25
Chr 26
